# Photoredox-catalyzed intramolecular nucleophilic amidation of alkenes with β-lactams

**DOI:** 10.3762/bjoc.20.210

**Published:** 2024-10-01

**Authors:** Valentina Giraldi, Giandomenico Magagnano, Daria Giacomini, Pier Giorgio Cozzi, Andrea Gualandi

**Affiliations:** 1 Department of Chemistry “G. Ciamician”, ALMA MATER STUDIORUM - Università di Bologna, Via Gobetti 85, 40129 Bologna, Italy; 2 Center for Chemical Catalysis - C3, ALMA MATER STUDIORUM - Università di Bologna, Via Gobetti 85, 40129 Bologna, Italy

**Keywords:** β-lactam, acridinium photocatalyst, alkenes, amides, intramolecular radical reaction, photoredox catalysis

## Abstract

The direct nucleophilic addition of amides to unfunctionalized alkenes via photoredox catalysis represents a facile approach towards functionalized alkylamides. Unfortunately, the scarce nucleophilicity of amides and competitive side reactions limit the utility of this approach. Herein, we report an intramolecular photoredox cyclization of alkenes with β-lactams in the presence of an acridinium photocatalyst. The approach uses an intramolecular nucleophilic addition of the β-lactam nitrogen atom to the radical cation photogenerated in the linked alkene moiety, followed by hydrogen transfer from the hydrogen atom transfer (HAT) catalyst. This process was used to successfully prepare 2-alkylated clavam derivatives.

## Introduction

Access to nitrogen radicals for the functionalization of alkenes is a field under active investigation [1–4], as it gives the possibility to directly introduce nitrogen into an alkyl chain (alkene carboamination) to obtain valuable nitrogen-containing molecules [5–6]. Among several N-centered radicals, such as aminyl, amidyl, or iminyl radicals, N-heterocyclic amidyl radicals were largely underinvestigated despite their importance as intermediates or relevant N-heterocyclic products in medicinal chemistry [7–10].

**Figure 1:**
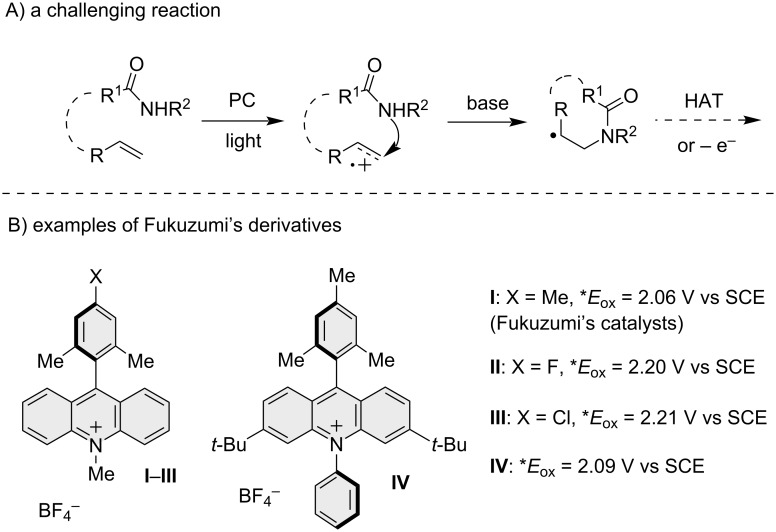
A) Photoredox amidocyclization reaction. B) The strongly oxidizing Fukuzumi catalyst (**I**) used in the functionalization of alkenes by amides, and more recent variants.

Recently, photoredox catalysis has emerged as a novel area of research [11–12], particularly focusing on innovative approaches to synthesize natural or bioactive compounds [13]. In the carboamination of alkenes, amides are used in photoredox cyclizations under proton-coupled electron transfer (PCET) conditions [14–17]. An alternative method to generate *N*-amidyl radicals uses activated N–O amide derivatives capable of generating amidyl radicals through fragmentation [18–19]. The direct formation of amidyl radicals in the presence of a carbon alkyl chain could lead to a competitive 1,5-hydrogen atom transfer (1,5-HAT) [20–22], limiting the direct functionalization of amides with alkenes under photoredox conditions. Another viable approach for amide functionalization through photoredox catalysis involves the nucleophilic addition, in the presence of base, of an amide to a radical cation obtained by oxidation of an unfunctionalized alkene moiety (Figure 1A) [23–25]. The nucleophilic attack of the nitrogen atom on the oxidized C=C double bond results in the formation of a radical intermediate after deprotonation. This radical intermediate can proceed through various pathways (e.g., HAT, oxidation) to yield the desired final product.

In the functionalization of amides with alkenes under oxidative conditions, the oxidation potential of the alkene plays a pivotal role in the oxidation to a radical cation through photoredox catalysis [26]. Alkenes that are less functionalized possess a higher oxidation potential, necessitating the use of potent photocatalysts (PC) that act as oxidants in the excited state [27]. The direct functionalization of amides with alkenes has been a relatively underexplored area in research, as evidenced by the limited number of examples reported in the literature. An interesting observation was made by the Nicewicz group during their investigation of the hydrofunctionalization reaction of unsaturated amides and thioamides [28]. They have found that the oxygen atom of the amide group, rather than the nitrogen atom, acted as a nucleophile, leading to the formation of 2-oxazolines and 2-thiazolines. Another recent example of intramolecular nucleophilic attack induced by photocatalytic oxidation was reported by Yoon et al. with tosylamide derivatives [29]. Specifically, amides were employed in a photoredox cyclization process using a strong photooxidative acridinium catalyst such as the Fukuzumi catalyst (**I**, Figure 1B) [30–31]. Through tailored molecular design, it is possible to enhance the oxidation capability of these catalysts, enabling the utilization of less reactive alkenes and even aromatic molecules such as toluene [32].

**Figure 2:**
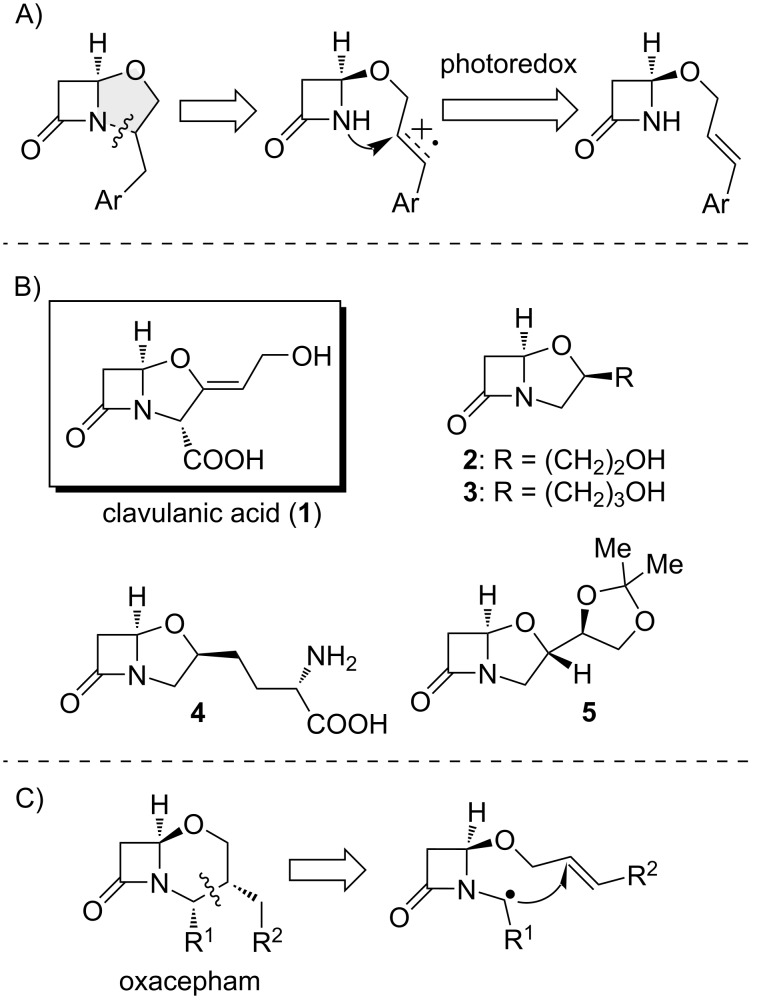
A) Access of clavam derivatives by intramolecular photoredox reaction of alkenes. B) Clavulanic acid and its derivatives. C) Construction of the oxacepham scaffold by radical cyclization.

Until now, heterocyclic amides such as β-lactam compounds have not been employed in alkene carboaminations. However, photoredox catalysis could be applied to a suitable β-lactam intermediate decorated with an alkene moiety to achieve N–H addition and cyclization to the fused bicyclic system of clavams (Figure 2A).

Clavulanic acid (**1**, Figure 2B) belongs to the family of clavam β-lactam compounds and is well known as a potent β-lactamase inhibitor [33–34]. It is produced by the filamentous bacterium *Streptomyces clavuligerus,* but in low yield. Various clavams **2**–**5** have been identified (Figure 2B), either through isolation as natural metabolites or obtained by synthetic methods [35–43]. The inhibitory activity of β-lactamases is exhibited by those congeners with a (3*R*,5*R*)-configuration, such as clavulanic acid (**1**), whereas clavams with other configurations are not lactamase inhibitors, although some of these have antifungal or antibacterial properties [35]. In the literature, oxacepham scaffolds, the 6-membered fused bicyclic analog of clavams, were prepared from appropriately substituted unfused precursors by intramolecular C-radical addition to alkene functionalities [44]. The utilization of radical conditions has prevented the effective nucleophilic opening of the lactams.

Notably, all reported structures have alkyl or aryl substituents in position 3 of the clavam ring. Conversely, clavams substituted with alkyl chains at the 2-position, to the best of our knowledge, have not been previously reported and are absent from common organic compound databases. To explore potential biological effects, a simple and modular approach to these molecules is thus required. Based on this, we decided to use photoredox chemistry to access 2-alkylclavams through a simplified synthetic pathway. We investigated the intramolecular nucleophilic addition of the nitrogen atom of the β-lactams to photooxidized alkenes (Figure 2A), and our findings are presented in this study.

## Results and Discussion

**Scheme 1:**
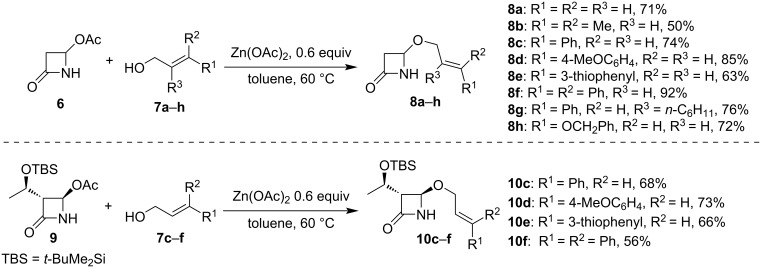
Preparation of alkenyl β-lactam derivatives for the intramolecular photoredox reaction.

The initial phase of our investigation involved the synthesis of suitable starting compounds for the following oxidative cyclization. For this purpose, a series of 4-alkoxy-β-lactams containing an alkene group was readily synthesized starting from commercially available 4-acetoxy-2-azetidinone (**6**) by nucleophilic displacement of the 4-acetoxy group with allylic alcohols promoted by Zn(OAc)_2_ (see compounds **8a**–**h**, Scheme 1) [45]. Similarly, enantiopure derivatives **10c**–**f** were synthesized from the commercially available β-lactam **9**, a key intermediate for the industrial preparation of carbapenems.

Starting from the reaction conditions reported by Nicewicz and Morse [28], we optimized the conditions with compound **8c** as the model substrate for the photoredox cyclization (Table 1). The reaction was carried out in DCM with acridinium PC **IV** (5 mol %), 50 mol % of PhSSPh as HAT catalyst, and lutidine (50 mol %) as the base.

**Table 1:** Intramolecular cyclization of β-lactams induced by photoredox conditions.^a^

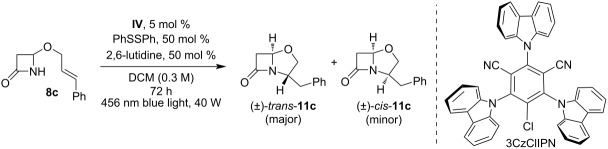			

entry	deviation from standard conditions	conversion to **11c**, %^b,c^	dr^d^

1	—	72 (70)	1.4:1
2	absence of **IV**	0	—
3	absence of light	0	—
4	reaction time 14 h	traces	—
5	20 mol % PhSSPh, 20 mol % 2,6-lutidine	49	1.4:1
6	DMF solvent	0	—
7	MeCN solvent	68	1.4:1
8	DCE solvent	49	1.4:1
9	**I** instead of **IV**	60	1.4:1
10	3CzClIPN instead of **IV**	0	—

^a^The reactions were conducted under irradiation with a Kessil blue light (40 W) for 72 hours on a 0.05 mmol scale. ^b^Conversion determined by ^1^H NMR analysis of the crude reaction mixture. ^c^In parentheses: isolated yield after purification by flash column chromatography. ^d^*Trans*/*cis* dr determined by ^1^H NMR analysis on the crude reaction mixture.

Upon 72 hours of irradiation with a blue light at 456 nm, the product **11c** was obtained in a satisfactory yield as a mixture of diastereoisomers in a 1.4:1 ratio (Table 1, entry 1). Assignment of the relative configurations as *cis* or *trans* was achieved by ^1^H NMR analysis, considering the chemical shifts of the proton in the α-position of the β-lactam nitrogen atom and the geminal protons in the benzylic position (see Supporting Information File 1). The difference in the chemical shifts of these protons in the two isomers could be attributed to the influence of the anisotropy of the neighboring carboxy group of the β-lactam and could be correlated with the configuration at the bridgehead stereocenter [46]. This analysis revealed the preferred formation of the *trans*- over the *cis*-isomer.

The optimal PC for the reaction was acridinium salt **IV** (Table 1, entry 1), while the Fukuzumi catalyst (**I**), commonly employed by Nicewicz et al., was less effective (Table 1, entry 9). 3CzClIPN, an organic dye belonging to the class of thermally activated delayed fluorescence (TADF) dyes commonly employed nowadays in photoredox catalysis [47], was tested in our reaction. This dye was chosen due to its oxidizing properties, and it ranks among the most oxidizing agents within this class of compounds (*E*_1/2_[*PC/PC^•−^] = +1.56 V vs SCE) [48], but it proved ineffective in our reaction (Table 1, entry 10). The reaction was successfully conducted in various solvents, such as DCM, DCE, and MeCN (Table 1, entries 1, 7, and 8). However, DMF failed to yield the desired product (Table 1, entry 6). Shortening the reaction time to 14 hours resulted in minimal product formation (Table 1, entry 4), while reducing the amount of PhSSPh and lutidine to 20 mol % led to a lower yield (Table 1, entry 5).

**Scheme 2:**
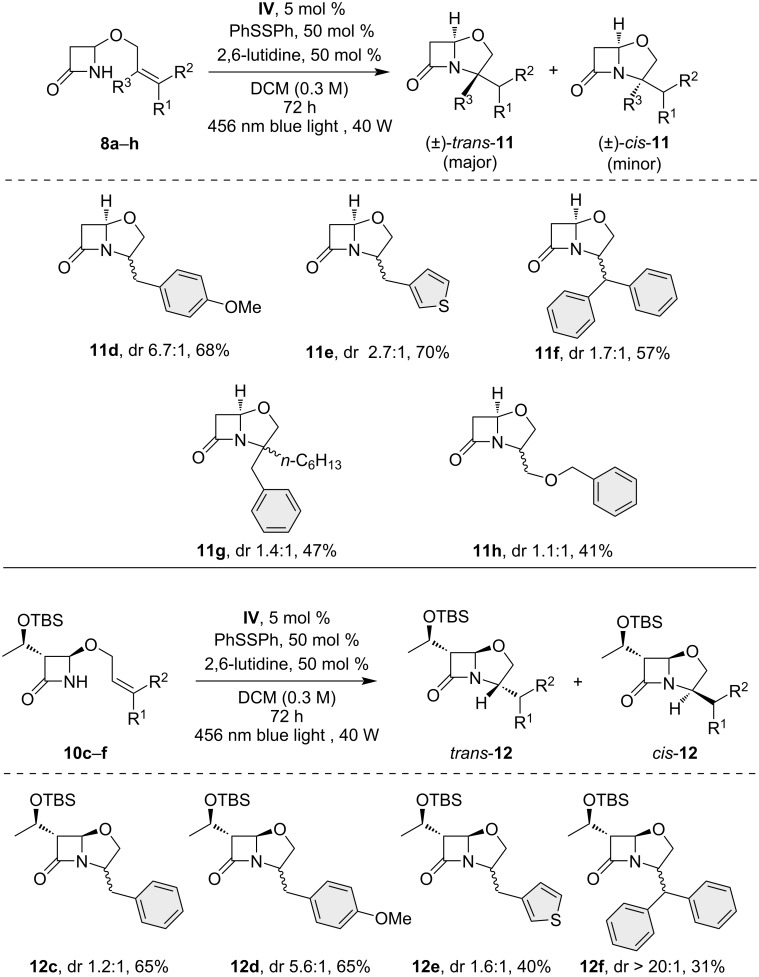
Photoredox-catalyzed intramolecular N-alkylation reactions of various β-lactams. The *trans*/*cis* dr was determined by ^1^H NMR analysis of the crude reaction mixture.

With the optimized reaction conditions in hand, we submitted the previously prepared 4-alkoxy-β-lactam substrates **8a**–**h** to photoredox conditions (Scheme 1), and the salient results are reported in Scheme 2. Unfortunately, the substrates **8a**,**b** displayed low reactivity due to their significantly higher oxidation potential compared to the excited photoredox catalysts (>2.5 V vs SCE) [49]. However, other derivatives exhibited a satisfactory product yield ranging from moderate to good. Substrate **8d** exhibited an enhancement in reaction diastereoselectivity, resulting in the isolation of product **11d** with a dr of 6.7:1 in favor of the *trans*-diastereoisomer. Remarkably, the formation of a fully substituted quaternary center was possible, as observed for the product **11g**, where the *trans*-diastereoisomer was favored.

In this study, enantiopure 3-(1’-(*tert*-butyldimethylsilyl)ethyl)-β-lactams **10c**–**f** were also tested. Products **12c**–**f** were obtained with moderate to good yield, underscoring the feasibility of the methodology for the C3-substituted β-lactam moiety. The configuration at the newly formed stereocenter in the five-membered ring was attributed by ^1^H NMR analysis for **11c**. Moreover, the configuration was also confirmed by NOE studies on the two isolated diastereoisomers, which confirmed the preferred *trans*-isomer formation (see Supporting Information File 1).

Analysis of the dr values revealed that the diastereoselectivity of the nucleophilic attack of the β-lactam on the radical cationic intermediate was influenced by stereoelectronic factors. Compounds unsubstituted at position C-3 of the β-lactam ring, **11d**–**h**, showed modest stereoselectivity with a higher dr (6.7:1) for compound **11d**, which has an electron-donating methoxy group on the phenyl substituent. Derivatives with a 3-OTBS side chain, **12c**–**e**, displayed moderate diastereoselectivity, except for the higher diastereoselectivity achieved for **12f** (dr 20:1), probably due to steric effects, albeit at the expense of a reduced isolated yield.

Across all tested substrates, nucleophilic attack predominantly occurred at the homobenzylic position, leading to the regioselective formation of clavam derivative with 2-benzylic substitution due to aryl stabilization of the radical intermediate (see mechanistic discussion below).

**Scheme 3:**
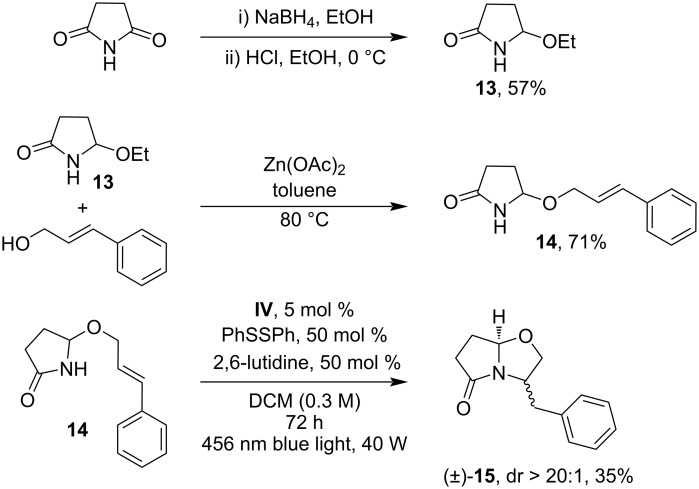
Synthesis of the model substrate **14** and its photoredox-catalyzed intramolecular N-alkylation reaction. The *trans*/*cis* dr was determined by ^1^H NMR analysis of the crude reaction mixture.

We briefly investigated whether this protocol could be adapted to other lactams, allowing for a practical synthesis of bicyclic structures. The resulting bicyclic lactam substrate could serve as a foundation to access pyrroloisoquinoline alkaloids [50–51]. The model substrate **14** was synthesized in a two-step process starting from succinimide (Scheme 3). Through a simple reaction in toluene at 80 °C in the presence of Zn(OAc)_2_, the hemiaminal derivative **13** underwent substitution with cinnamyl alcohol, resulting in the isolation of **14** with a satisfactory yield. Under optimized reaction conditions, the photocatalytic cyclization occurred by producing *trans*-**15** in 35% yield as the single diastereoisomer.

**Figure 3:**
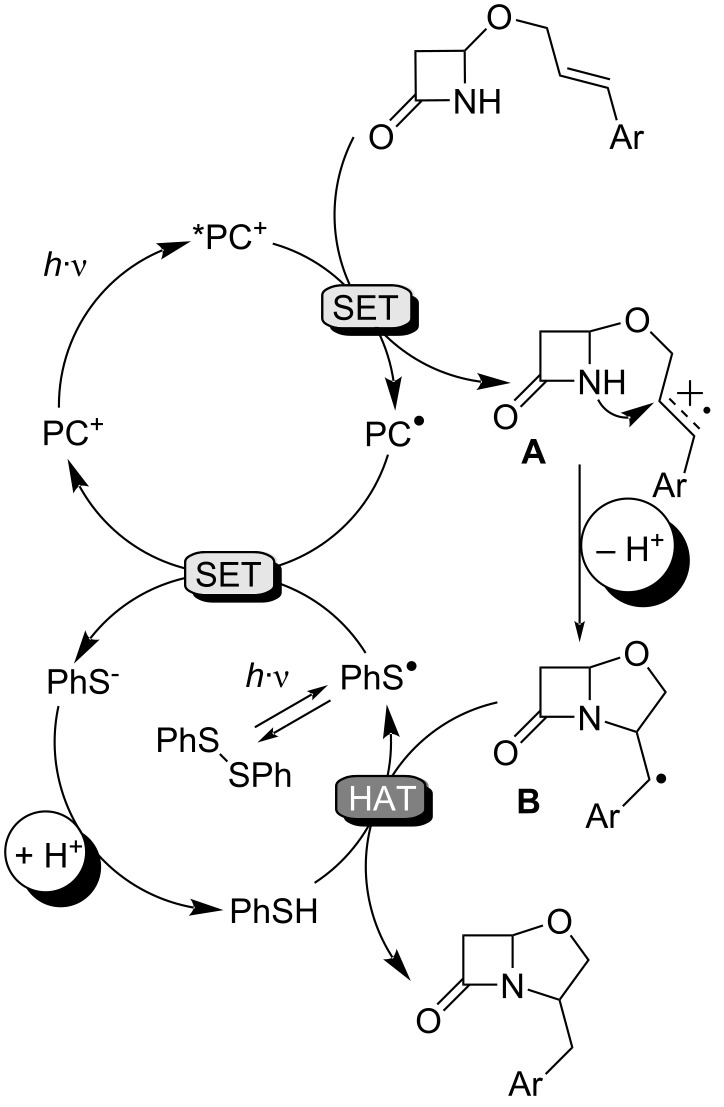
Tentative mechanism for the photo-cyclization reaction.

For the reaction mechanism, we propose a mechanistic hypothesis according to the study by Nicewicz and Nguyen (Figure 3) [23]. The incorporation of electron-donating groups into the acridinium core, as in catalyst **IV**, enhances charge transfer by stabilizing the mesityl moiety. Conversely, the introduction of *tert-*butyl groups increases the life time of the excited state [52–56]. As a consequence, the PC **IV** is a strong oxidant in the excited state and displays unique oxidizing properties (*E*_1/2_[*PC^+^/PC^•^] = +2.09 V vs SCE) [55–56]. The *PC^+^ species can oxidize the unsaturated lactam, thereby producing the corresponding radical cation intermediate **A**. The low stabilization by amide-bond resonance of the cyclic four-membered β-lactam [57–58] ensures a good nucleophilicity of the nitrogen atom to efficiently attack the radical cation **A**, giving the bicyclic radical intermediate **B**. Amide is an ambident nucleophile, and oxygen attack of radical cation **A** is also conceivable to give the corresponding imidate [28]. In our reaction, O-addition is disfavored due to the formation of an unsaturated four-membered ring as final product, characterized by significant ring strain [59]. Under light irradiation, PhSSPh is in equilibrium with the corresponding thiyl radical, which is subsequently reduced to thiophenolate by PC^•^, originating from the reduction of *PC^+^. The reduction potential of PhS^−^/PhS^•^ (*E*p_red_ = +0.45 V vs SCE) [60–61] is sufficient to oxidize the radical form of **IV** (PC^•^/PC^+^ = −0.57 V vs SCE) [55–56]. Finally, PhS^–^ is protonated and HAT from thiophenol to **B** furnishes the final product, closing the HAT cycle. Additionally, lutidine acts as a proton shuttle between the lactam NH unit and thiophenolate.

## Conclusion

To conclude, we have employed a photoredox methodology to access clavam and pyrrolyloxazole intermediates, showing the possibility of using the nucleophilic nitrogen atom of β-lactams under photoredox conditions. The acridinium catalyst **IV** was able to oxidize the C=C double bond present in the substrates to access the corresponding radical cation. The reaction shows high regioselectivity and good to moderate diastereoselectivity with satisfactory yield. The limitation, which will be further addressed by a more powerful catalyst, is related to the unreactivity of unsubstituted alkenes due to their higher oxidation potential. Biological studies concerning the new derivatives will also be a subject of future investigations.

## Supporting Information

File 1 Reaction optimization studies, general experimental procedures, product isolation and characterization, spectroscopic data for new compounds, and copies of NMR spectra.

## Data Availability

All data that supports the findings of this study is available in the published article and/or the supporting information of this article.
