# Supplementary material for: Photoredox-catalyzed intramolecular nucleophilic amidation of alkenes with β-lactams
Source: Beilstein J Org Chem. 2024 Oct 1;20:2461–8. doi: 10.3762/bjoc.20.210 (PMC11457124; doi:10.3762/bjoc.20.210)

BEILSTEIN JOURNAL OF ORGANIC CHEMISTRY

Supporting Information

for

Photoredox-catalyzed intramolecular nucleophilic amidation of alkenes with β-lactams

Valentina Giraldi, Giandomenico Magagnano, Daria Giacomini, Pier Giorgio Cozzi and Andrea Gualandi

Beilstein J. Org. Chem. **2024,** *20,* 2461–2468. doi:10.3762/bjoc.20.210

Reaction optimization studies, general experimental procedures, product isolation and characterization, spectroscopic data for new compounds, and copies of NMR spectra

License and Terms: This is a supporting information file under the terms of the Creative Commons Attribution License (https://creativecommons.org/licenses/by/4.0). Please note that the reuse, redistribution and reproduction in particular requires that the author(s) and source are credited and that individual graphics may be subject to special legal provisions.
The license is subject to the *Beilstein Journal of Organic Chemistry* terms and conditions: (https://www.beilstein-journals.org/bjoc/terms)

Table of contents

Experimental procedures and characterization	S2
General methods and materials	S2
General procedure A the for the synthesis of 4-alkoxyazetidinones	S5
Characterization of the of 4-alkoxyazetidinones	S6
General procedure B for the photocatalytic cyclization reaction	S11
Characterization of the target compounds **11c–h**, **12c–f**, and **14**	S12
NOE experiments for the determination of relative configuration	S18
Copies of ^{1}H and ^{13}C NMR spectra	S20

Experimental procedures and characterization

General methods and materials

^{1}H NMR spectra were recorded on Varian Mercury 400 and Bruker AVANCE III 600 MHz spectrometers. Chemical shifts are reported in ppm from TMS with the solvent resonance as the internal standard ($CHCl_3$: δ = 7.27 ppm). Data are reported as follows: chemical shift, multiplicity (s = singlet, d = duplet, t = triplet, q = quartet, dd = double duplet, m = multiplet), coupling constants (Hz).
^{13}C NMR spectra were recorded on Varian Mercury 400 and Bruker AVANCE III 600 MHz spectrometers. Chemical shifts are reported in ppm from TMS with the solvent as the internal standard ($CDCl_3$: δ = 77.16 ppm). Chromatographic purifications were done with 240–400 mesh silica gel. All reactions were set up under an argon atmosphere in oven-dried glassware using standard Schlenk techniques.
ATR-FTIR spectra of pure compounds were recorded with a Bruker Alpha instrument in transmittance mode with a 4 cm^{-1} resolution in the 4000–400 cm^{-1} range.
HPLC-MS analyses were performed with an Agilent Technologies 1260 Infinity II instrument, coupled with an Agilent Technologies Infinity Lab LC/MSD XT single-quadrupole mass spectrometer in full scan mode from *m*/*z* = 50 to 2600, in positive ion mode (ESI+). The HPLC is equipped with a Phenomenex Gemini® 3 µm C18 (100 × 3 mm) column; the following method was used: mobile phase = H_2O/ACN (gradient from 30% to 80% of ACN in 8 minutes, then isocratic for 15 minutes), flow = 0.4 mL/min, temperature = 40 °C.
HRMS were performed on Waters Xevo G2-XS QTof, ESI+, cone voltage 40 V, Capillary 3KV, source temperature 120 °C.
Anhydrous solvents were supplied by Aldrich in Sureseal® bottles. Unless specified, anhydrous solvents were used without further purifications. All the reagents were purchased from commercial sources (Sigma-Aldrich, Alfa Aesar, Fluorochem, Strem Chemicals, TCI) and used without further purification unless specified. Reaction mixture was irradiated with Kessil® PR160L@456 nm.[1]
Photocatalysts **I**, **IV**, lactams **6**, **9** and alcohols **7a–c** were commercially available. Alcohols **7d**,[2] **7e**,[3] **7f**,[4] **7g**,[5] **7h**,[6] and **13**[7] were prepared according to the literature procedures. 3CzClIPN was prepared according to literature procedure.[8]

1 https://kessil.com/products/science_PR160L.php
2 Meiß, R.; Kumar, K.; Waldmann, H. *Chem. – A Eur. J.* **2015**, *21*, 13526–13530.
3 Bober, A. E.; Proto, J. T.; Brummond, K. M. *Org. Lett.* **2017**, *19*, 1500–1503.
4 Nguyen, T. N. T.; Thiel, N. O.; Pape, F.; Teichert, J. F. *Org. Lett.* **2016**, *18*, 2455–2458.
5 Ji, E.; Meng, H.; Zheng, Y.; Ramadoss, V.; Wang, *Eur. J. Org. Chem.* **2019**, 7367–7371.
6 Fujiwara, K.; Goto, A.; Sato, D.; Kawai, H.; Suzuki, T. *Tetrahedron Lett.* **2005**, *46*, 3465–3468.
7 Gilbert, N.; Ricard, S.; Bergeron, J.; Lambolez, P.; Daoust, B. *Eur. J. Org. Chem.* **2020**, 2517–2529.
8 Speckmeier, E.; Fischer, T. G.; Zeitler, K. *J. Am. Chem. Soc.* **2018**, *140*, 15353–15365.

Figure S1. Emission profile of the Kessil® PR160L@456 nm used to irradiate the solutions (form Kessil® website: https://www.kessil.com/science/PR160L.php).

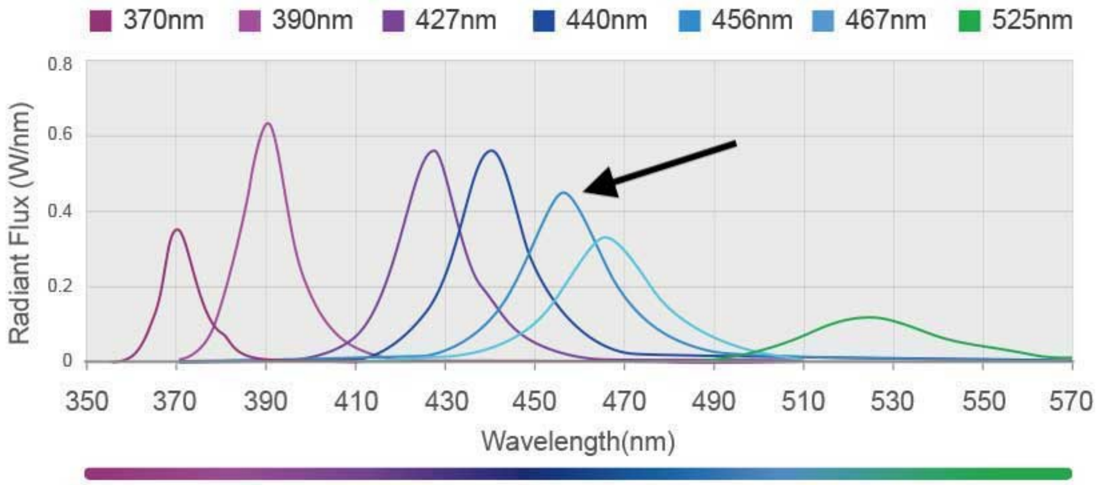

Figure S2. Reaction set-up. The images presented are sourced from the supplementary information of authors' published article,[9] which is published under a CC BY 4.0 license.

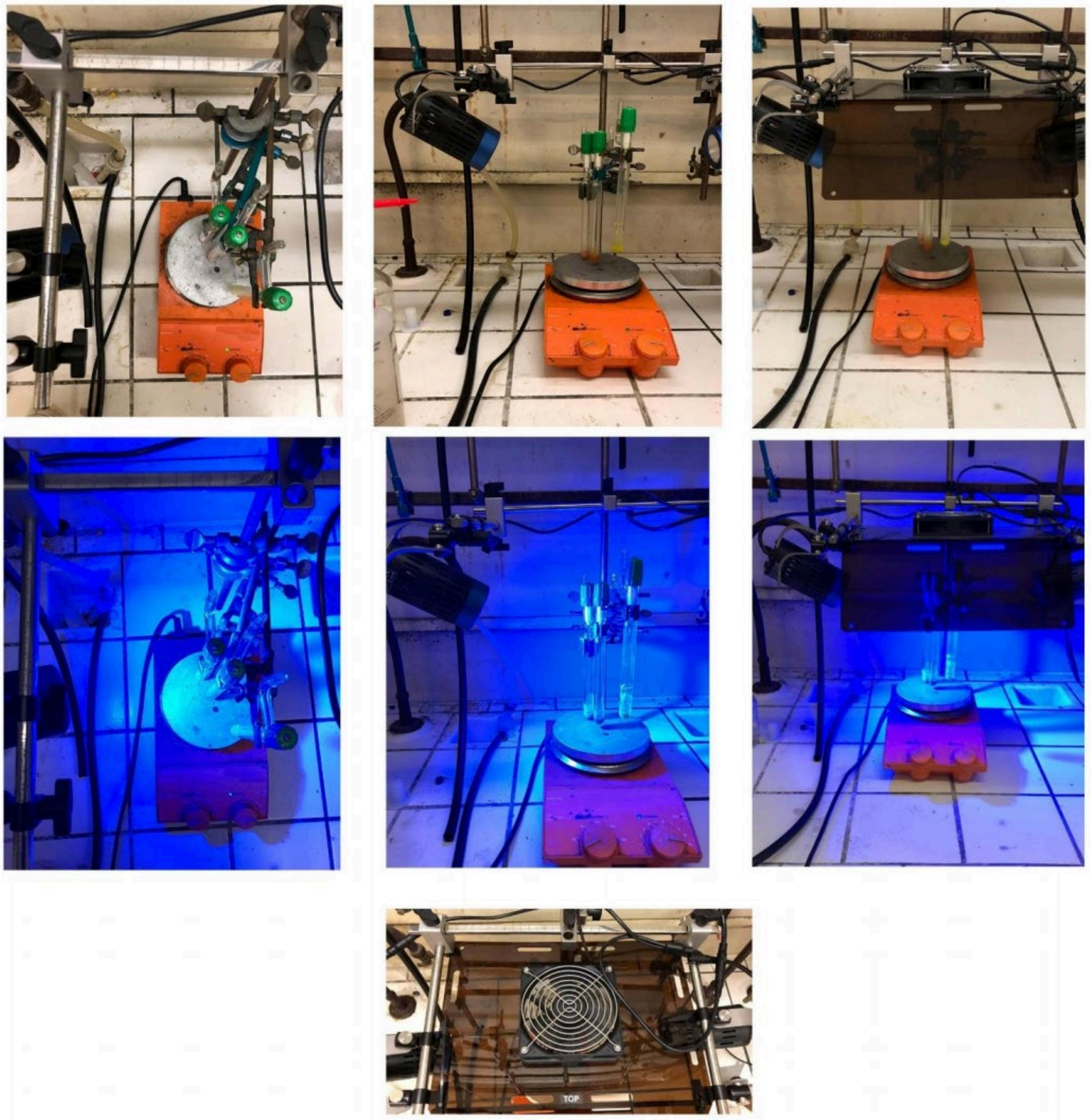

[9] Gualandi, A.; Calogero, F.; Mazzarini, M.; Guazzi, S.; Fermi, A.; Bergamini, G.; Cozzi, P. G. *ACS Catal.* **2020**, *10*, 3857–3863.

General procedure A for the synthesis of 4-alkoxyazetidinones

The 4-allyloxyazetidinones were synthetized using a modified procedure reported by Bachi and Hoornaert.[10] A flame-dried two-neck round bottom flask connected to a Schlenk line through a gas inlet under inert atmosphere, was charged with the 4-acetoxy-2-azetidinone (**6**), finely powdered $Zn(OAc)_2{\cdot}2H_2O$ (0.5 equiv), dry toluene (to obtain a 0.3 M solution of 4-acetoxy-2-azetidinone) and allyl alcohol **7a–h**. The reaction mixture was heated at 90 °C for 20 h under vigorous stirring and then quenched with water (approx. 15 mL) and extracted with EtOAc (4 × 10 mL). The combined organic layers were dried over anhydrous Na_2SO_4 and the solvent was removed under reduced pressure. The obtained residue was purified by flash column chromatography (SiO_2) to afford the (allyloxy)azetidinone products **8a–h** in the stated yields.

Scheme S1. Synthesis of compounds **8a–h** from **6**.

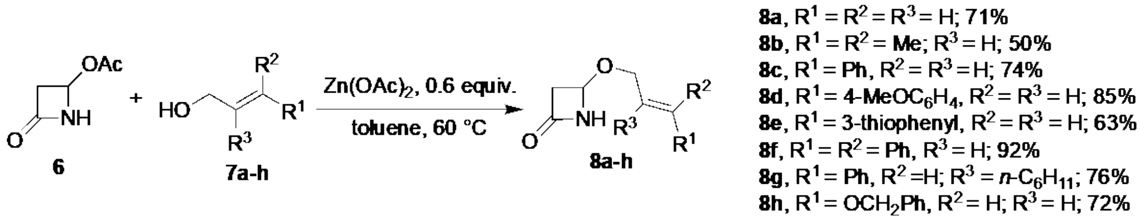

The **General Procedure A** was applied also to the (2*R*,3*R*)-3-((*R*)-1-((*tert*-butyldimethylsilyl)oxy)ethyl)-4-oxoazetidin-2-yl acetate (**9**) to afford **10c–f** products and to compound **13** to give compound **14**.

Scheme S2. A) Synthesis of compounds **10c-f** from **9**. B) Synthesis of compound **14**.

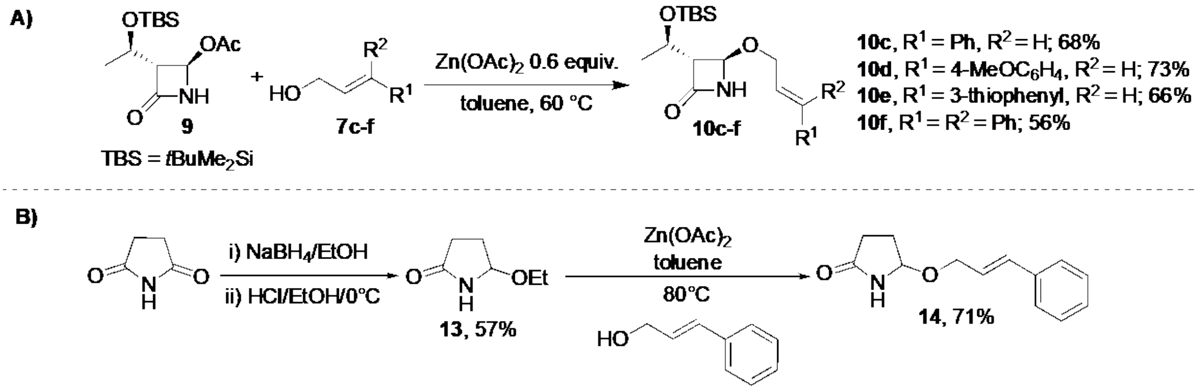

---

[10] Bachi M. D.; Frolow F.; Hoornaert, C. *J. Org. Chem.* **1983**, *48*, 1841-1849.

Characterization of the of 4-alkoxyazetidinones

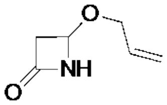

8a: yellowish oil, 71% (7.1 mmol, 900 mg). The **General Procedure A** was applied using **6** (10 mmol, 1.290 g) and **7a** (60 mmol, 3.5 g, 4.1 mL**)**. ^{1}H NMR (400 MHz, $CDCl_3$) δ 7.15 (bs, 1H), 5.95 – 5.82 (m, 1H), 5.29 (dq, *J* = 17.2, 1.5 Hz, 1H), 5.20 (dq, *J* = 10.4, 1.3 Hz, 1H), 5.06 (dd, *J* = 3.9, 1.4 Hz, 1H), 4.08 – 3.97 (m, 2H), 3.07 (ddd, *J* = 15.1, 3.9, 2.7 Hz, 1H), 2.86 (dd, *J* = 15.1, 1.5 Hz, 1H). ^{13}C NMR (100 MHz, $CDCl_3$) δ 167.1, 133.8, 118.2, 77.9, 69.3, 45.6. ATR-FTIR: *v* (cm^{-1}) 3263, 2928, 2870, 1743, 1459, 1411, 1377, 1344, 1273, 1185, 1163, 1124, 1075, 1031, 993, 926, 841, 818, 766, 647, 632. Spectroscopic data are in accordance with those reported in the literature.[10]

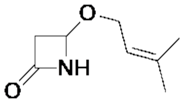

8b: yellowish oil, 50% (5.0 mmol, 775 mg). The **General Procedure A** was applied using **6** (10 mmol, 1.290 g) and **7b** (10 mmol, 861 mg, 1 mL**)**. ^{1}H NMR (400 MHz, $CDCl_3$) δ 6.69 (bs, 1H), 5.33 (ddq, *J* = 8.5, 5.8, 1.5 Hz, 1H), 5.07 (dd, *J* = 3.9, 1.5 Hz, 1H), 4.03 (qd, *J* = 11.4, 7.0 Hz, 2H), 3.09 (ddd, *J* = 15.1, 3.9, 2.8 Hz, 1H), 2.88 (dd, *J* = 15.1, 1.5 Hz, 1H), 1.76 (s, 3H), 1.69 (s, 3H). ^{13}C NMR (100 MHz, $CDCl_3$) δ 167.2, 138.7, 119.8, 77.7, 64.8, 45.6, 25.9, 18.2. ATR-FTIR: *v* (cm^{-1}) 3258, 2971, 2920, 1744, 1672, 1444, 1413, 1377, 1353, 1329, 1273, 1187, 1141, 1075, 1029, 995, 950, 925, 818, 766, 645, 615. HRMS *m/z* = $[M+H]^+$ calcd for $C_8H_{14}NO_2^+$ 156.1019, found 156.1022.

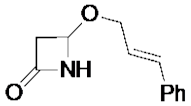

8c: white solid, 74% (7.4 mmol, 1.502 g). The **General Procedure A** was applied using **6** (10 mmol, 1.290 g) and **7c** (30 mmol, 4.025 g, 3.87 mL**)**. m. p. = 93-96 °C. ^{1}H NMR (400 MHz, $CDCl_3$) δ 7.42 – 7.36 (m, 2H), 7.36 – 7.30 (m, 2H), 7.26 (tt, *J* = 7.3, 1.5 Hz, 1H), 6.73 – 6.58 (m, 2H), 6.27 (dt, *J* = 15.9, 6.1 Hz, 1H), 5.15 (dd, *J* = 3.9, 1.5 Hz, 1H), 4.23 (dd, *J* = 6.1, 1.4 Hz, 2H), 3.13 (ddd, *J* = 15.1, 3.9, 2.8 Hz, 1H), 2.94 (dd, *J* = 15.1, 1.5 Hz, 1H). ^{13}C NMR (100 MHz, $CDCl_3$) δ 167.2, 136.2, 133.5, 128.7, 128.2, 126.6, 124.6, 77.8, 69.0, 45.6. ATR-FTIR: *v* (cm^{-1}) 3181, 3062, 3002, 2924, 2855, 1718, 1491, 1441, 1415, 1381, 1357, 1282, 1249, 1195, 1139, 1096, 1066, 1034, 1007, 979, 949, 923, 827, 808, 777, 740, 706, 688. Spectroscopic data are in accordance with those reported in the literature.[10]

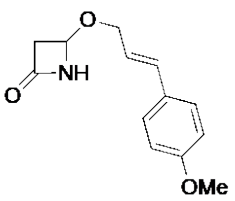

8d: white solid, 85% (2.1 mmol, 495 mg). The **General Procedure A** was applied using **6** (2.5 mmol, 325 mg) and **7d** (5 mmol, 821 mg**)**. m. p. = 105-107 °C. ^{1}H NMR (400 MHz, $CDCl_3$) δ 7.36 – 7.29 (m, 2H), 6.89 – 6.82 (m, 2H), 6.63 (bs, 1H), 6.58 (dd, *J* = 15.9, 1.5 Hz, 1H), 6.13 (dt, *J* = 15.9, 6.4 Hz, 1H), 5.14 (dd, *J* = 3.9, 1.5 Hz, 1H), 4.22 – 4.16 (m, 2H), 3.81 (s, 3H), 3.12 (ddd, *J* = 15.1, 3.9, 2.8 Hz, 1H), 2.93 (dd, *J* = 14.9, 1.5 Hz, 1H). ^{13}C NMR (100 MHz, $CDCl_3$) δ 167.2, 159.6, 133.3, 129.0, 127.9, 122.3, 114.1, 77.7, 69.2, 55.3, 45.6. ATR-FTIR: *v* (cm^{-1}) 3299, 2959, 2933, 2859, 1772, 1735, 1655, 1577, 1511, 1459, 1444, 1413, 1344, 1306, 1273, 1241, 1174, 1139, 1094, 1066, 1023, 991, 965, 837, 820, 781, 677, 632, 611. HRMS *m/z* = $[M+H]^+$ calcd for $C_{13}H_{16}NO_3^+$ 234.1125, found 234.1129.

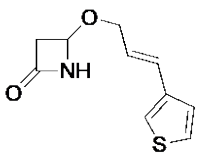

8e: white solid, 63% (2.8 mmol, 592 mg). The **General Procedure A** was applied using **6** (4.5 mmol, 581 mg) and **7e** (6.7 mmol, 939 mg). m. p. = 77-79 °C. ^{1}H NMR (400 MHz, $CDCl_3$) δ 7.26 (ddd, *J* = 5.0, 2.9, 0.6 Hz, 1H), 7.22 – 7.14 (m, 2H), 7.11 (bs, 1H), 6.62 (dt, *J* = 16.0, 1.4 Hz, 1H), 6.10 (dt, *J* = 15.8, 6.2 Hz, 1H), 5.11 (dd, *J* = 3.9, 1.5 Hz, 1H), 4.22 – 4.10 (m, 2H), 3.10 (ddd, *J* = 15.1, 3.9, 2.8 Hz, 1H), 2.89 (dd, *J* = 15.0, 1.4 Hz, 1H). ^{13}C NMR (100 MHz, $CDCl_3$) δ 167.3, 138.9, 127.7, 126.3, 125.0, 124.4, 123.0, 77.7, 68.9, 45.5. ATR-FTIR: *v* (cm^{-1}) 3304, 3103, 2939, 2859, 1774, 1735, 1340, 1278, 1241, 1178, 1139, 1092, 1064, 1029, 977, 960, 859, 822, 753, 738, 677, 634, 611. HRMS *m/z* = $[M+H]^+$ calcd for $C_{10}H_{12}NO_2S^+$ 210.0583, found 210.0587.

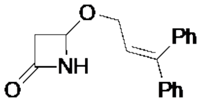

8f: white solid, 92% (0.46 mmol, 128 mg). The **General Procedure A** was applied using **6** (0.5 mmol, 65 mg) and **7f** (1 mmol, 210 mg). m. p. = 148-150 °C. ^{1}H NMR (400 MHz, $CDCl_3$) δ 7.43 – 7.35 (m, 3H), 7.32 – 7.23 (m, 5H), 7.20 – 7.13 (m, 2H), 6.20 (t, *J* = 6.9 Hz, 1H), 6.16 (bs, 1H), 5.04 (dd, *J* = 3.9, 1.5 Hz, 1H), 4.18 – 4.04 (m, 2H), 3.07 (ddd, *J* = 15.0, 3.9, 2.8 Hz, 1H), 2.87 (dd, *J* = 15.0, 1.5 Hz, 1H). ^{13}C NMR (100 MHz, $CDCl_3$) δ 166.7, 146.2, 141.3, 138.9, 129.7, 128.5, 128.4, 128.1, 128.0, 127.7, 123.5, 77.7, 65.9, 45.6. ATR-FTIR: *v* (cm^{-1}) 3308, 3103, 2939, 2861, 1776, 1735, 1571, 1320, 1282, 1243, 1178, 1146, 1087, 1068, 1031, 993, 962, 937, 859, 822, 755, 738, 690, 634, 611. HRMS *m/z* = $[M+H]^+$ calcd for $C_{18}H_{18}NO_2^+$ 280.1332, found 280.1335.

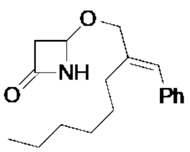

8g: orange sticky solid, 76% (1.9 mmol, 545 mg). The **General Procedure A** was applied using **6** (2.5 mmol, 323 mg) and **7g** (5 mmol, 1.09 g). ^{1}H NMR (400 MHz, $CDCl_3$) δ 7.38 – 7.30 (m, 2H), 7.24 (dt, *J* = 6.4, 1.1 Hz, 3H), 6.52 (s, 1H), 6.38 (bs, 1H), 5.16 (dd, *J* = 4.0, 1.5 Hz, 1H), 4.20 – 4.07 (m, 2H), 3.15 (ddd, *J* = 15.0, 3.9, 2.8 Hz, 1H), 2.97 (dd, *J* = 15.1, 1.5 Hz, 1H), 2.33 – 2.26 (m, 2H), 1.56 – 1.42 (m, 2H), 1.37 – 1.18 (m, 6H), 0.98 – 0.78 (m, 3H). ^{13}C NMR (100 MHz, $CDCl_3$) δ 167.2, 138.8, 137.1, 128.7, 128.4, 128.3, 126.9, 126.5, 78.0, 72.7, 45.6, 31.7, 29.5, 28.9, 28.2, 22.7, 14.2. ATR-FTIR: ν (cm^{-1}) 3278, 3058, 3025, 2954, 2924, 2857, 1754, 1675, 1493, 1459, 1411, 1377, 1344, 1269, 1183, 1135, 1077, 1031, 954, 919, 850, 747, 699, 647. HRMS *m/z* = $[M+H]^+$ calcd for $C_{18}H_{26}NO_2^+$ 288.1958, found 288.1962.

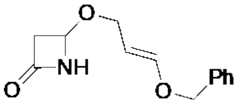

8h: colorless oil, 72% (1.8 mmol, 419 mg). The **General Procedure A** was applied using **6** (2.5 mmol, 323 mg) and **7h** (3.75 mmol, 240 mg). ^{1}H NMR (400 MHz, $CDCl_3$) δ 7.46 – 7.28 (m, 5H), 6.71 (bs, 1H), 6.61 (d, *J* = 12.6 Hz, 1H), 5.07 – 4.94 (m, 2H), 4.79 (s, 2H), 4.02 – 3.92 (m, 2H), 3.07 (ddd, *J* = 15.0, 4.0, 2.8 Hz, 1H), 2.85 (dd, *J* = 15.1, 1.5 Hz, 1H). ^{13}C NMR (100 MHz, $CDCl_3$) δ 167.0, 151.0, 136.5, 128.8, 128.4, 127.7, 100.1, 77.4, 71.5, 66.5, 45.8. ATR-FTIR: ν (cm^{-1}) 3263, 2969, 2933, 2872, 1752, 1649, 1498, 1456, 1411, 1379, 1353, 1288, 1182, 1133, 1072, 1027, 993, 924, 816, 801, 740, 697, 663, 634, 613. HRMS *m/z* = $[M+H]^+$ calcd for $C_{18}H_{26}NO_2^+$ 234.1125, found 234.1127.

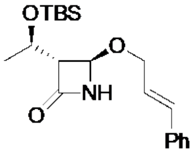

10c: white solid, 68% (1.7 mmol, 613 mg). The **General Procedure A** was applied using **9** (2.5 mmol, 720 mg) and **7c** (7.5 mmol, 1.0 g). m. p. = 87-89 °C. ^{1}H NMR (400 MHz, $CDCl_3$) δ 7.38 (dt, *J* = 6.4, 1.4 Hz, 2H), 7.33 (ddd, *J* = 7.6, 6.7, 1.4 Hz, 2H), 7.30 – 7.23 (m, 2H), 6.68 – 6.60 (m, 1H), 6.37 – 6.22 (m, 2H), 5.16 (d, *J* = 1.4 Hz, 1H), 4.30 – 4.14 (m, 3H), 3.09 (dd, *J* = 4.0, 1.3 Hz, 1H), 1.28 (d, *J* = 6.4 Hz, 3H), 0.87 (s, 9H), 0.08 (s, 3H), 0.07 (s, 3H). ^{13}C NMR (100 MHz, $CDCl_3$) δ 168.0, 136.4, 133.2, 128.7, 128.1, 126.6, 125.0, 80.5, 69.1, 65.9, 64.4, 25.8, 22.7, 18.0, -4.2, -4.9. ATR-FTIR: *ν* (cm^{-1}) 3029, 2928, 2858, 1770, 1667, 1603, 1583, 1496, 1454, 1397, 1377, 1335, 1286, 1192, 1171, 1119, 1029, 956, 913, 836, 747, 700, 630, 558; HRMS *m/z* = $[M+Na]^+$ calcd for $C_{20}H_{31}NNaO_3Si^+$ 384.1971, found 384.1944.

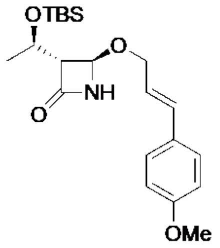

10d: white solid, 73% (0.51 mmol, 199 mg). The **General Procedure A** was applied using **9** (0.7 mmol, +202 mg) and **7d** (2.1 mmol, 345 mg). m. p. = 98-101 °C. ^{1}H NMR (400 MHz, $CDCl_3$) δ 7.35 – 7.29 (m, 2H), 6.90 – 6.82 (m, 2H), 6.59 (d, *J* = 15.9 Hz, 1H), 6.27 (bs, 1H), 6.14 (dt, *J* = 15.9, 6.3 Hz, 1H), 5.15 (d, *J* = 1.3 Hz, 1H), 4.30 – 4.06 (m, 3H), 3.81 (s, 3H), 3.09 (dd, *J* = 4.0, 1.3 Hz, 1H), 1.27 (d, *J* = 6.3 Hz, 3H), 0.87 (s, 9H), 0.08 (s, 3H), 0.06 (s, 3H). ^{13}C NMR (100 MHz, $CDCl_3$) δ 167.8, 159.7, 133.1, 129.1, 127.9, 122.7, 114.2, 80.5, 69.4, 66.0, 64.4, 55.4, 25.9, 22.7, 18.1, -4.2, -4.9. ATR-FTIR: ν (cm^{-1}) 3222, 2954, 2928, 2857, 1769, 1739, 1687, 1605, 1510, 1461, 1439, 1370, 1359, 1336, 1292, 1247, 1172, 1131, 1094, 1062, 1034, 1010, 969, 937, 880, 826, 807, 779, 757, 718, 693, 669, 626. HRMS *m/z* = $[M+Na]^+$ calcd for $C_{21}H_{33}NNaO_4Si^+$ 414.2077, found 414.2073.

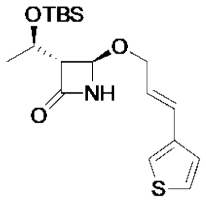

10e: orange sticky solid, 66% (2.97 mmol, 1.089 g). The **General Procedure A** was applied using **9** (4.5 mmol, 1.293 g) and **7e** (6.7 mmol, 939 mg) ^{1}H NMR (400 MHz, $CDCl_3$) δ 7.34 – 7.29 (m, 1H), 6.65 (bs, 1H), 6.43 (dt, *J* = 15.8, 1.6 Hz, 1H), 6.34 (dd, *J* = 3.3, 1.8 Hz, 1H), 6.23 (d, *J* = 3.3 Hz, 1H), 6.17 (dt, *J* = 15.8, 5.9 Hz, 1H), 5.10 (d, *J* = 1.3 Hz, 1H), 4.22 – 4.11 (m, 3H), 3.04 (dd, *J* = 4.2, 1.3 Hz, 1H), 1.23 (d, *J* = 2.0 Hz, 3H), 0.84 (s, 9H), 0.05 (s, 3H), 0.03 (s, 3H). ^{13}C NMR (100 MHz, $CDCl_3$) δ 168.2, 139.0, 127.4, 126.3, 125.0, 124.7, 122.9, 80.5, 69.0, 65.8, 64.4, 25.8, 22.6, 18.0, -4.2, -4.9. ATR-FTIR: *ν* (cm^{-1}) 3280, 2954, 2930, 2887, 2857, 1754, 1687, 1463, 1375, 1362, 1342, 1251, 1169, 1144, 1079, 1031, 1005, 967, 937, 876, 829, 773, 663, 613. HRMS *m/z* = $[M+Na]^+$ calcd for $C_{18}H_{29}NNaO_3SSi^+$ 390.1535, found 390.1533.

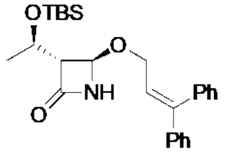

10f: colorless oil, 56% (0.28 mmol, 122 mg). The **General Procedure A** was applied using **9** (0.5 mmol, 143 mg) and **7g** (2.5 mmol, 525 mg). ^{1}H NMR (400 MHz, $CDCl_3$) δ 7.42 – 7.33 (m, 4H), 7.31 – 7.23 (m, 6H), 7.17 (dt, *J* = 7.4, 1.3 Hz, 2H), 6.20 (t, *J* = 6.9 Hz, 1H), 6.12 (bs, 1H), 5.05 (d, *J* = 1.3 Hz, 1H), 4.15 (tt, *J* = 6.4, 3.2 Hz, 1H), 4.12-4.08 (m, 2H), 3.00 (dd, *J* = 3.7, 1.3 Hz, 1H), 1.20 (d, *J* = 6.3 Hz, 3H), 0.85 (s, 9H), 0.05 (s, 3H), 0.03 (s, 3H). ^{13}C NMR (151 MHz, $CDCl_3$) δ 167.7, 146.2, 141.4, 139.0, 129.8, 128.5, 128.5, 128.4, 128.0, 128.0, 127.7, 123.8, 80.1, 66.0, 65.8, 64.3, 25.9, 22.6, 18.1, -4.2, -5.0. ATR-FTIR: ν (cm^{-1}) 3084, 3062, 3028, 2952, 2928, 2885, 1765, 1722, 1664, 1599, 1493, 1463, 1444, 1401, 1370, 1359, 1338, 1308, 1280, 12552,

1198, 1176, 1139, 1121, 1096, 1068, 1008, 975, 958, 937, 889, 827, 807, 773, 755, 729, 696, 632. HRMS *m/z* = $[M+Na]^+$ calcd for $C_{26}H_{35}NNaO_3Si^+$ 460.2284, found 460.2280.

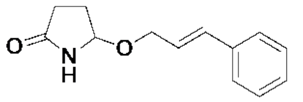

14: yellow sticky solid, 71% (240 mg, 1.11 mmol). The **General Procedure A** was applied using **13** (1.55 mmol, 200 mg, 1 eq) and **7c** (2.33 mmol, 312 mg, 2.3 eq). The crude was purified by flash chromatography on silica gel (40:60 to 70:30 EtOAc/n-Hex). ^{1}H NMR (400 MHz, $CDCl_3$) δ 8.10 (bs, 1H), 7.40 – 7.14 (m, 5H), 6.61 (dt, J = 15.9, 1.6 Hz, 1H), 6.23 (dt, J = 15.9, 6.1 Hz, 1H), 5.04 (dt, J = 6.0, 1.4 Hz, 1H), 4.20 (ddd, J = 12.4, 5.9, 1.5 Hz, 1H), 4.08 (ddd, J = 12.5, 6.3, 1.5 Hz, 1H), 2.59 – 2.42 (m, 1H), 2.35 – 2.15 (m, 2H), 2.15 – 2.03 (m, 1H). ^{13}C NMR (100 MHz, $CDCl_3$) δ 179.8, 136.5, 133.1, 128.7, 127.9, 126.6, 125.2, 85.6, 68.2, 28.5, 28.4. Spectroscopic data are in accordance with those reported in the literature.[11]

[11] Dascalu, A.-E.; Ghinet, A.; Lipka, E.; Collinet, M.; Rigo, B.; Billamboz, M. *Mol. Catal.* **2019**, *470*, 32–39.

General procedure B for the photocatalytic cyclization reaction.

Scheme S3. Photocatalytic cyclization reactions.

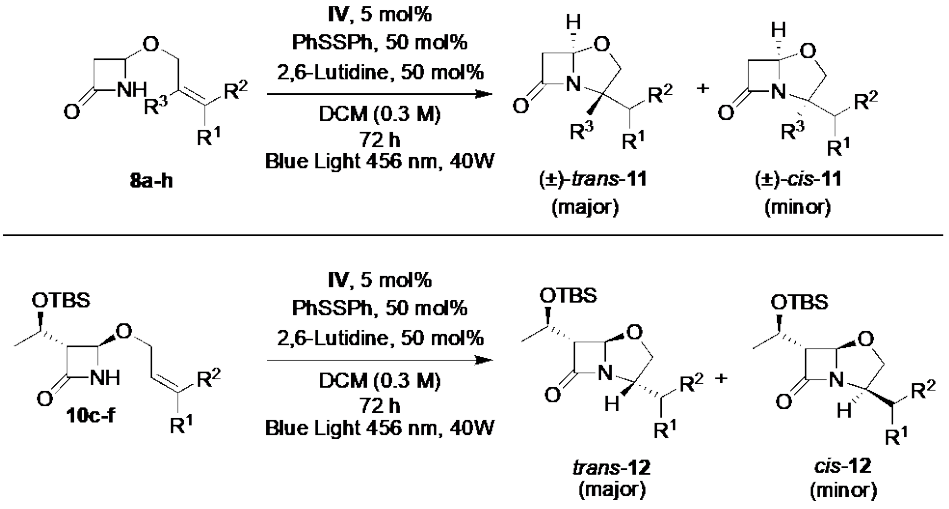

All the reactions were performed on 0.2 mmol scale of (allyloxy)azetidinones **8a–h** or **10c–f**. A dry 10 mL Schlenk tube, equipped with a Rotaflo® stopcock, magnetic stirring bar and an argon supply tube, was charged under argon with the 9-mesityl-3,6-di-*tert*-butyl-10-phenylacridinium tetrafluoroborate organic photocatalyst (Fukuzumi catalyst (**IV**), 4 mol %, 0.01 mmol, 4.5 mg), the substrate (0.2 mmol) and the phenyl disulfide (50 mol %, 0.1 mmol, 0.5 equiv, 21.8 mg). Dry DCM (700 µL in order to obtain a 0.3 M substrate solution) was then added and the yellow reaction mixture was allowed to stir for 5 minutes. The reaction mixture was further subjected to a freeze-pump-thaw procedure (three cycles, five minutes each) and the vessel was refilled with argon. The reaction vessel then was briefly open under argon and degassed 2,6-lutidine (50 mol %, 0.5 equiv, 0.1 mmol, 12 µL) were added. The reaction mixture was irradiated under vigorous stirring for 72 h. After that, the reaction mixture was quenched with water (approx. 4 mL) and extracted with DCM (4 × 3 mL). The combined organic layers were dried over anhydrous Na_2SO_4 and the solvent was removed under reduced pressure.

The ^{1}H NMR of the reaction crude was recorded in order to evaluate the diastereomeric ratio. The crude was purified by flash column chromatography (SiO_2) to afford products **11** and **12** in the stated yields.

Characterization of the target compounds 11c-h, 12c–f, and 14

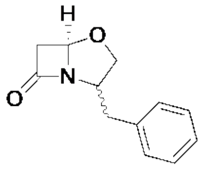

(±)-**11c**, pale yellow oil, 70% (0.14 mmol, 28 mg) as mixture of diastereomers (±)-*trans*-**11c**:(±)-*cis*-**11c** of 1.4:1. The **General Procedure B** was applied using **8c** (0.2 mmol, 40.6 mg). The diastereomeric ratio was determined by ^{1}H NMR analysis of the reaction mixture, integrating the signals at 5.21 ppm *cis* (1H) and 5.17 ppm *trans* (1H). The title compound was isolated by flash column chromatography (8:2 Hex:AcOEt). The overall yield (70%) refers to the sum of the two diastereoisomers (±)-*trans*-**11c** and (±)-*cis*-**11c**. ^{1}H NMR (400 MHz, $CDCl_3$) δ 7.36 – 7.19 (m, 5H_{trans}, 5H_{cis}), 5.21 (d, *J* = 2.8 Hz, 1H_{cis}), 5.17 (d, *J* = 2.7 Hz, 1H_{trans}), 4.35 – 4.28 (m, 1H_{trans}), 4.19 (dd, *J* = 6.6, 8.7 Hz, 1H_{trans}), 4.08 (dd, *J* = 5.9, 8.3 Hz, 1H_{cis}), 3.87 – 3.77 (m, 1H_{trans}, 1H_{cis}), 3.71 – 3.59 (m, 2H_{cis}), 3.27 – 3.18 (m, 1H_{trans}, 1H_{cis}), 2.94 – 2.77 (m, 2H_{trans}, 2H_{cis}), 2.71 (dd, *J* = 7.4, 14.1 Hz, 1H_{trans}). ^{13}C NMR (100 MHz, $CDCl_3$) δ 177.9 ($C6_{trans}$), 175.7 ($C6_{cis}$), 138.2 ($C7_{cis}$), 137.2 ($C7_{trans}$), 129.2 (2$C8_{trans}$), 128.9 (2$C8_{cis}$), 128.8 (2$C9_{cis}$), 128.8 (2$C9_{trans}$), 126.9 (2$C10_{trans+cis}$), 85.0 ($C1_{cis}$), 84.4 ($C1_{trans}$), 75.0 ($C2_{trans}$), 74.8 ($C2_{cis}$), 63.1 ($C3_{cis}$), 59.3 ($C3_{trans}$), 45.2 ($C5_{cis}$), 44.3 ($C5_{trans}$), 38.6 ($C4_{trans}$), 34.5 ($C4_{cis}$); HRMS *m/z* = $[M+H]^+$ calcd for $C_{12}H_{14}NO_2^+$ 204.1019, found 204.1022.

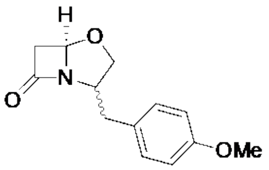

(±)-**11d:** pale yellow oil, 68% (0.14 mmol, 32 mg) as mixture diastereomers (±)-*trans*-**11d**:(±)-*cis*-**11d** of 6.7:1. The **General Procedure B** was applied using **8d** (0.2 mmol, 47 mg). The diastereomeric ratio was determined by ^{1}H NMR analysis of the reaction mixture, integrating the signals at 5.20 ppm *trans* (1H) and 5.15 ppm *cis* (1H). The title compound was isolated by flash column chromatography (8:2- Hex:AcOEt). The overall yield (68%) refers to the sum of the two diastereoisomers (±)-*trans*-**11d** and (±)-*cis*-**11d**. ^{1}H NMR (400 MHz, $CDCl_3$) δ 7.26 – 7.18 (m, 2H_{trans}), 7.14 (d, *J* = 8.4 Hz, 2H_{cis}), 6.88 – 6.82 (m, 2H_{trans}+2H_{cis}), 5.20 (dd, *J* = 2.9, 0.9 Hz, 1H_{trans}), 5.15 (d, J=2.7 1H_{cis}), 4.31 – 4.21 (m, 1H_{cis}), 4.18 (dd, *J* = 8.6, 6.5 Hz, 1H_{cis}), 4.07 (dd, *J* = 8.4, 5.6 Hz, 1H_{trans}), 3.87 – 3.81 (m, 1H_{cis}), 3.81 – 3.75 (m, 4H_{trans}+4H_{cis}), 3.65 – 3.53 (m, 2H_{trans}), 3.27 – 3.15 (m, 1H_{trans}+1H_{cis}), 2.89 (d, *J* = 16.3 Hz, 1H_{trans}), 2.88 – 2.75 (m, 2H_{trans}+2H_{cis}), 2.65 (dd, *J* = 14.1, 7.3 Hz, 1H_{cis}). ^{13}C NMR (100 MHz, $CDCl_3$) 177.8, 175.6 158.4, 130.1, 129.7, 129.1, 114.0, 84.9, 84.2, 76.8, 74.7, 63.2, 59.4, 55.2, 45.0, 44.2, 37.5, 33.5. ATR-FTIR: ν (cm^{-1}) 2953, 2909, 2836, 1767, 1645, 1611, 1583, 1511, 1463, 1440, 1419, 1351, 1328, 1300, 1287, 1244, 1175, 1127, 1114, 1029, 985, 946, 831, 776, 749, 725, 704, 669, 638, 609, 544, 518, 487. HRMS *m/z* = $[M+H]^+$ calcd for $C_{13}H_{16}NO_3^+$ 234.1125, found 234.1129.

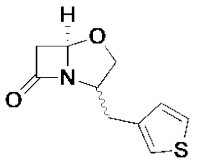

(±)-**11e**: pale yellow oil, 70% (0.14 mmol, 29 mg) as mixture diastereomers (±)-*trans*-**11e**:(±)-*cis*-**11e** of 2.7:1. The **General Procedure B** was applied using **8e** (0.2 mmol, 42 mg). The diastereomeric ratio was determined by ^{1}H NMR analysis of the reaction mixture, integrating the signals at 5.21 ppm *trans* (1H) and 5.15 ppm *cis* (1H). The title compound was isolated by flash column chromatography (9:1 Hex:AcOEt). The overall yield (70%) refers to the sum of the two diastereoisomers (±)-*trans*-**11e** and (±)-*cis*-**11e**. ^{1}H NMR (400 MHz, $CDCl_3$) δ 7.34 – 7.23 (m, $1H_{trans}$ + $1H_{cis}$), 7.19 – 7.10 (m, $1H_{trans}$ + $1H_{ci}$), 7.03 (dd, J = 4.9, 1.4 Hz, $1H_{trans}$), 6.99 (dd, J = 4.9, 1.4 Hz, $1H_{cis}$), 5.21 (d, J = 2.8 Hz, $1H_{trans}$), 5.15 (d, J = 2.7 Hz, $1H_{cis}$), 4.35 – 4.22 (m, $2H_{cis}$), 4.17 – 4.07 (m, $1H_{trans}$), 3.79 (ddd, J = 8.6, 5.2, 2.6 Hz, $1H_{trans}$ + $1H_{cis}$), 3.69 – 3.56 (m, $2H_{trans}$), 3.22 (ddd, J = 16.4, 7.7, 2.8 Hz, $1H_{trans}$ + $1H_{cis}$), 3.03-2.73 (m, $2H_{trans}$ + $4H_{cis}$). ^{13}C NMR (100 MHz, $CDCl_3$) δ 177.9, 175.6, 138.4, 137.5, 135.9, 128.4, 128.2, 126.1, 126.0, 122.1, 85.1, 84.4, 75.3, 75.1, 62.5, 58.8, 45.2, 44.3, 33.2, 29.3. HRMS *m/z* = $[M+H]^+$ calcd for $C_{18}H_{18}NO_2S^+$ 210.0583, found 210.0586.

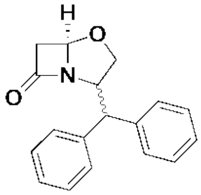

(±)-**11f**: pale yellow oil, 57% (0.11 mmol, 32 mg) as mixture diastereomers (±)-*trans*-**11f**:(±)-*cis*-**11f** of 1.7:1. The **General Procedure B** was applied using **8f** (0.2 mmol, 56 mg). The diastereomeric ratio was determined by ^{1}H NMR analysis of the reaction mixture, integrating the signals at 5.34 ppm *trans* (1H) and 5.12 ppm *cis* (1H). The title compound was isolated by flash column chromatography (9:1 Hex:AcOEt). The overall yield (57%) refers to the sum of the two diastereoisomers (±)-*trans*-**11f** and (±)-*cis*-**11f**. ^{1}H NMR (400 MHz, $CDCl_3$) δ 7.54 – 7.47 (m, $2H_{trans}$), 7.40 (dt, J = 6.9, 1.2 Hz, $2H_{cis}$), 7.37 – 7.17 (m, $8H_{trans}$+$8H_{cis}$), 5.34 (d, J = 2.9 Hz, $1H_{trans}$), 5.12 (dd, J = 2.8, 0.7 Hz, $1H_{cis}$), 4.82 (ddd, J = 10.0, 6.7, 5.7 Hz, $1H_{cis}$), 4.72 (d, J = 11.7 Hz, $1H_{trans}$), 4.26 – 4.15 (m, $1H_{trans}$+ $1H_{cis}$), 3.94 – 3.81 (m, $1H_{trans}$+ $2H_{cis}$), 3.64 (dd, J = 9.5, 8.4 Hz, $1H_{trans}$), 3.25 – 3.15 (m, $1H_{trans}$+ $1H_{cis}$), 2.88 – 2.80 (m, $1H_{trans}$+ $1H_{cis}$). ^{13}C NMR (100 MHz, $CDCl_3$) δ 177.8, 175.9, 142.4, 141.8, 141.7, 141.4, 129.1, 129.0, 128.7, 128.6, 128.4, 128.3, 127.8, 127.8, 127.4, 127.2, 126.9, 126.9, 85.6, 84.8, 75.3, 73.9, 65.5, 61.8, 53.6, 49.7, 46.0, 44.4. ATR-FTIR: ν (cm^{-1}) 3027, 2948, 2866, 1771, 1636, 1597, 1583, 1495, 1476, 1451, 1395, 1350, 1332, 1275, 1179, 1130, 1045, 1019, 1003, 982, 946, 911, 837, 763, 743, 696, 657, 618, 598, 554, 503, 489, 475. HRMS *m/z* = $[M+H]^+$ calcd for $C_{18}H_{18}NO_2^+$ 280.1332, found 280.1336.

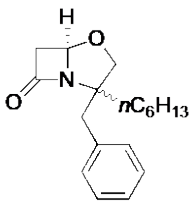

(±)-**11g**: yellowish oil, 47% (0.09 mmol, 27 mg) as mixture diastereomers (±)-*trans*-**11g**:(±)-*cis*-**11g** of 1.4:1. The **General Procedure B** was applied using **8g** (0.2 mmol, 57 mg). The diastereomeric ratio was determined by ^{1}H NMR analysis of the reaction mixture, integrating the signals at 5.24 ppm *cis* (1H) and 4.95 ppm *trans* (1H). The title compound was isolated by flash column chromatography (9:1 Hex:AcOEt). The overall yield (47%) refers to the sum of the two diastereoisomers (±)-*trans*-**11g** and (±)-*cis*-**11g**. ^{1}H NMR (600 MHz, CDCl3) δ 7.31 – 7.20 (m, $5H_{trans}$+ $5H_{c}$), 5.24 (dd, *J* = 2.9, 0.9 Hz, $1H_{cis}$), 4.95 (dd, *J* = 2.8, 0.9 Hz, $1H_{trans}$), 4.10 – 4.01 (m, $1H_{trans}$+ $1H_{cis}$), 3.86 (d, *J* = 8.4 Hz, $1H_{trans}$), 3.75 (d, *J* = 8.3 Hz, $1H_{cis}$), 3.57 (d, *J* = 14.1 Hz, $1H_{cis}$), 3.19 (dd, *J* = 16.2, 2.9 Hz, $1H_{cis}$), 3.12 – 3.06 (m, $1H_{trans}$+ $1H_{cis}$), 2.91 (dd, *J* = 16.2, 0.9 Hz, $1H_{cis}$), 2.81 (dd, *J* = 16.2, 0.9 Hz, $1H_{trans}$), 2.78 – 2.71 (m, $2H_{trans}$), 1.93 (ddd, *J* = 13.9, 11.6, 4.3 Hz, 1H), 1.80 (ddd, *J* = 14.0, 12.3, 3.7 Hz, 1H), 1.66 – 1.59 (m, 1H), 1.52 – 1.38 (m, 2H), 1.37 – 1.21 (m, 17H), 0.91 – 0.85 (m, $3H_{trans}$+ $3H_{cis}$). ^{13}C NMR (151 MHz, $CDCl_3$) δ 175.4, 174.5, 136.7, 136.5, 130.5, 130.0, 128.6, 128.3, 126.9, 126.9, 84.4, 84.4, 79.4, 79.1, 70.3, 70.2, 44.7, 44.5, 40.7, 38.1, 33.8, 33.1, 31.8, 31.7, 29.7, 29.5, 25.2, 24.2, 22.7, 22.7, 14.2. HRMS *m/z* = $[M+H]^+$ calcd for $C_{18}H_{26}NO_2^+$ 288.1958, found 288.1961.

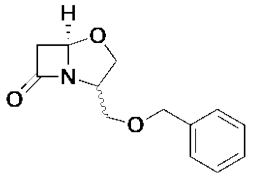

(±)-**11h:** pale yellow oil, 41% (0.08 mmol, 19 mg) as mixture diastereomers (±)-*trans*-**11h**:(±)-*cis*-**11h** of 1.1:1. The **General Procedure B** was applied using **8h** (0.2 mmol, 47 mg). The diastereomeric ratio was determined by ^{1}H NMR analysis of the reaction mixture, integrating the signals at 5.20 ppm *trans* (1H) and 5.13 ppm *cis*. The title compound was isolated by flash column chromatography (9:1 Hex:AcOEt). The overall yield (41%) refers to the sum of the two diastereoisomers (±)-*trans*-**11h** and (±)-*cis*-**11h**. ^{1}H NMR (400 MHz, $CDCl_3$) δ 7.39 – 7.28 (m, 10H), 5.20 (dd, *J* = 2.7, 0.8 Hz, $1H_{trans}$), 5.13 (dd, *J* = 2.8, 0.9 Hz, $1H_{cis}$), 4.70 (s, 1H), 4.61 – 4.50 (m, 5H), 4.29 (dd, *J* = 8.4, 6.9 Hz, 1H), 4.25 – 4.19 (m, 1H), 4.17 (dd, *J* = 8.3, 7.0 Hz, 1H), 4.14 – 4.06 (m, 2H), 3.98 (dd, *J* = 8.4, 5.3 Hz, 1H), 3.84 – 3.74 (m, 2H), 3.71 – 3.60 (m, 1H), 3.51 (dd, *J* = 9.7, 4.8 Hz, 1H), 3.43 (dd, *J* = 9.7, 6.3 Hz, 1H), 3.25 (dd, *J* = 16.1, 2.7 Hz, 1H), 3.15 (ddd, *J* = 16.2, 2.8, 1.1 Hz, 1H), 2.92 – 2.86 (m, 1H), 2.86 – 2.81 (m, 1H). ^{13}C NMR (100 MHz, $CDCl_3$) δ 177.9, 175.0, 137.9, 137.8, 128.7, 128.6, 128.6, 128.0, 127.9, 127.9, 127.8, 127.1, 84.8, 84.7, 74.7, 73.6, 73.5, 73.5, 69.8, 67.4, 60.6, 57.9, 44.6, 43.9. ATR-FTIR: *v* (cm^{-1}) 3029, 2960, 2925, 2855, 1770, 1736, 1489, 1453, 1417, 1363, 1260, 1177, 1086, 1048, 1025, 973, 914, 860, 799, 735, 697, 601, 459. HRMS *m/z* = $[M+H]^+$ calcd for $C_{13}H_{16}NO_3^+$ 234.1125, found 234.1128.

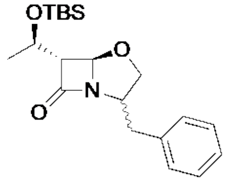

12c: the **General Procedure B** was applied using **10c** (0.2 mmol, 72 mg). The diastereomeric ratio (1.2:1) was determined by ^{1}H NMR analysis of the reaction mixture, integrating the signals at 5.20 ppm C*is* (1H) and 5.18 ppm *Trans* (1H). The *trans* diastereomer (*trans*-**12c**, 29 mg) and the minor diastereomer (*cis*-**12c**, 18 mg) were isolated by flash chromatography (9:1 Hex:AcOEt) as colourless oils, giving an overall product yield of 65% (0.13 mmol, 47 mg). *trans*-**12c**: ^{1}H NMR (400 MHz, $CDCl_3$) 7.37 – 7.14 (m, 5H), 5.18 (s, 1H), 4.29 (qd, *J* = 6.8, 4.6 Hz, 1H), 4.17 (qd, *J* = 6.3, 4.2 Hz, 1H), 4.11 (dd, *J* = 8.6, 6.4 Hz, 1H), 3.84 (dd, *J* = 8.6, 4.7 Hz, 1H), 2.95 (dd, *J* = 4.3, 0.8 Hz, 1H), 2.86 (dd, *J* = 14.0, 6.8 Hz, 1H), 2.69 (dd, *J* = 14.0, 7.3 Hz, 1H), 1.25 (d, *J* = 6.3 Hz, 4H), 0.89 (s, 9H), 0.06 (s, 3H), 0.05 (s, 3H). ^{13}C NMR (100 MHz, $CDCl_3$) δ 178.1, 137.2, 129.2, 128.5, 126.7, 86.4, 74.2, 65.4, 64.1, 58.8, 38.4, 25.7, 22,4, 17.9, -4.5, -5.1. ATR-FTIR: *ν* (cm^{-1}) 3029, 2954, 2929, 2884, 2856, 1778, 1661, 1604, 1497, 1472, 1462, 1455, 1375, 1361, 1330, 1287, 1252, 1171, 1137, 1105, 1074, 1038, 991, 940, 900, 830, 810, 775, 742, 699, 665, 576, 536, 494, 452. HRMS *m/z* = $[M+Na]^+$ calcd for $C_{20}H_{31}NNaO_3Si^+$ 384.1971, found 384.1967. *cis*-**12c**: ^{1}H NMR (400 MHz, $CDCl_3$) δ 7.34 – 7.21 (m, 5H), 5.20 (d, *J* = 0.9 Hz, 1H), 4.18 (qd, *J* = 6.3, 4.6 Hz, 1H), 4.05 (dd, *J* = 8.3, 5.8 Hz, 1H), 3.76 – 3.63 (m, 2H), 3.59 (tt, *J* = 7.4, 6.0 Hz, 1H), 3.02 (dd, *J* = 4.6, 0.9 Hz, 1H), 2.85 (dd, *J* = 13.7, 8.1 Hz, 1H), 1.26 (d, *J* = 6.2 Hz, 5H), 0.88 (s, 9H), 0.07 (d, *J* = 0.6 Hz, 6H). ^{13}C NMR (100 MHz, $CDCl_3$) δ 176.2, 138.3, 128.9, 128.8, 126.8, 87.4, 74.8, 66.1, 64.4, 62.5, 34.5, 25.8, 22.5, 18.1, -4.2, -4.9. ATR-FTIR: *ν* (cm^{-1}) 3028, 2954, 2928, 2884, 2856, 1771, 1644, 1636, 1455, 1438, 1416, 1375, 1360, 1333, 1286, 1253, 1170, 1135, 1103, 1076, 1049, 1021, 971, 938, 901, 832, 808, 776, 748, 699, 663, 637, 614, 571, 535, 498.

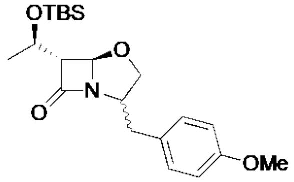

12d: The **General Procedure B** was applied using **10d** (0.2 mmol, 78 mg). The diastereomeric ratio (5.6:1) was determined by ^{1}H NMR analysis of the reaction mixture, integrating the signals at 5.19 ppm *Trans* (1H) and 5.15 ppm *Cis* (1H). Only the trans diastereomer (*trans*-**12d**) was isolated by flash chromatography (9:1 Hex:AcOEt) as a colourless oil, giving a product yield of 65% (0.13 mmol, 51 mg). *trans*-**12d**: ^{1}H NMR (400 MHz, $CDCl_3$) δ 7.24 – 7.19 (m, 2H), 6.87 – 6.80 (m, 2H), 5.19 (s, 1H), 4.17 (qd, *J* = 6.3, 4.6 Hz, 1H), 4.08 – 4.00 (m, 1H), 3.79 (s, 3H), 3.75 – 3.68 (m, 1H), 3.64 – 3.49 (m, 2H), 3.01 (dd, *J* = 4.7, 0.8 Hz, 1H), 2.84 – 2.76 (m, 1H), 1.25 (d, *J* = 6.3 Hz, 3H), 0.88 (s, 9 H), 0.06 (s, 6H). ^{13}C NMR (100 MHz, $CDCl_3$) δ 176.0, 158.4, 130.2, 129.7, 114.0, 87.3, 74.6, 65.9, 64.2, 62.6, 55.2, 33.5, 25.7, 22.3, 17.9, -4.4, -5.1. ATR-FTIR: *ν* (cm^{-1}) 2954, 2930, 2897, 2856, 1770, 1612, 1584, 1513, 1471, 1463, 1442, 1375, 1360, 1350, 1326, 1301, 1287, 1247,1174, 1151, 1137, 1105, 1077, 1035, 1008, 972, 937, 902, 831, 806, 775, 749, 739, 683,663, 613, 532, 436. HRMS *m/z* = $[M+Na]^+$ calcd for $C_{21}H_{33}NNaO_4Si^+$ 414.2077, found 414.2072.

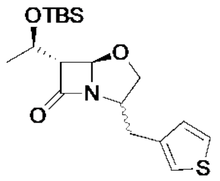

12e: The **General Procedure B** was applied using **10e** (0.2 mmol, 73 mg). The diastereomeric ratio (1.6:1) was determined by ^{1}H NMR analysis of the reaction mixture, integrating the signals at 5.21 ppm *Trans* (1H) and at 5.16 ppm *Cis* (1H). The crude was purified by flash chromatography (90:10 n-Hex:EtOAc). The *trans* diastereomer (20 mg) and the *cis* diastereomer (9 mg) were isolated as colourless oils, giving an overall product yield of 40% (29 mg, 0.079 mmol). *trans*-**12e**: ^{1}H NMR (400 MHz, $CDCl_3$) δ 7.28 – 7.26 (m, 1H), 7.12 (ddt, *J* = 2.9, 1.4, 0.7 Hz, 1H), 7.03 (dd, *J* = 5.0, 1.3 Hz, 1H), 5.21 (s, 1H), 4.17 (qd, *J* = 6.3, 4.5 Hz, 1H), 4.11 (dd, *J* = 8.3, 5.5 Hz, 1H), 3.71 (td, *J* = 7.1, 0.9 Hz, 1H), 3.65 – 3.54 (m, 2H), 3.01 (d, *J* = 4.5 Hz, 1H), 2.99 – 2.87 (m, 1H), 1.25 (d, *J* = 6.4 Hz, 3H), 0.88 (d, *J* = 0.6 Hz, 9H), 0.06 (d, *J* = 0.6 Hz, 6H). ^{13}C NMR (100 MHz, $CDCl_3$) δ 176.1, 138.4, 128.3, 125.9, 122.1, 87.4, 74.9, 66.1, 64.3, 61.9, 29.3, 25.8, 22.5, 21.6, 18.1, -4.23, -4.94. ATR-FTIR: ν (cm^{-1}) 2954, 2929, 2884, 2856, 1769, 1635, 1472, 1462, 1442, 1410, 1375, 1360, 1350, 1328, 1288, 1252, 1169, 1152, 1137, 1101, 1077, 1046, 1007, 972, 938, 903, 831, 808, 774, 739, 688, 663, 647, 617, 588, 576, 532, 504, 455, 434. HPLC-MS R_t = 13.7 min, *m/z* = 390.0 [M+Na]$^+$. HRMS *m/z* = [M+Na]$^+$ calcd for $C_{18}H_{29}NNaO_3SSi^+$ 390.1535, found 390.1531. *cis*-**12e**: ^{1}H NMR (400 MHz, $CDCl_3$) δ 7.29 – 7.24 (m, 2H), 7.10 (dt, *J* = 3.0, 1.1 Hz, 1H), 6.99 (dd, *J* = 4.9, 1.3 Hz, 1H), 5.16 (s, 1H), 4.29 (qd, *J* = 6.8, 4.9 Hz, 1H), 4.22 – 4.13 (m, 2H), 3.81 (dd, *J* = 8.6, 4.9 Hz, 1H), 2.97 (dd, *J* = 4.3, 0.7 Hz, 1H), 2.89 – 2.70 (m, 2H), 1.25 (d, *J* = 6.2 Hz, 4H), 0.87 (s, 9H), 0.06 (s, 3H), 0.05 (s, 3H). ^{13}C NMR (100 MHz, $CDCl_3$) δ 178.3, 137.6, 128.5, 125.9, 122.2, 86.6, 74.9, 65.5, 64.3, 58.4, 33.2, 25.8, 22.6, 18.1, -4.26, -4.95. ATR-FTIR: *v* (cm^{-1}) 3101, 2953, 2929, 2884, 2856, 1776, 1472, 1463, 1443, 1409, 1374, 1361, 1330, 1288, 1252, 1171, 1136, 1105, 1075, 1039, 1002, 939, 901, 830, 809, 774, 741, 688, 663, 628, 574, 535, 451. HPLC-MS (ESI$^+$). R_t= 13.4 min, *m/z* = 390.0 [M+Na]$^+$.

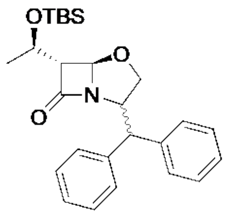

12f: The **General Procedure B** was applied using **10f** (0.2 mmol, 87 mg). The diastereomeric ratio (20:1) was determined by ^{1}H NMR analysis of the reaction mixture, integrating the signals at 5.12 ppm *Cis* (1H) and 5.32 ppm *Trans* (1H). Only the *trans* diastereomer (*trans*-**12f**) was isolated by flash chromatography (9:1 n-Hex:AcOEt) as a colourless oil, giving a product yield of 31% (0.06 mmol, 27 mg). *trans*-**12f**: ^{1}H NMR (400 MHz, $CDCl_3$) δ 7.50 – 7.44 (m, 2H), 7.32 – 7.26 (m, 4H), 7.24 – 7.15 (m, 4H), 5.32 (s, 1H), 4.67 (d, *J* = 11.6 Hz, 1H), 4.18 – 4.07 (m, 2H), 3.83 (dd, *J* = 8.4, 5.6 Hz, 1H), 3.57 (dd, *J* = 9.5, 8.4 Hz, 1H), 2.97 (d, *J* = 4.6 Hz, 1H), 1.22 (d, *J* = 6.3 Hz, 3H), 0.83 (s, 10H), 0.02 (s, 3H), -0.05 (s, 3H). ^{13}C NMR (100 MHz, $CDCl_3$) δ 176.2, 142.4, 142.0, 129.0, 128.5, 128.0, 127.9, 127.3, 126.6, 88.0, 74.0, 66.6, 64.8, 64.4, 49.7, 25.8, 22.5, 21.7, -4.2, -5.1. ATR-FTIR: *v* (cm^{-1}) 3028, 2976, 2960, 2945, 2925, 2887, 2852, 1775, 1736, 1717, 1669, 1653,

1598, 1495, 1470, 1456, 1380, 1360, 1352, 1341, 1323, 1278, 1251, 1193, 1172, 1135, 1104, 1071, 1032, 1013, 998, 966, 934, 898, 829, 806, 778, 741, 690, 658, 636, 620, 599, 536, 501, 441. HRMS *m/z* = $[M+Na]^+$ calcd for $C_{26}H_{35}NNaO_3Si^+$ 460.2284, found 460.2282.

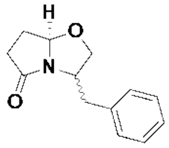

15: The **General Procedure B** was applied using **14** (0.2 mmol, 42 mg). The crude was purified by flash chromatography on silica gel (10:90 to 70:30 EtOAc/n-Hex), obtaining the product (yellow oil, 15 mg, 0.069 mmol) as a single diastereomer **15** in 35% yield. ^{1}H NMR (400 MHz, $CDCl_3$) δ 7.35 – 7.29 (m, 2H), 7.25 (dt, *J* = 9.7, 2.5 Hz, 4H), 5.04 (dd, *J* = 6.2, 2.6 Hz, 1H), 4.45 – 4.32 (m, 1H), 4.08 (dd, *J* = 8.8, 7.1 Hz, 1H), 3.67 (dd, *J* = 8.8, 6.4 Hz, 1H), 3.05 (dd, *J* = 13.9, 5.8 Hz, 1H), 2.79 (dd, *J* = 13.9, 8.3 Hz, 1H), 2.66 (ddd, *J* = 17.6, 10.4, 7.2 Hz, 1H), 2.51 (ddd, *J* = 17.6, 10.5, 4.5 Hz, 1H), 2.36 (dddd, *J* = 14.8, 10.5, 6.1, 4.5 Hz, 1H), 2.10 – 1.99 (m, 1H). ^{13}C NMR (100 MHz, $CDCl_3$) δ 179.5, 136.9, 129.4, 128.7, 126.9, 91.9, 77.4, 71.9, 55.5, 39.5, 31.8, 24.7. HPLC-MS t_R = 5.045 min; *m/z* = 218 $[M+H]^+$, 240 $[M+Na]^+$, 457 $[2M+Na]^+$. ATR-FTIR: *ν* (cm^{-1}) 3100, 2949, 2929, 2872, 1767, 1639, 1539, 1522, 1430, 1411, 1351, 1331, 1287, 1267, 1227, 1186, 1125, 1104, 1081, 1043, 1020, 984, 946, 855, 832, 774, 694, 660, 635, 587, 577, 544, 486. HRMS *m/z* = $[M+H]^+$ calcd for $C_{13}H_{16}NO_2^+$ 218.1176, found 218.1180.

NOE experiments for the determination of relative configuration

Figure S3. 1D-NOESY experiment on *cis*-**12c** (Minor diastereomer); selective irradiation at 5.21 ppm; mixing time= 2.5 s.

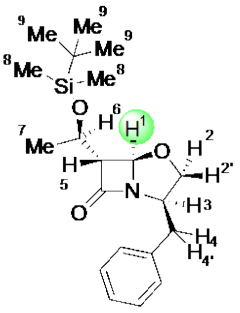

cis-**12c**

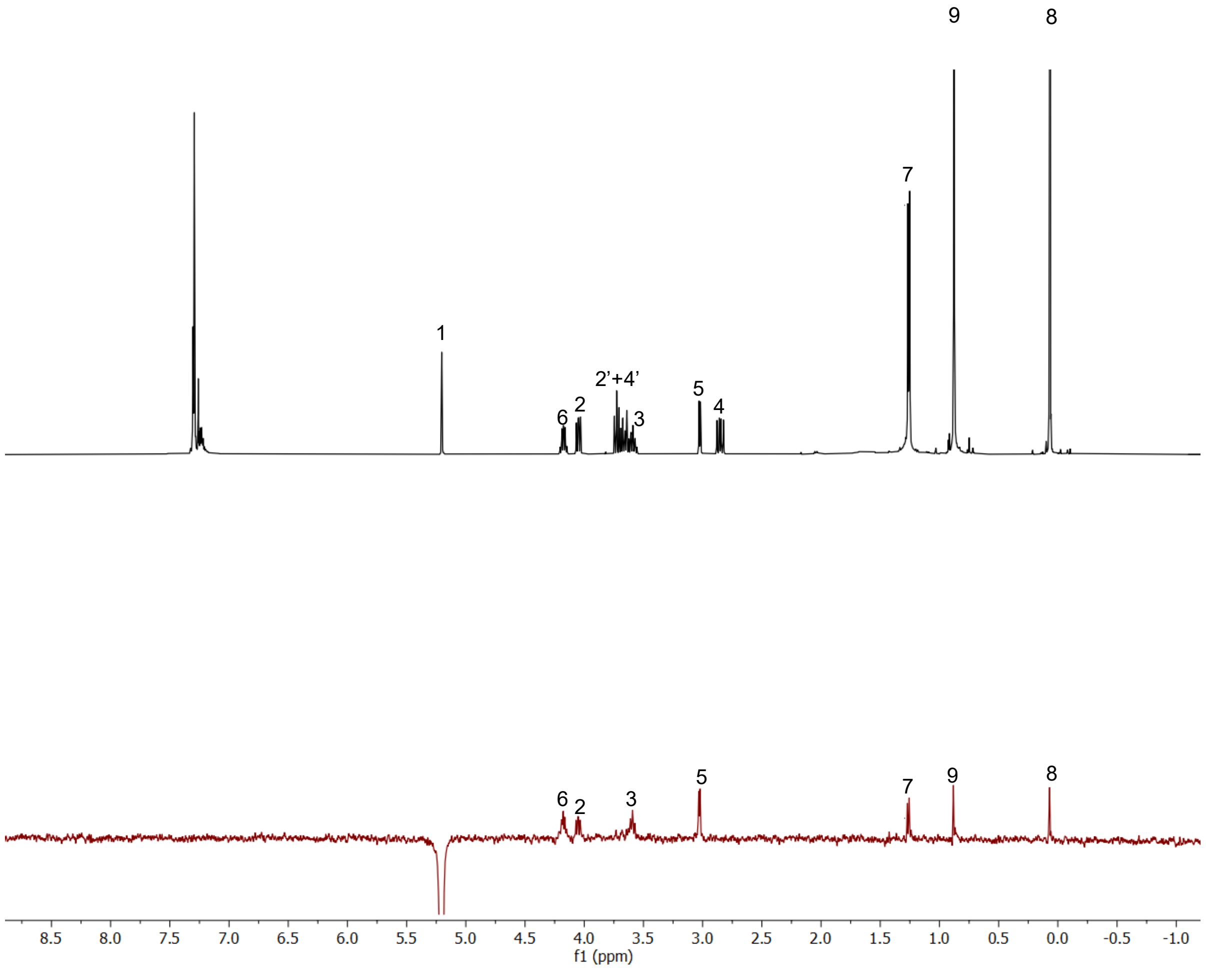

Figure S4. 1D-NOESY experiment on *trans*-**12c** (Major diastereomer); selective irradiation at 5.18 ppm; mixing time= 2.5 s.

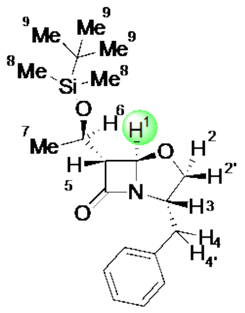

trans-**12c**

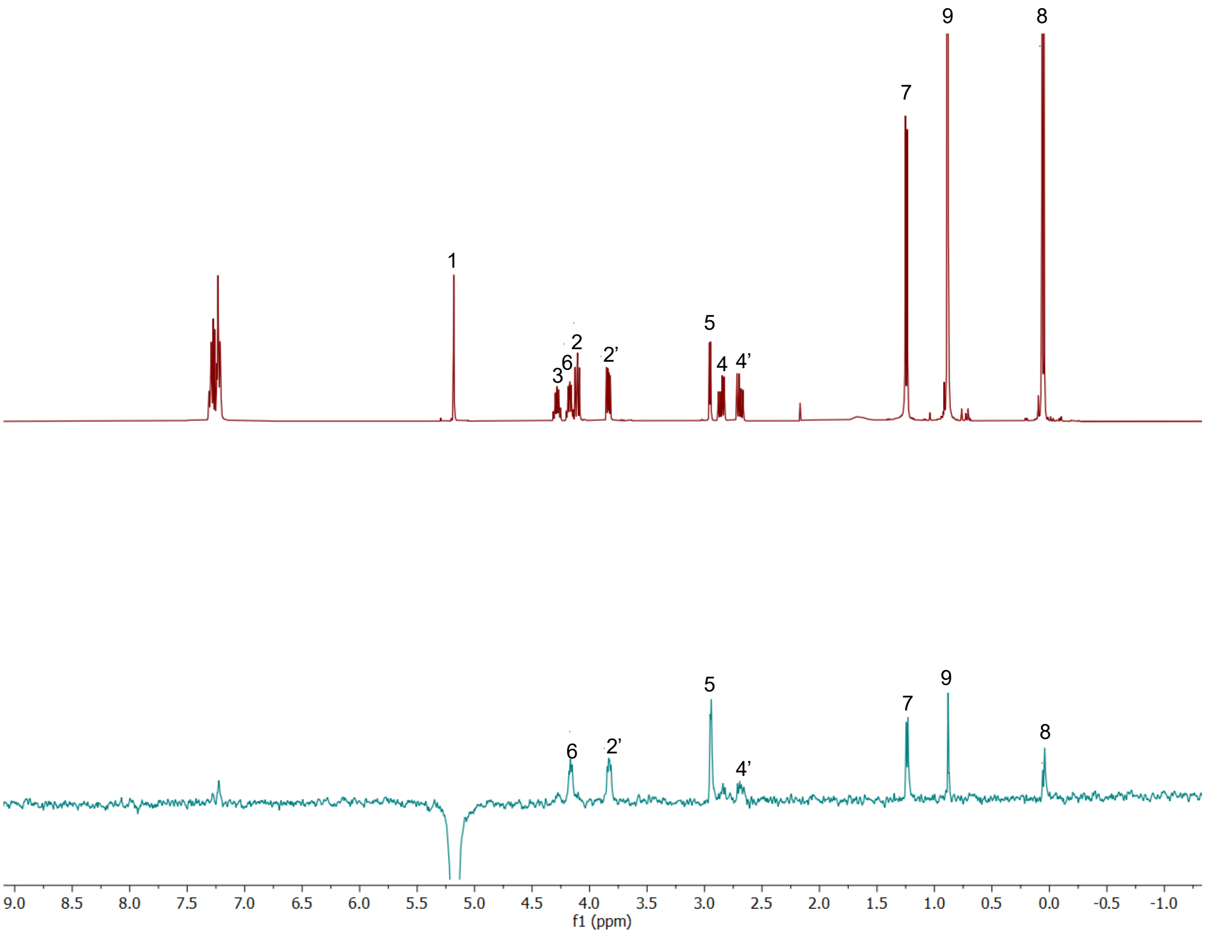

Copies of ^{1}H and ^{13}C NMR spectra

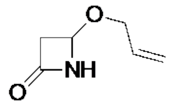

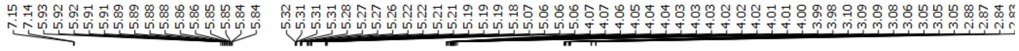

8a

^{1}H NMR ($CDCl_3$, 400 MHz)

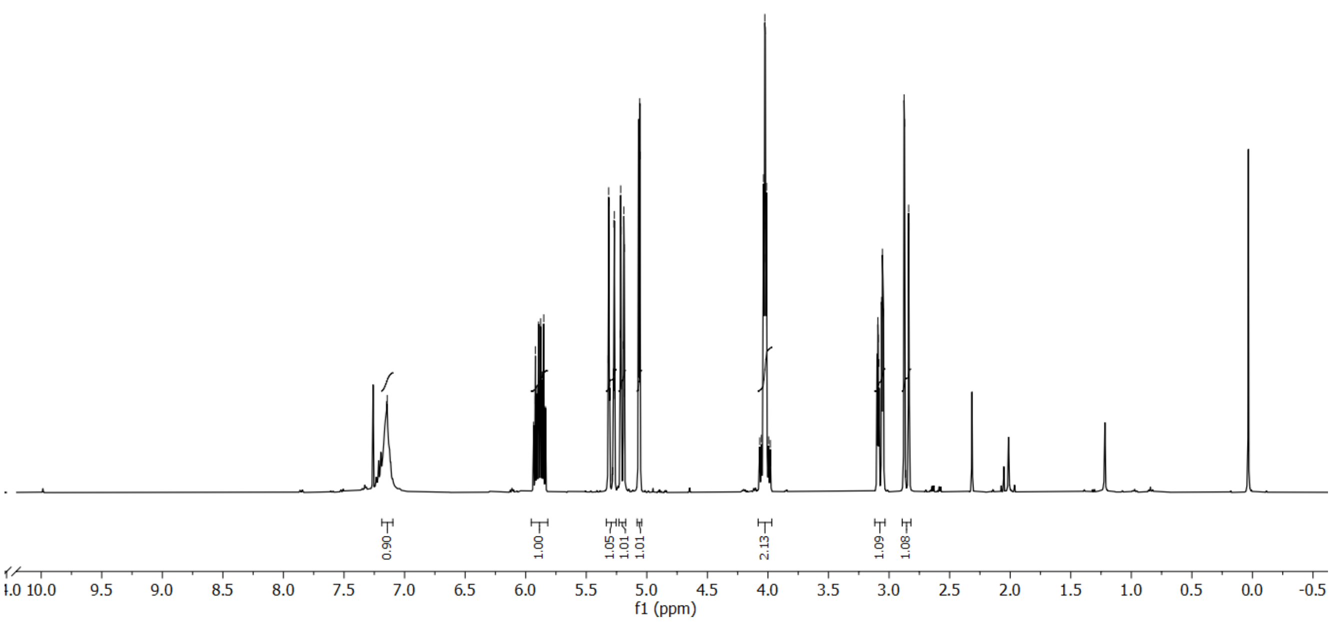

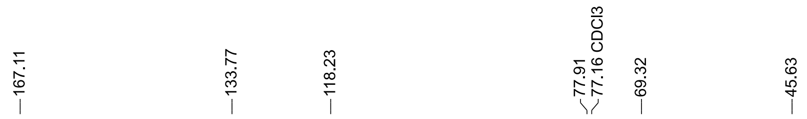

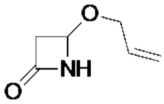

8a

^{13}C NMR ($CDCl_3$, 100 MHz)

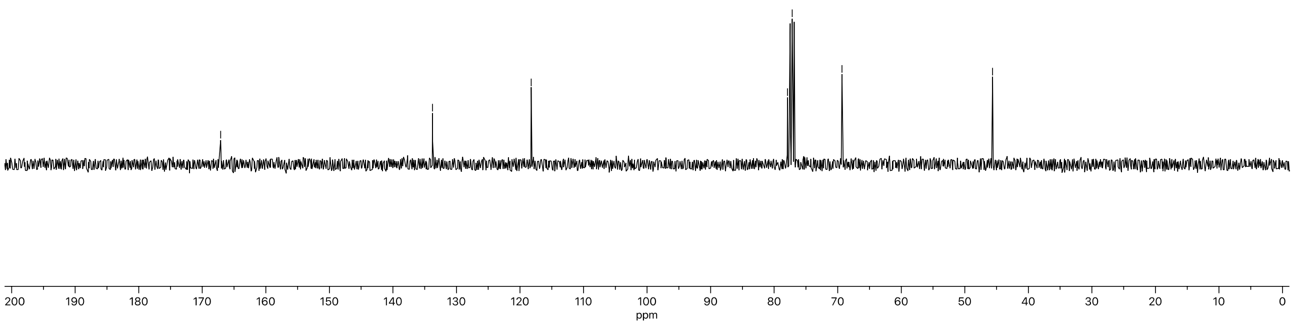

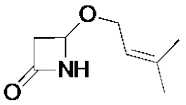

8b

^{1}H NMR ($CDCl_3$, 400 MHz)

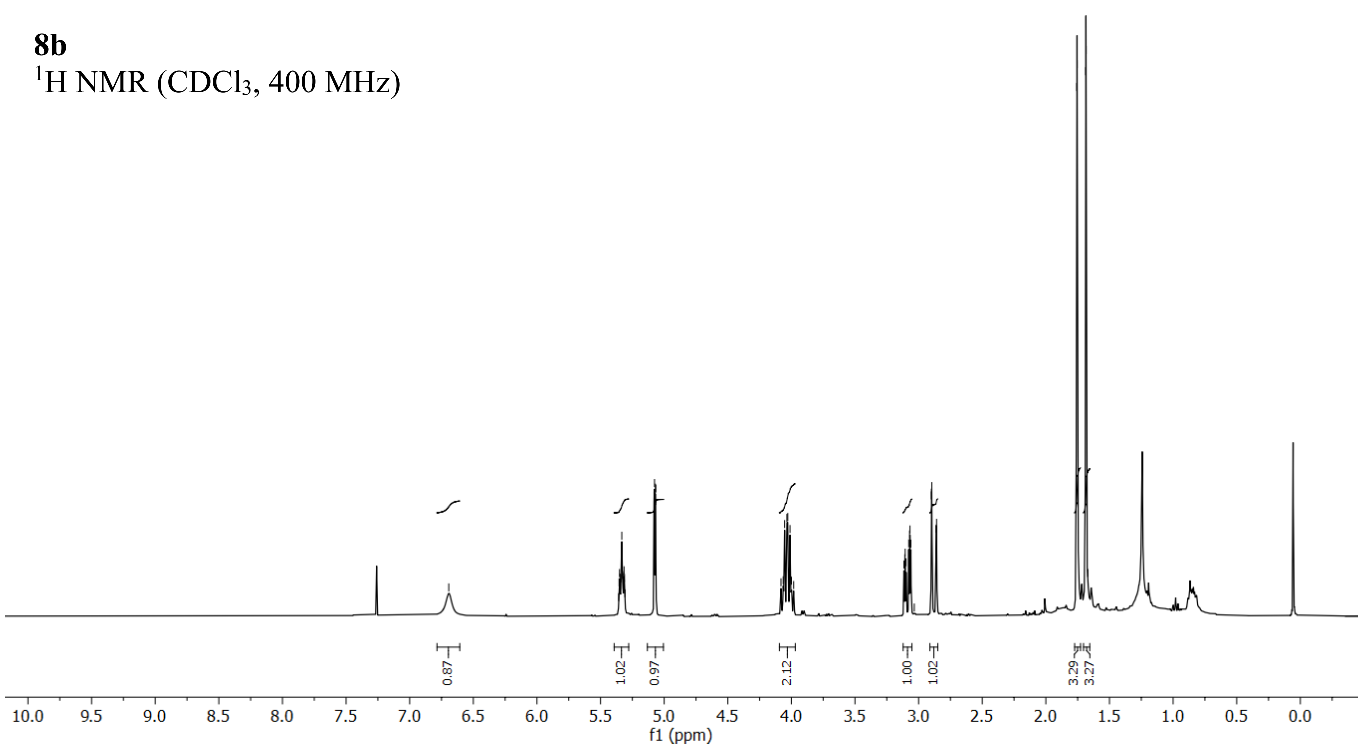

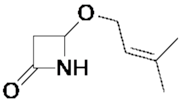

8b

^{13}C NMR ($CDCl_3$, 100 MHz)

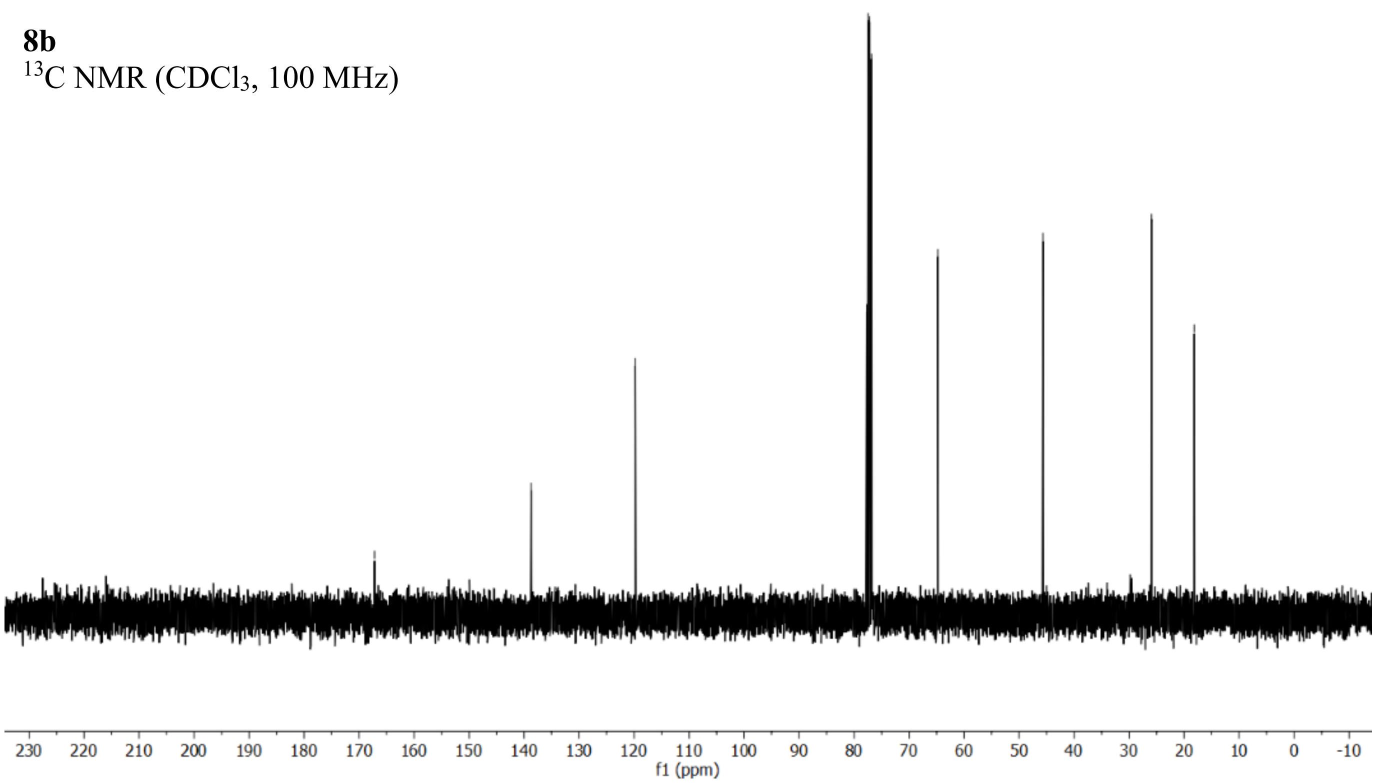

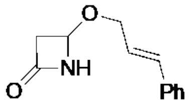

8c

^{1}H NMR ($CDCl_3$, 400 MHz)

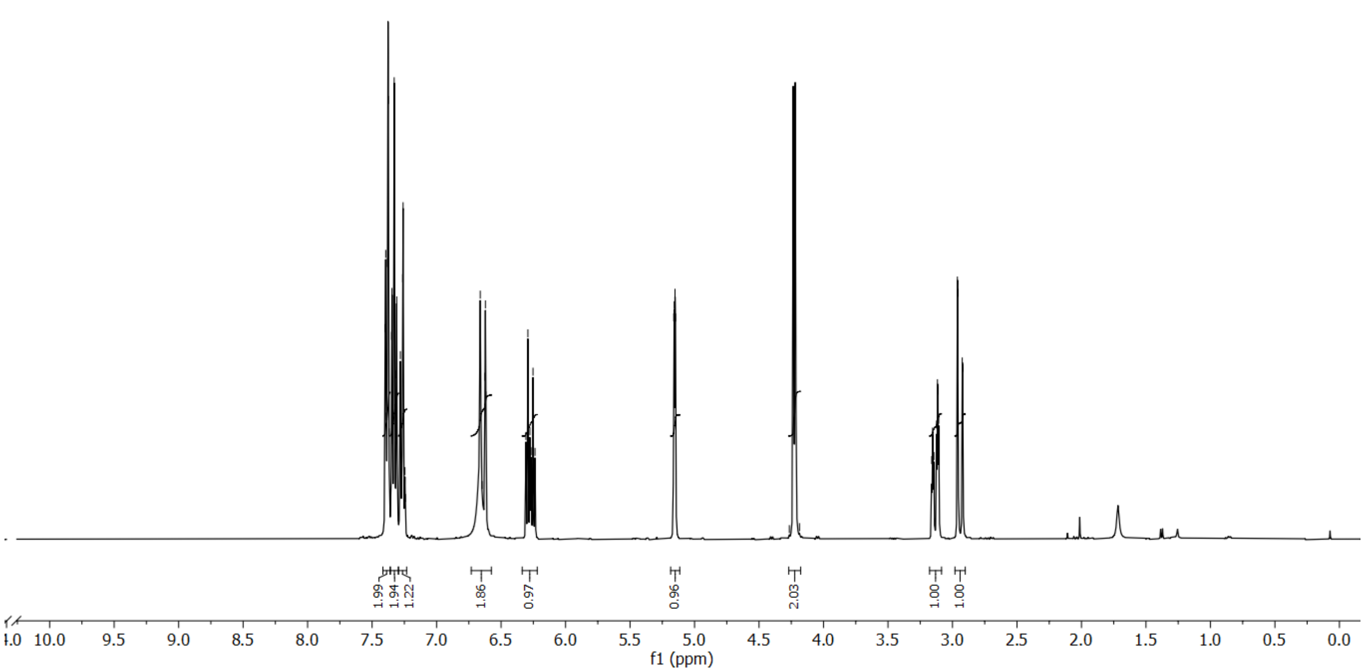

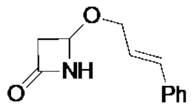

8c

^{13}C NMR ($CDCl_3$, 100 MHz)

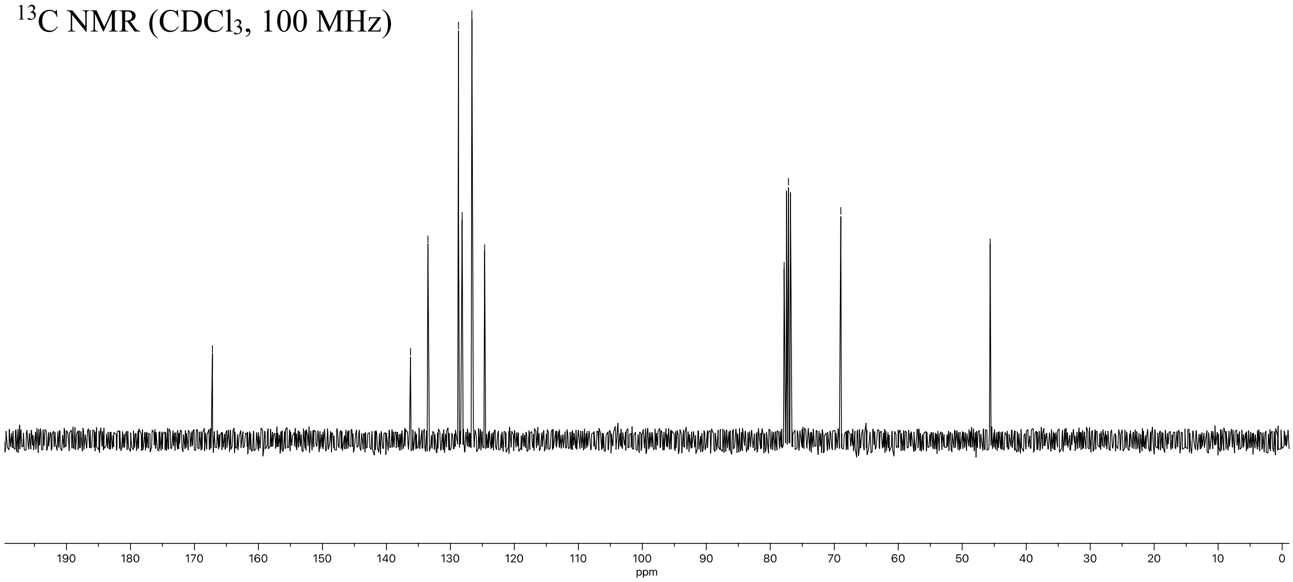

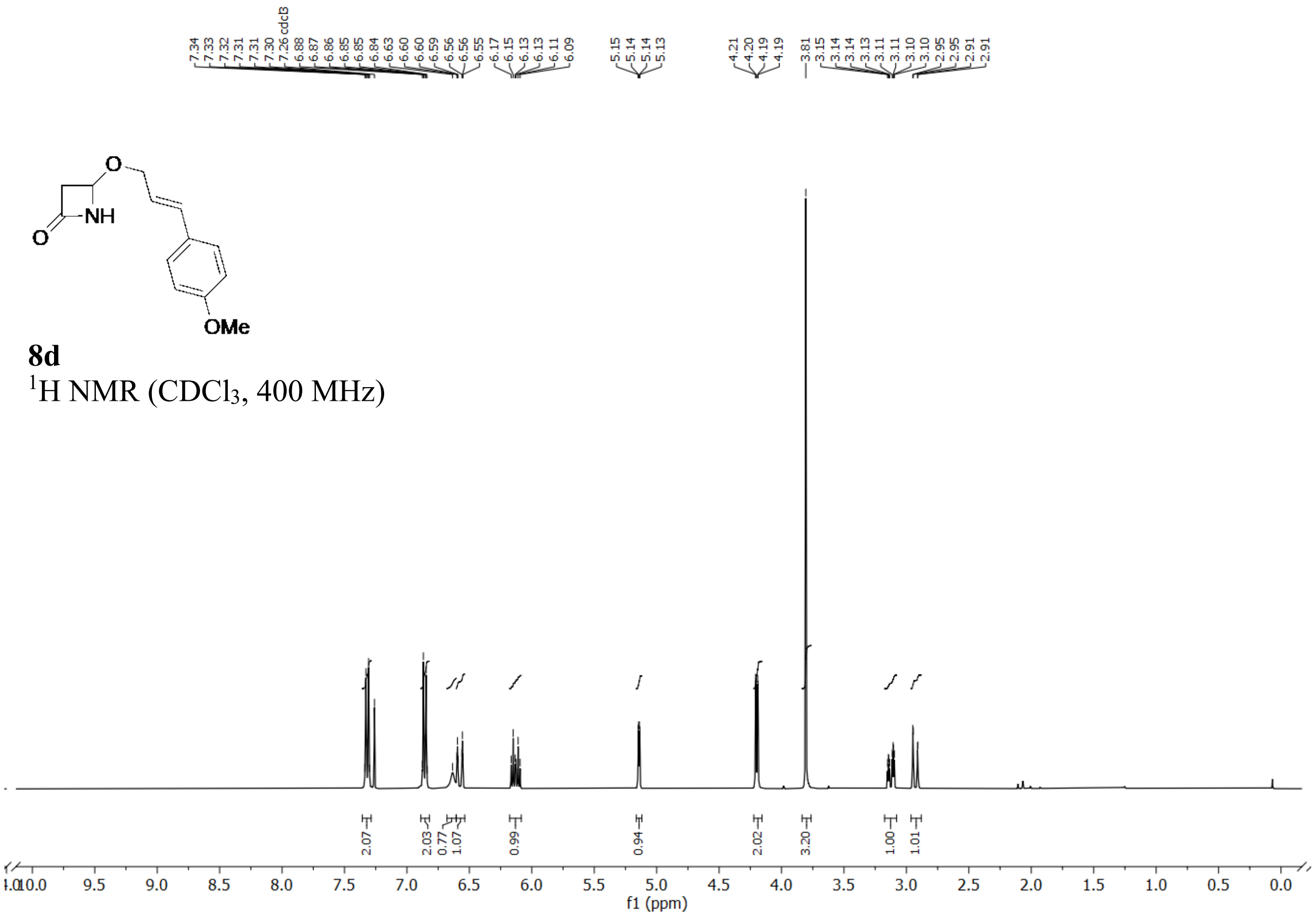

8d

^{1}H NMR ($CDCl_3$, 400 MHz)

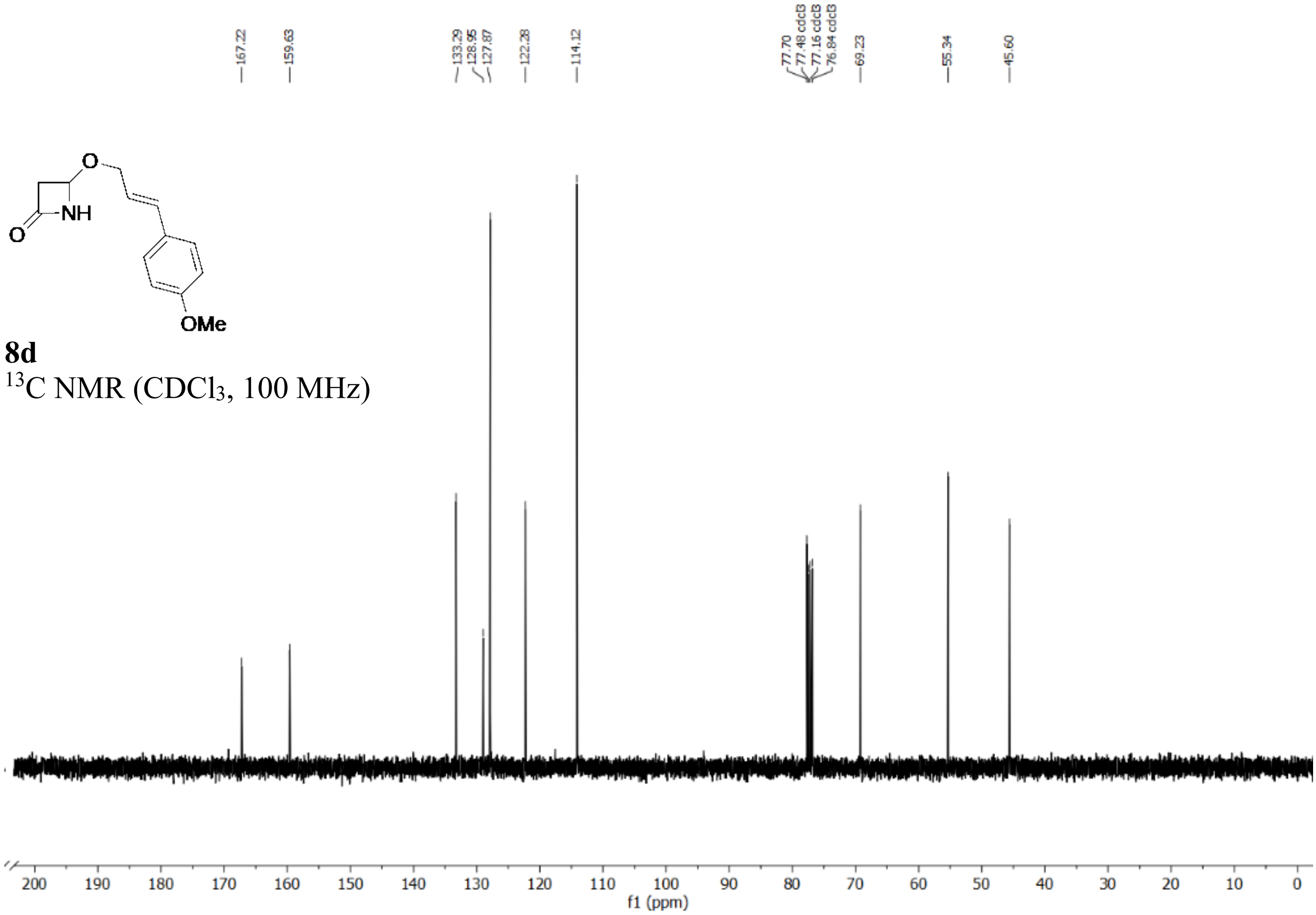

8d

^{13}C NMR ($CDCl_3$, 100 MHz)

8e

^{1}H NMR ($CDCl_3$, 400 MHz)

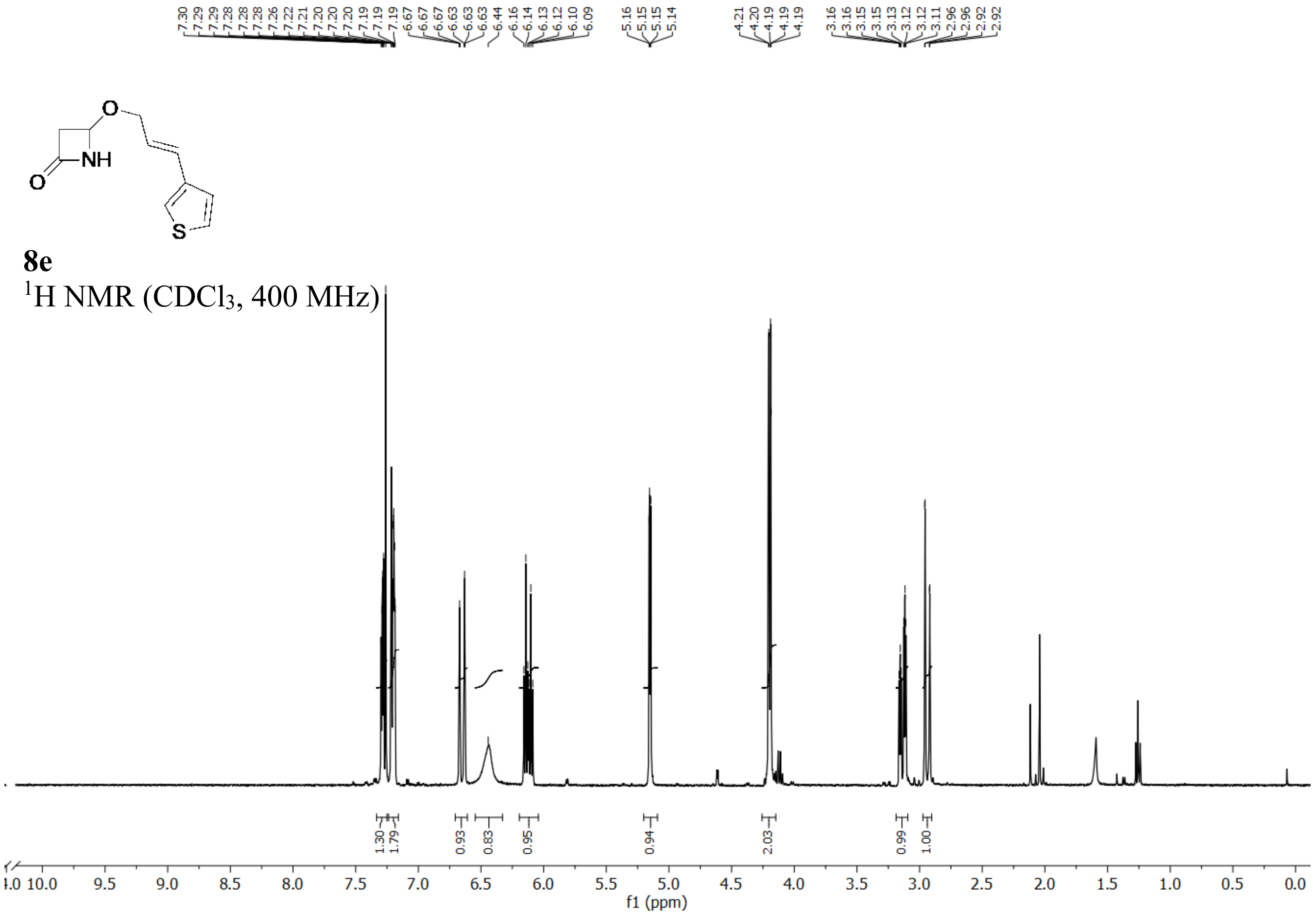

8e

^{13}C NMR ($CDCl_3$, 100 MHz)

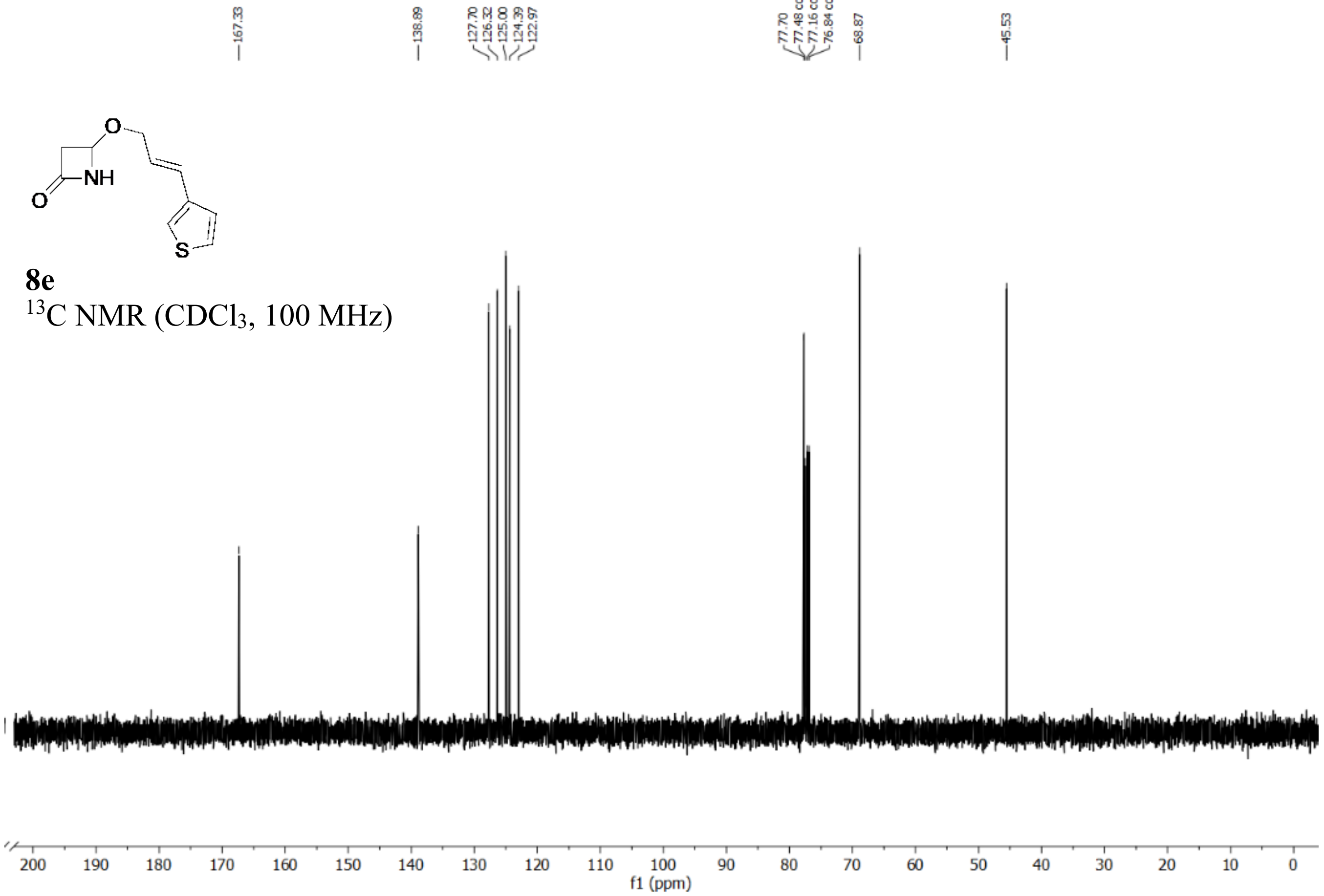

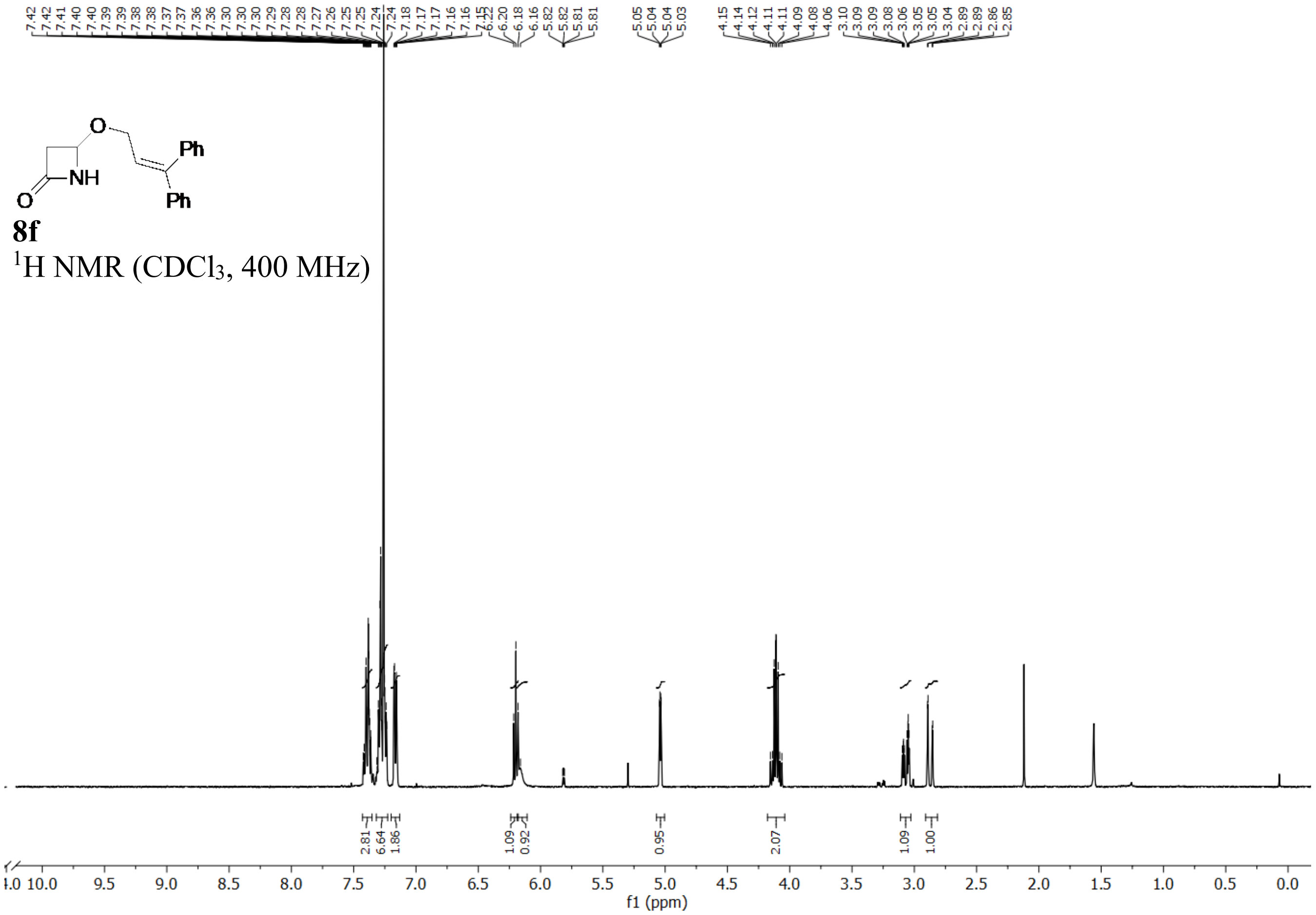

8f
^{1}H NMR ($CDCl_3$, 400 MHz)

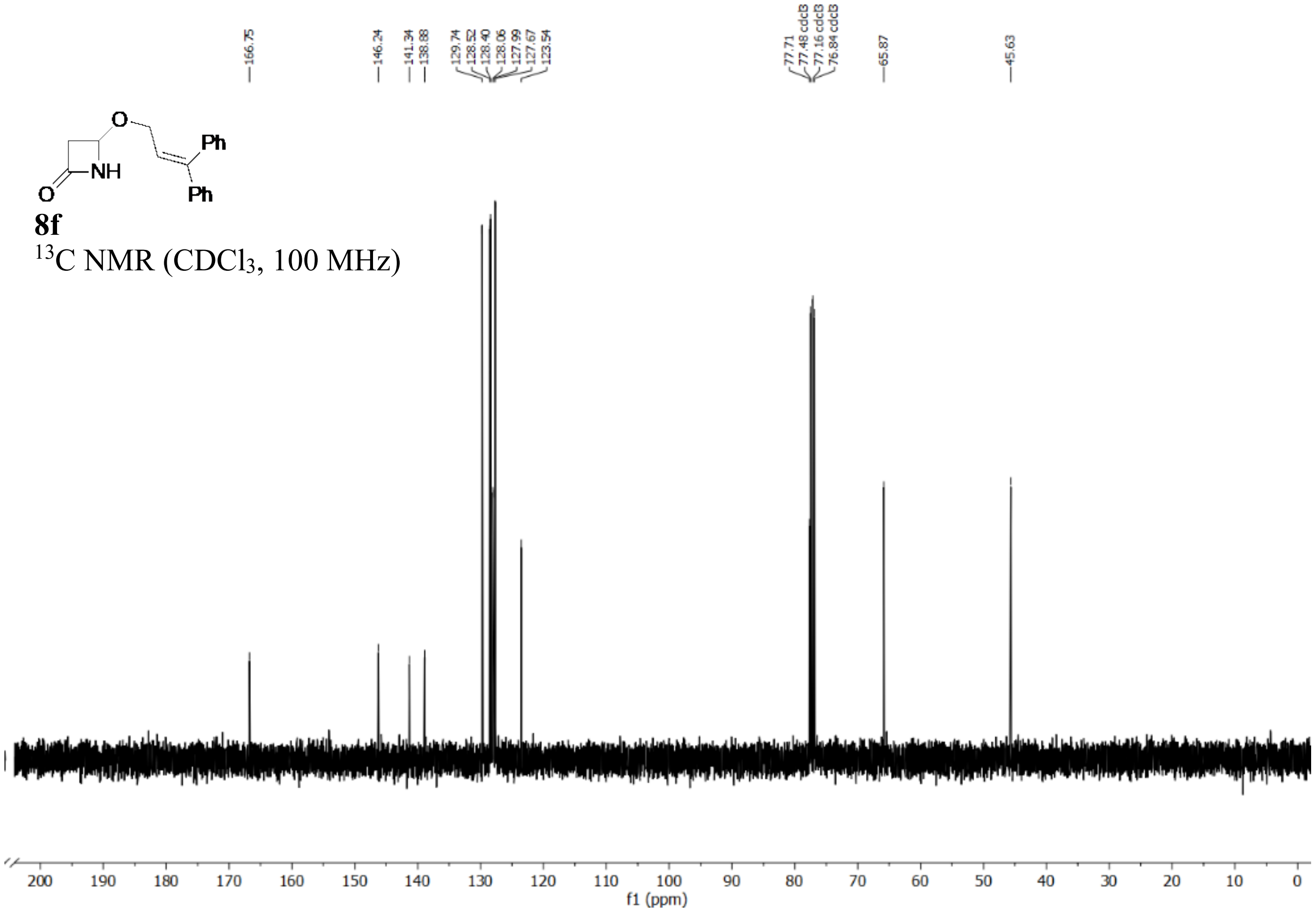

8f
^{13}C NMR ($CDCl_3$, 100 MHz)

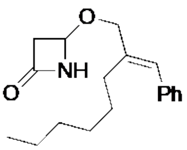

8g
^{1}H NMR ($CDCl_3$, 400 MHz)

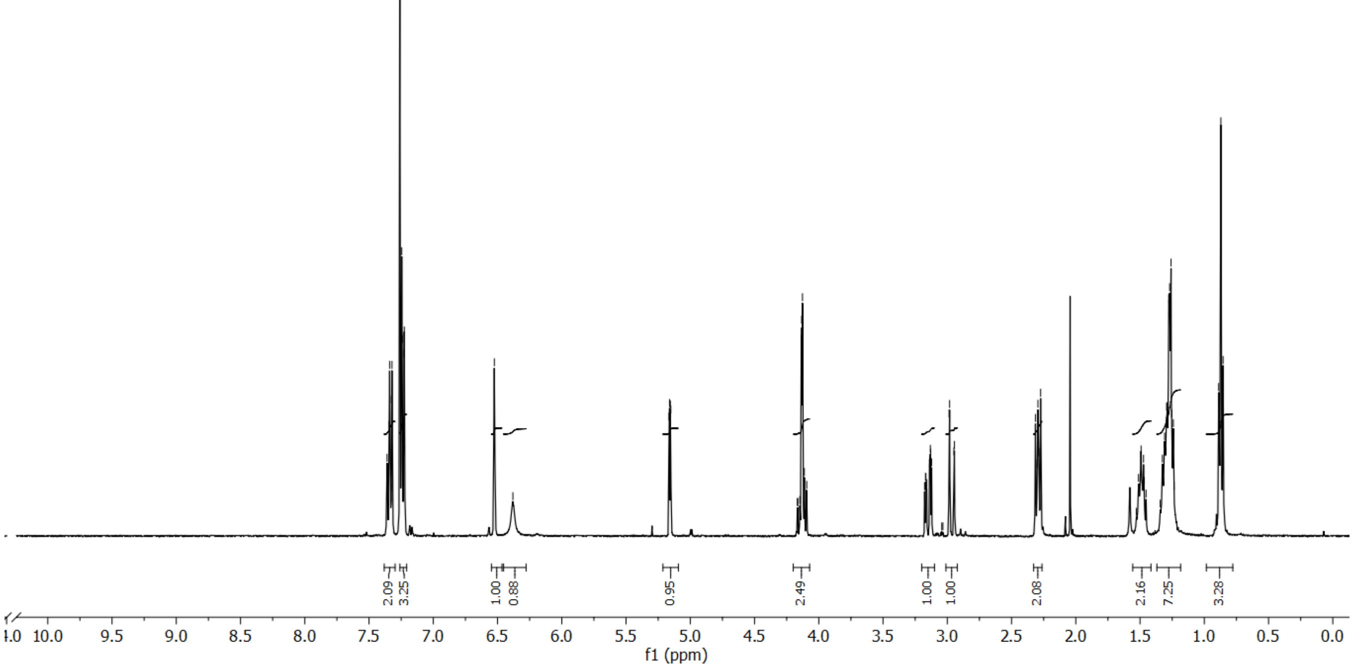

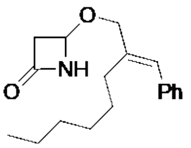

8g
^{13}C NMR ($CDCl_3$, 100 MHz)

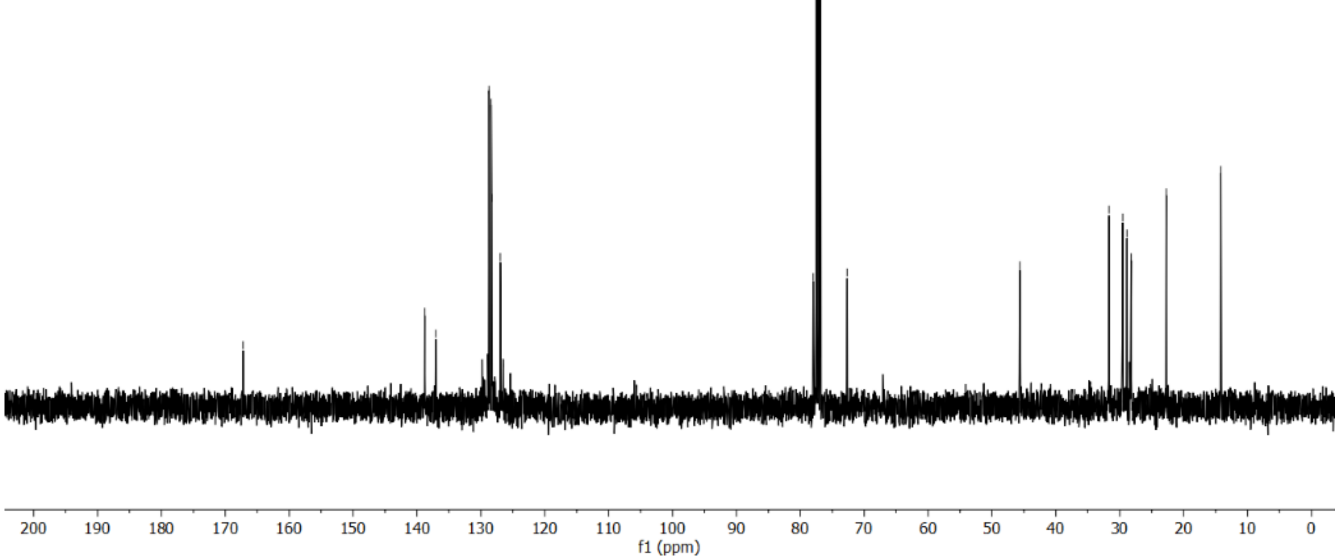

8h

^{1}H NMR ($CDCl_3$, 400 MHz)

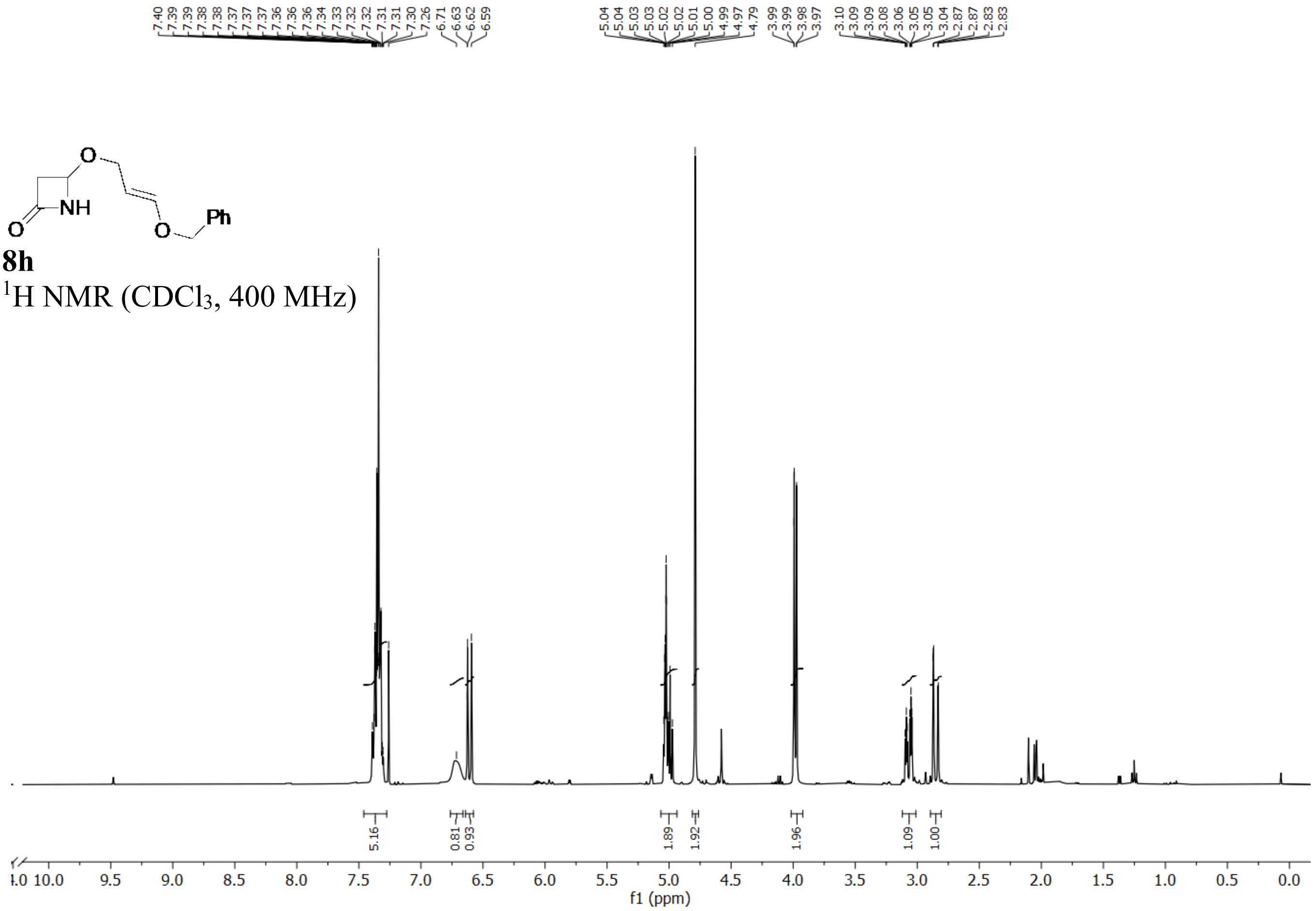

8h

^{13}C NMR ($CDCl_3$, 100 MHz)

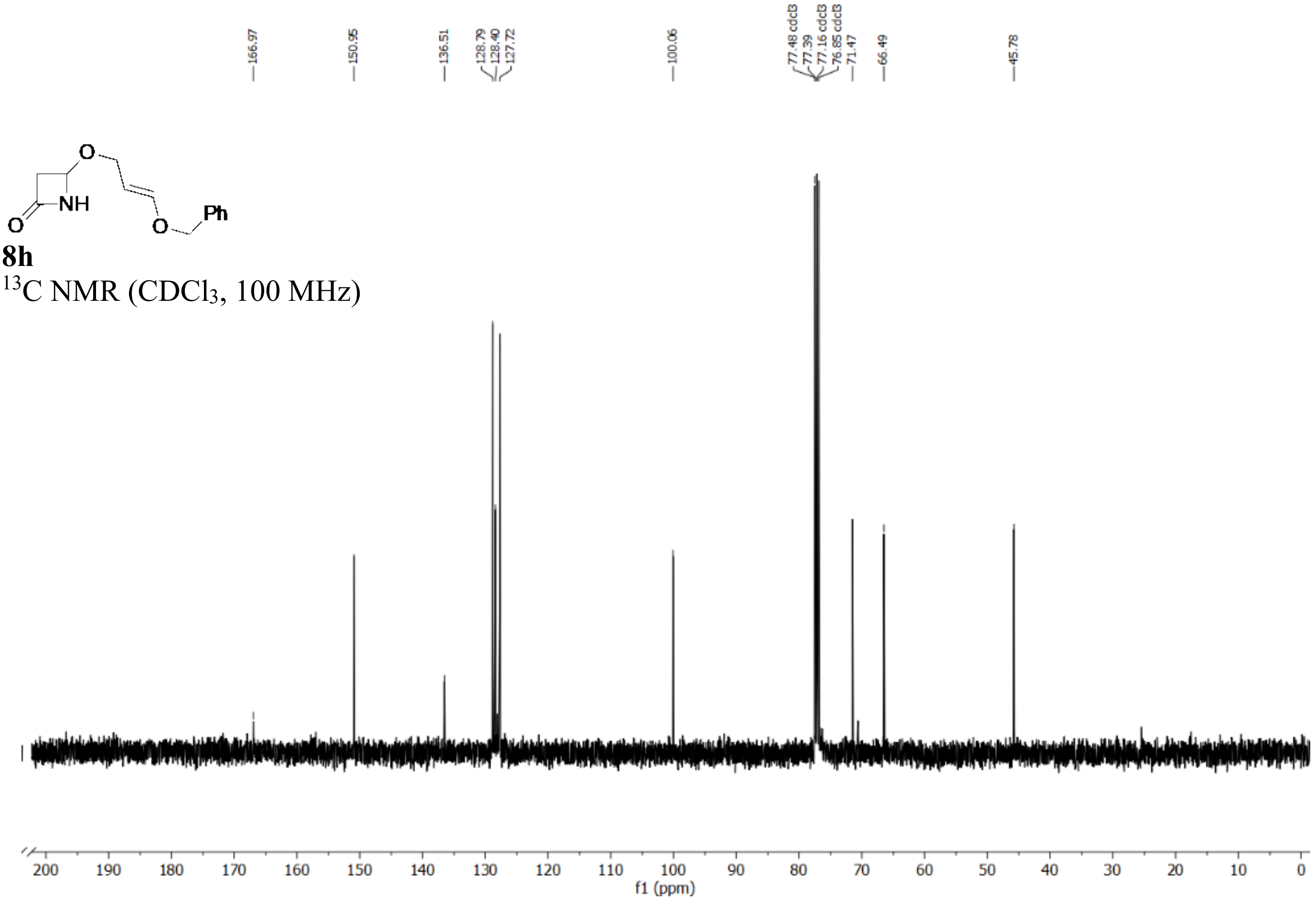

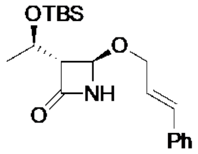

10c
^{1}H NMR ($CDCl_3$, 400 MHz)

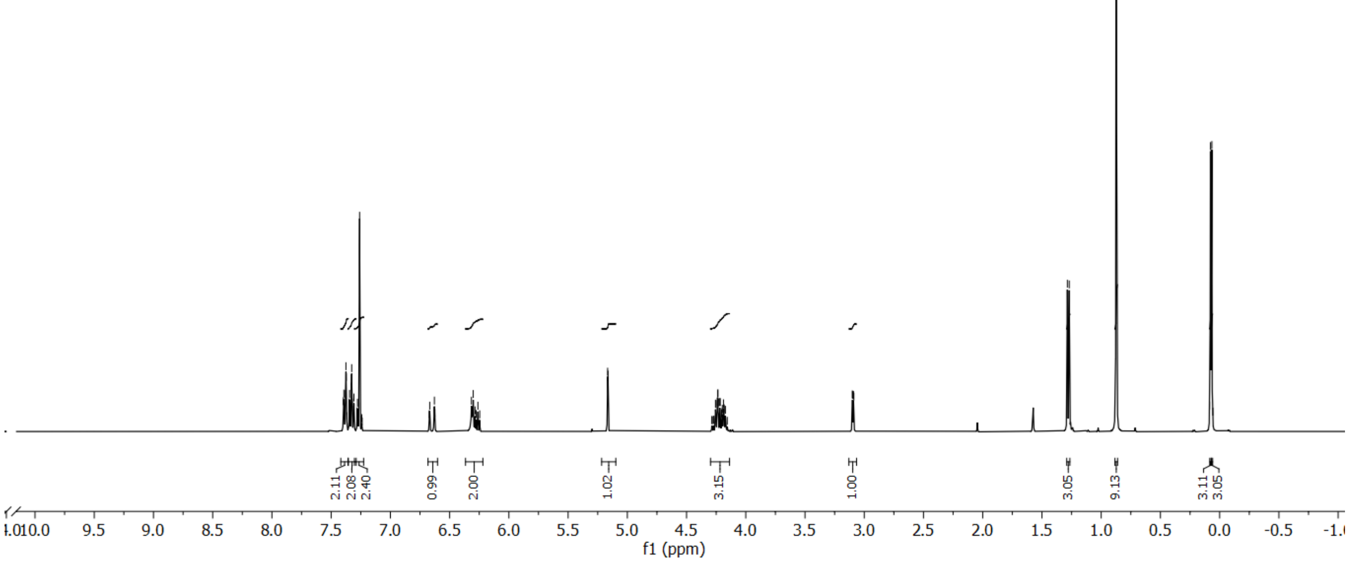

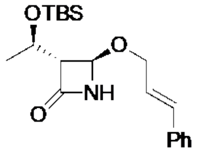

10c
^{13}C NMR ($CDCl_3$, 100 MHz)

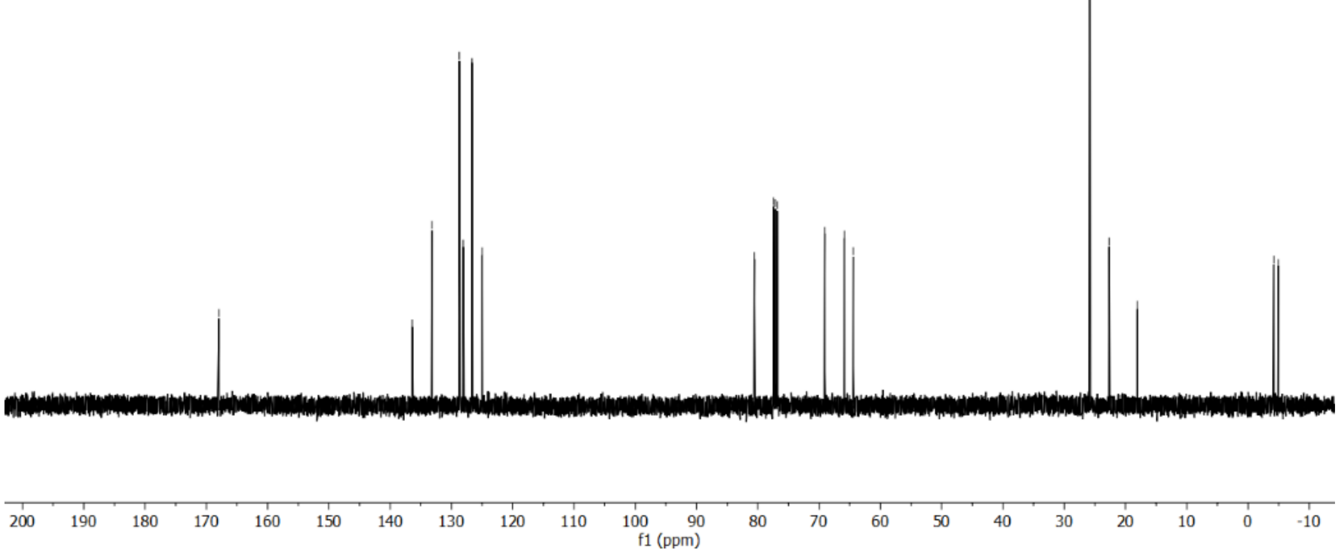

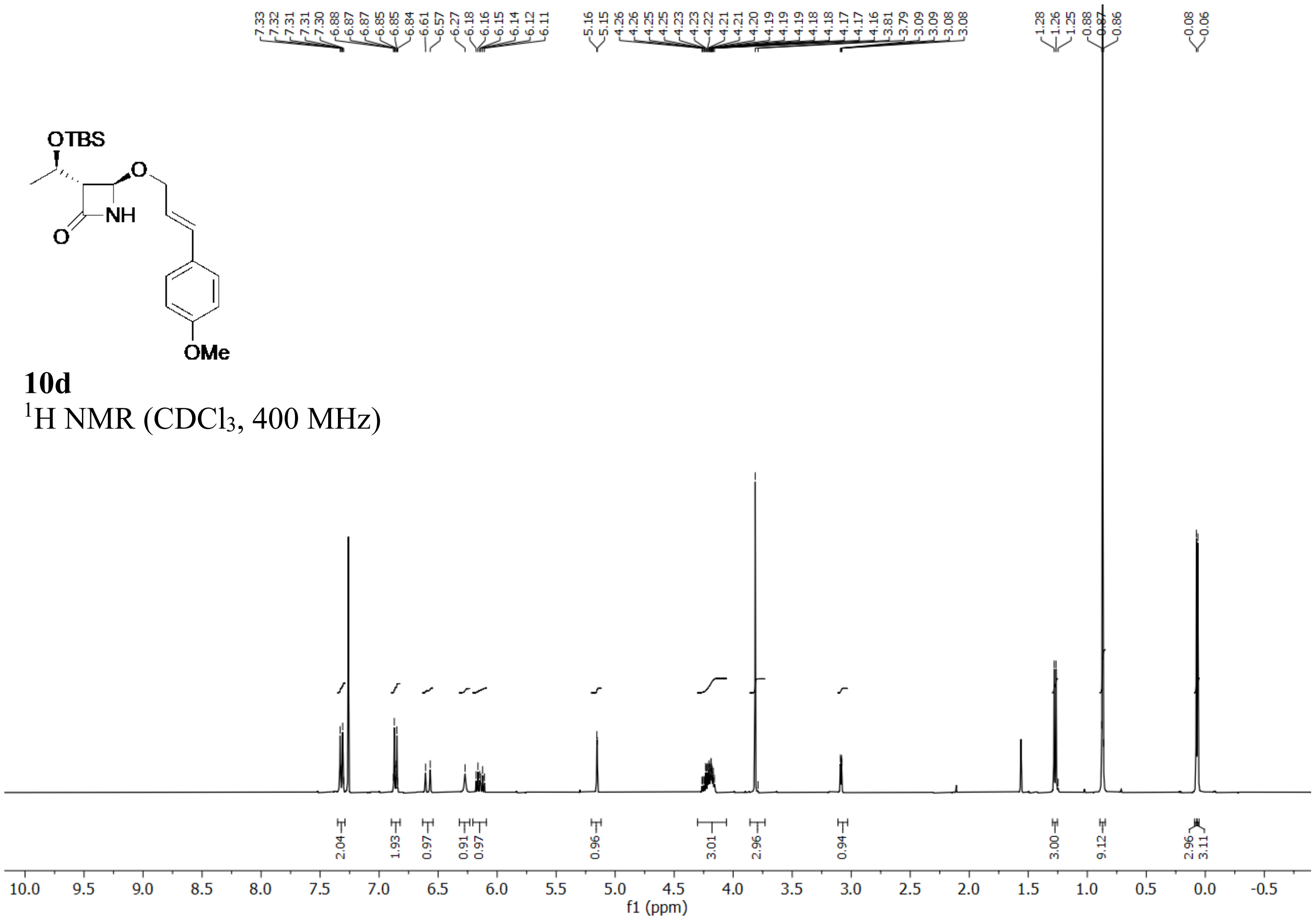

10d

^{1}H NMR ($CDCl_3$, 400 MHz)

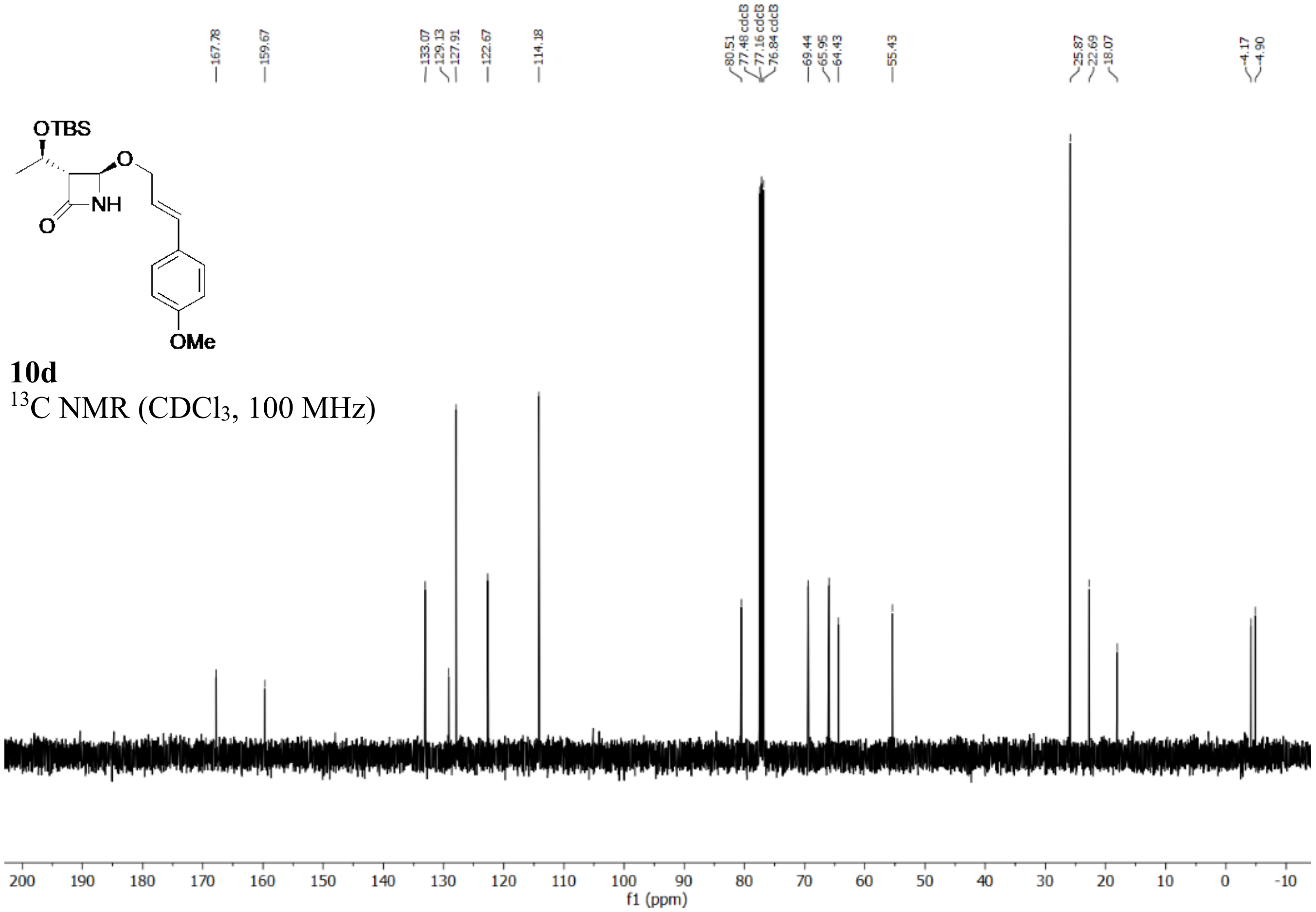

10d

^{13}C NMR ($CDCl_3$, 100 MHz)

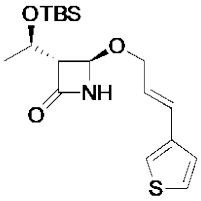

10e

^{1}H NMR ($CDCl_3$, 400 MHz)

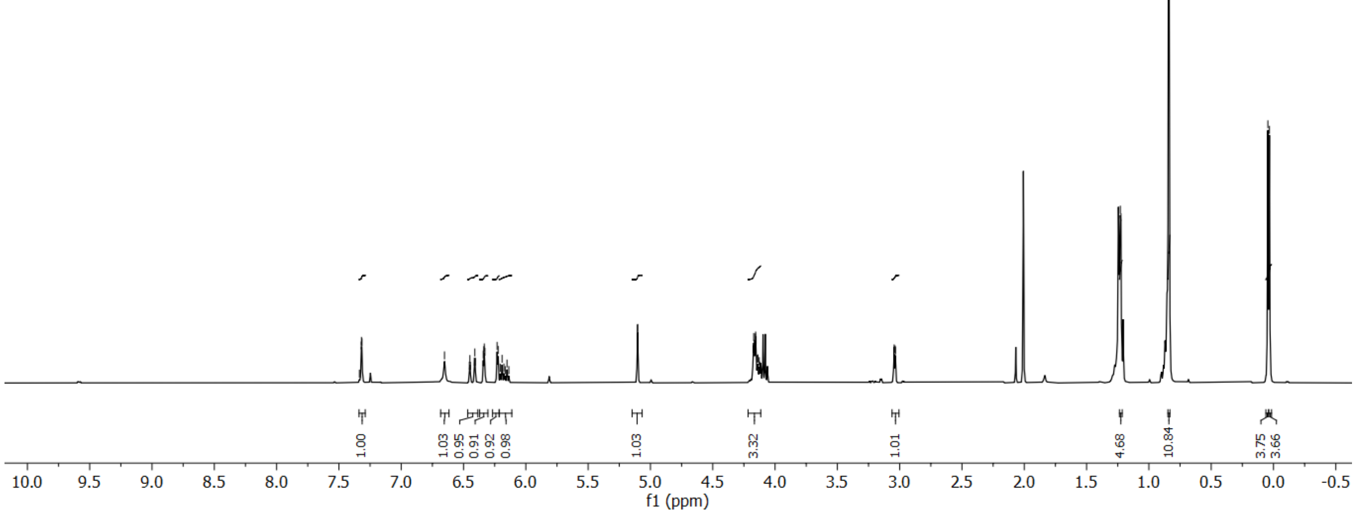

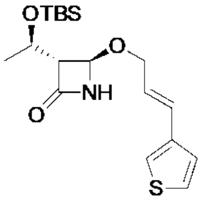

10e

^{13}C NMR ($CDCl_3$, 100 MHz)

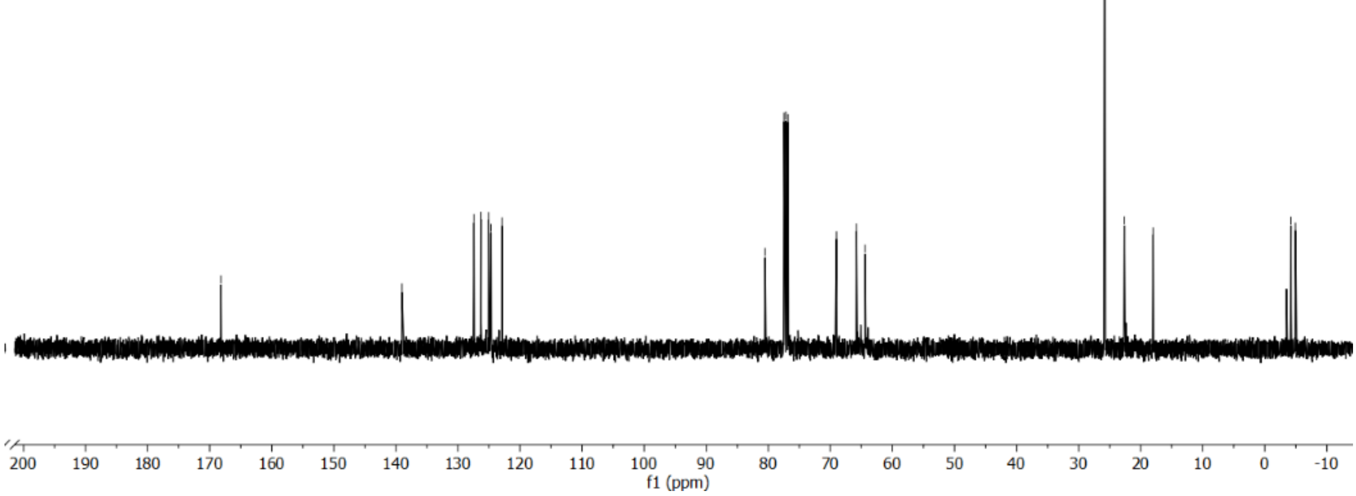

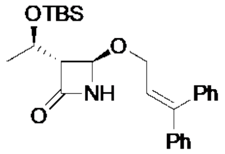

10f
^{1}H NMR ($CDCl_3$, 400 MHz)

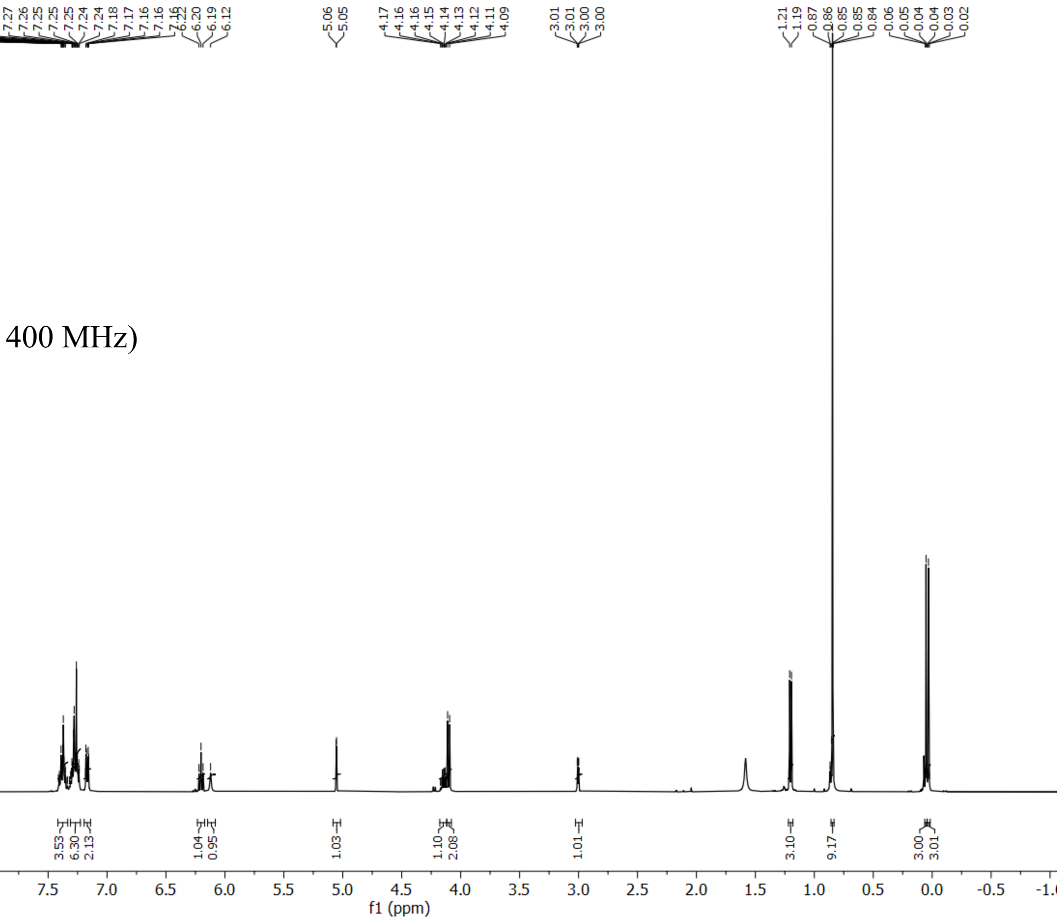

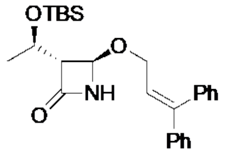

10f
^{13}C NMR ($CDCl_3$, 151 MHz)

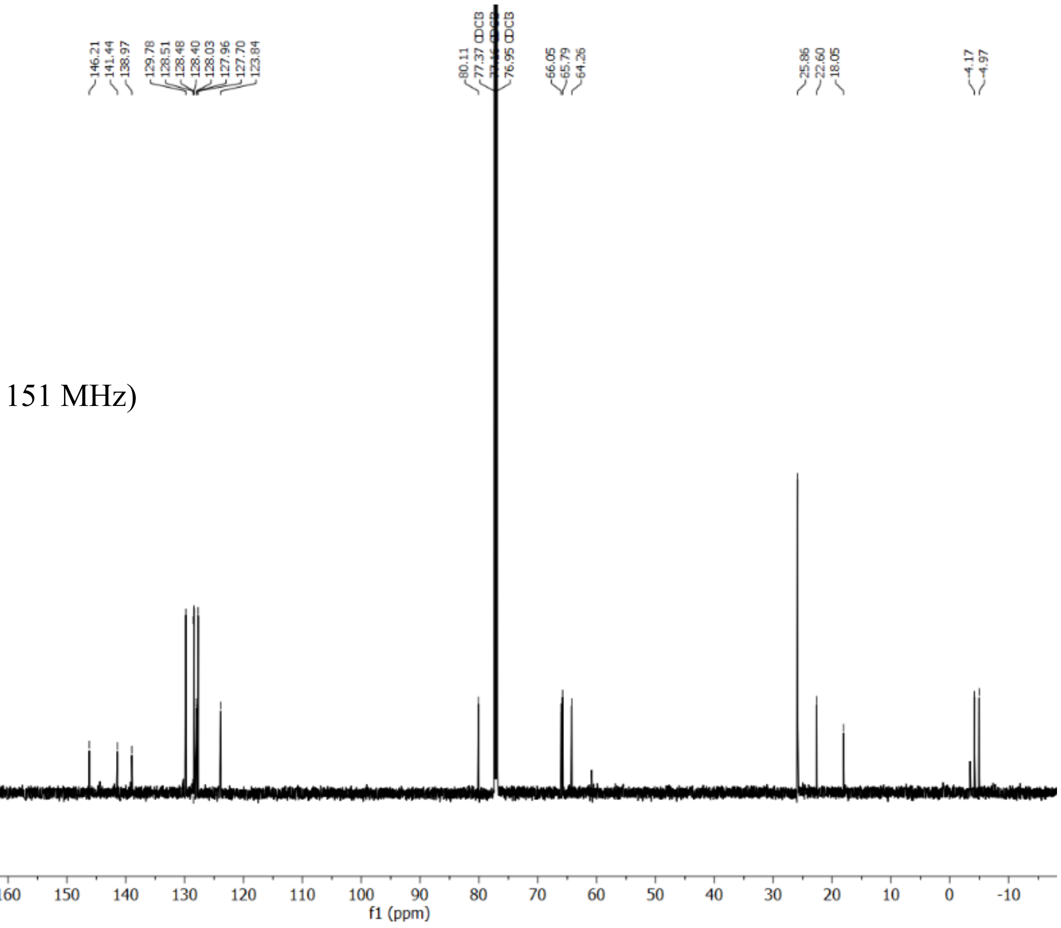

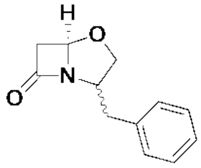

(±)-*trans*-**11c** (±)-*cis*-**11c**

Mixture of diastereomers
^{1}H NMR (400 MHz, $CDCl_3$)

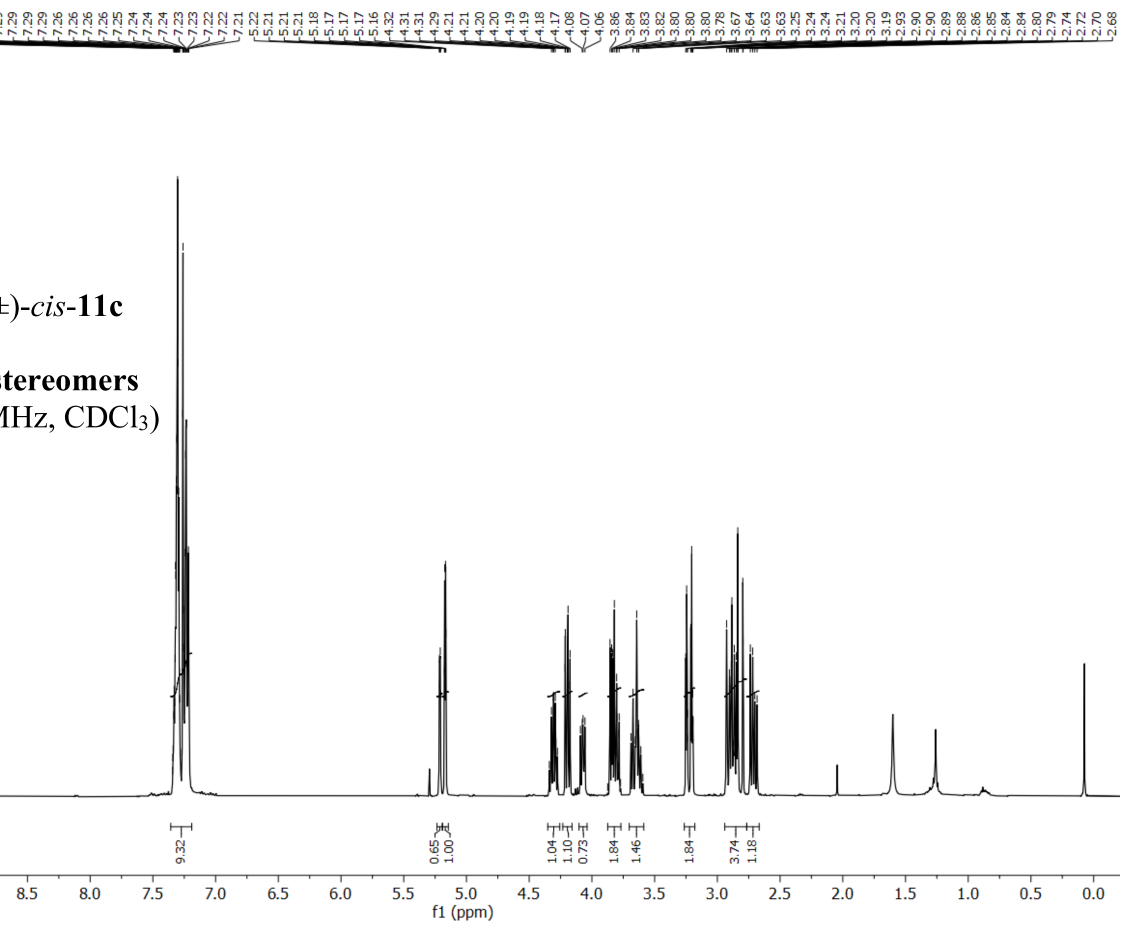

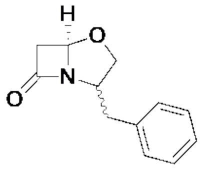

(±)-*trans*-**11c** (±)-*cis*-**11c**

Mixture of diastereomers
^{13}C NMR (100 MHz, $CDCl_3$)

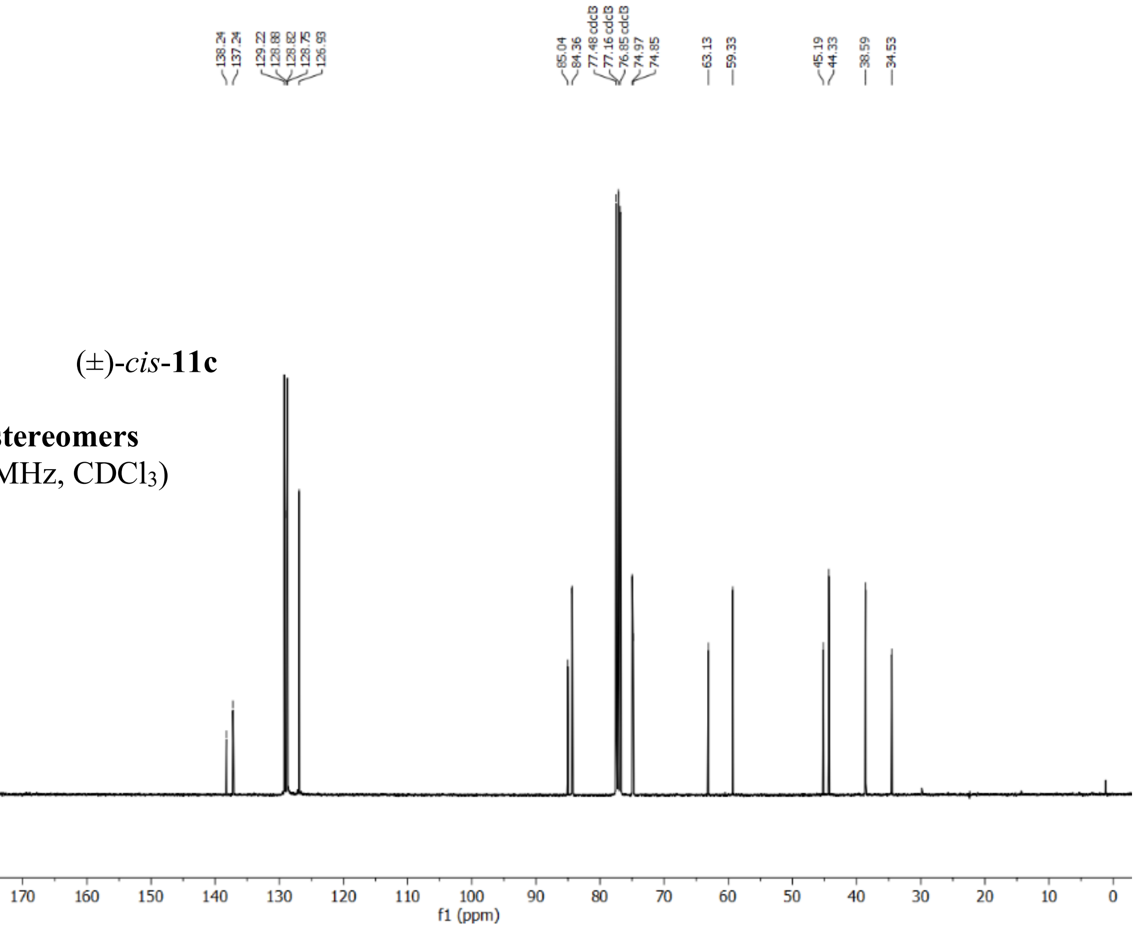

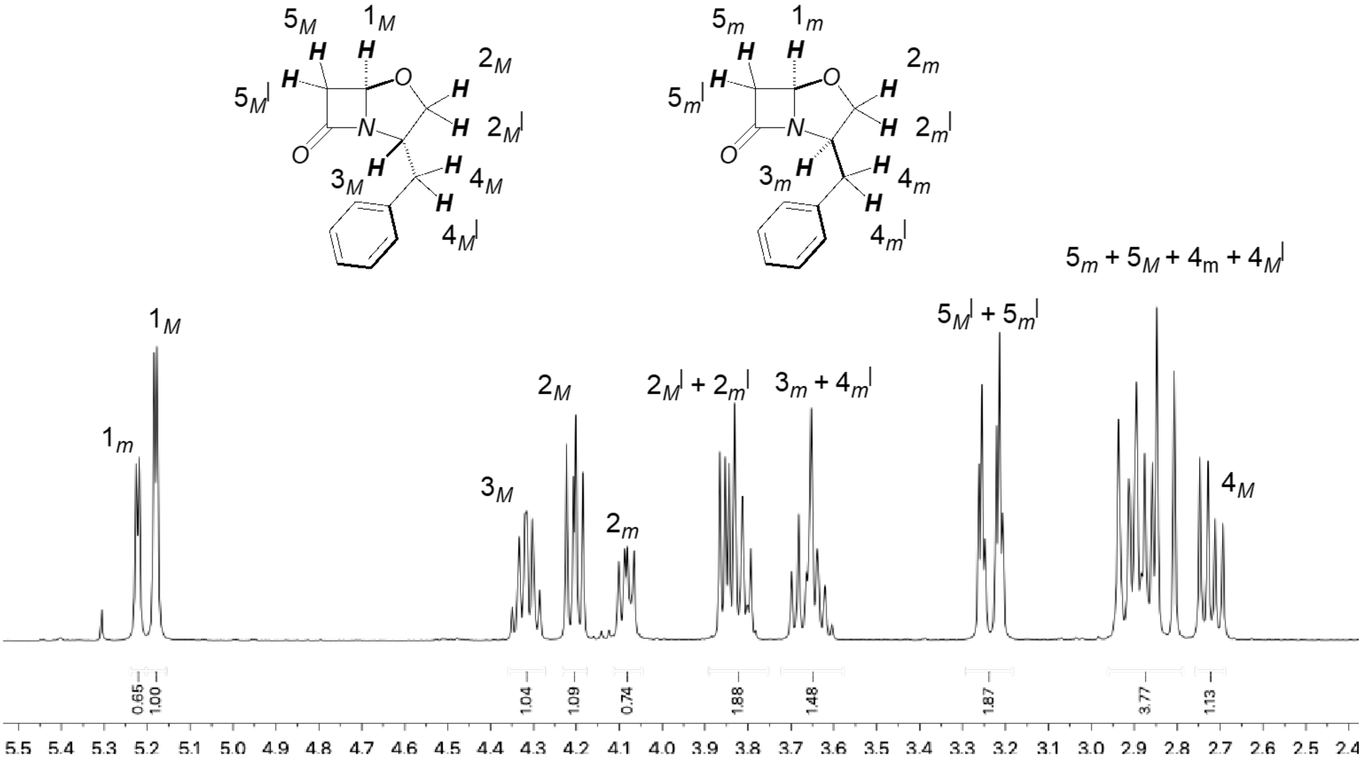
1M
1m
3M
2M
2m
2M| + 2m|
3m + 4m|
5M| + 5m|
5m + 5M + 4m + 4M|
4M
0.65
1.00
1.04
1.09
0.74
1.88
1.48
1.87
3.77
1.13
5.5 5.4 5.3 5.2 5.1 5.0 4.9 4.8 4.7 4.6 4.5 4.4 4.3 4.2 4.1 4.0 3.9 3.8 3.7 3.6 3.5 3.4 3.3 3.2 3.1 3.0 2.9 2.8 2.7 2.6 2.5 2.4

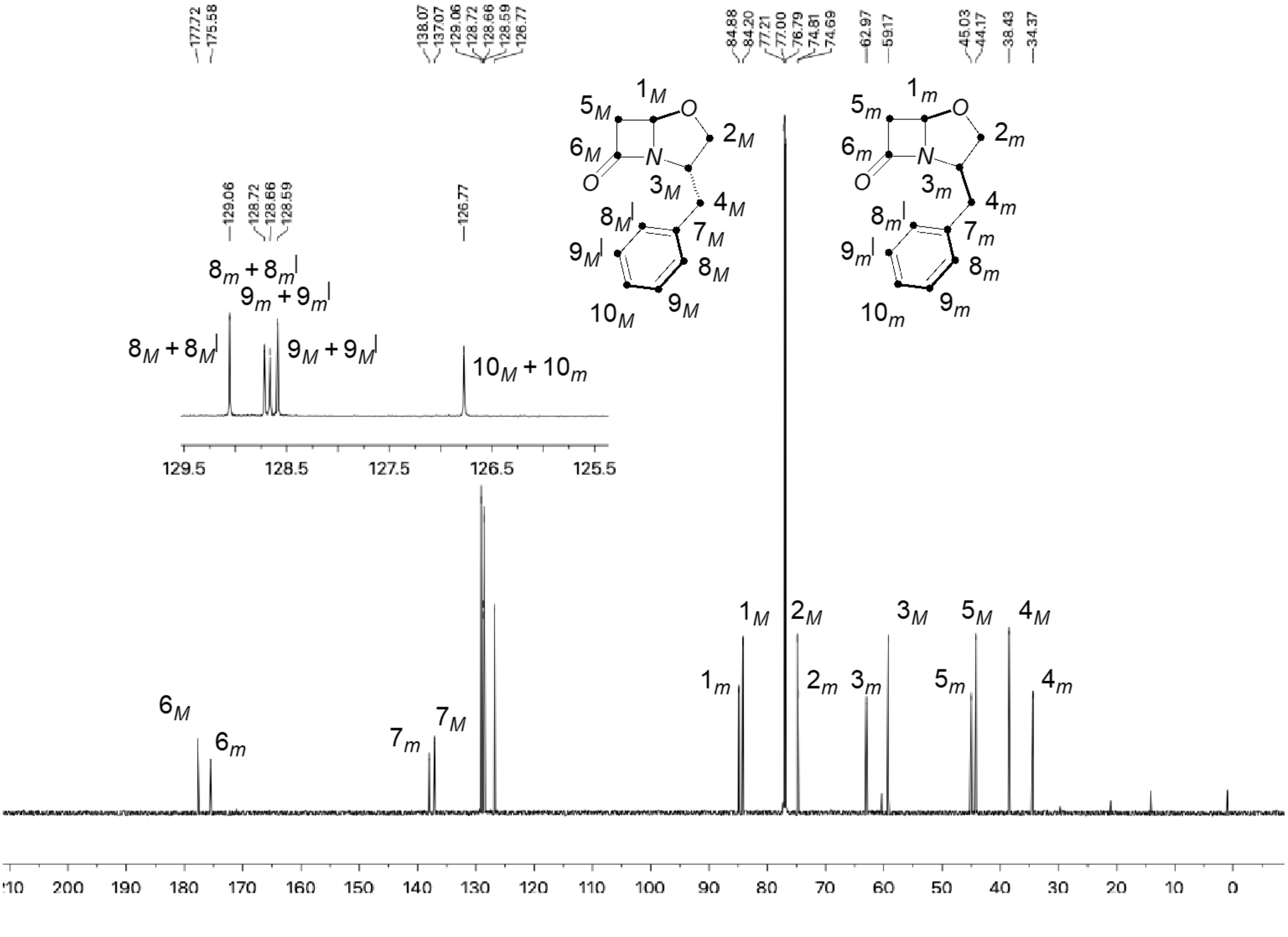
177.72
175.58
138.07
137.07
129.06
128.72
128.66
128.59
126.77
84.88
84.20
77.21
77.00
76.79
74.81
74.69
62.97
59.17
45.03
44.17
38.43
34.37
8m + 8m|
9m + 9m|
8M + 8M|
9M + 9M|
10M + 10m
129.5 128.5 127.5 126.5 125.5
6M
6m
7m
7M
1m
1M
2M
2m
3m
3M
5m
5M
4M
4m
210 200 190 180 170 160 150 140 130 120 110 100 90 80 70 60 50 40 30 20 10 0

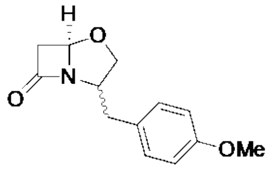

(±)-*trans*-**11d** (±)-*cis*-**11d**

Mixture of diastereomers
^{1}H NMR (400 MHz, $CDCl_3$)

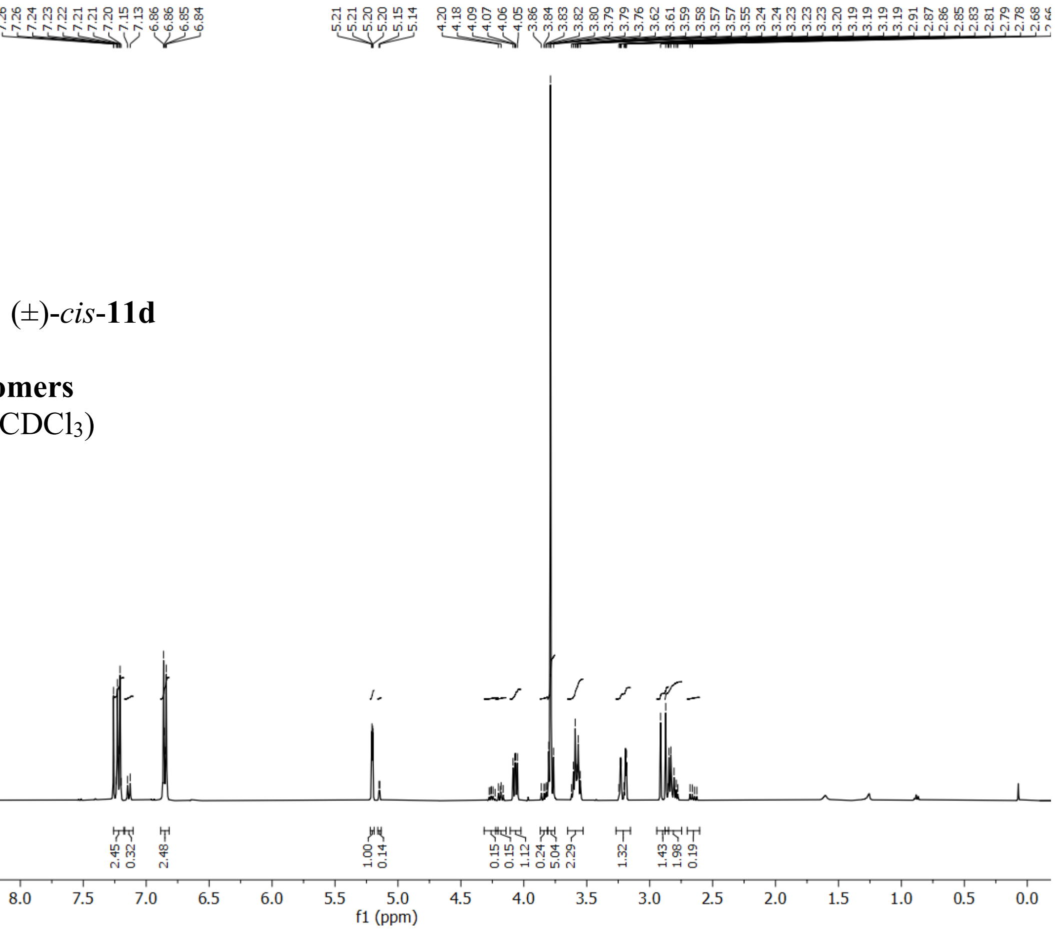

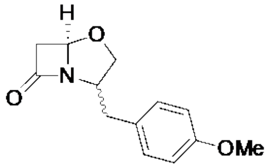

(±)-*trans*-**11d** (±)-*cis*-**11d**

Mixture of diastereomers
^{13}C NMR (100 MHz, $CDCl_3$)

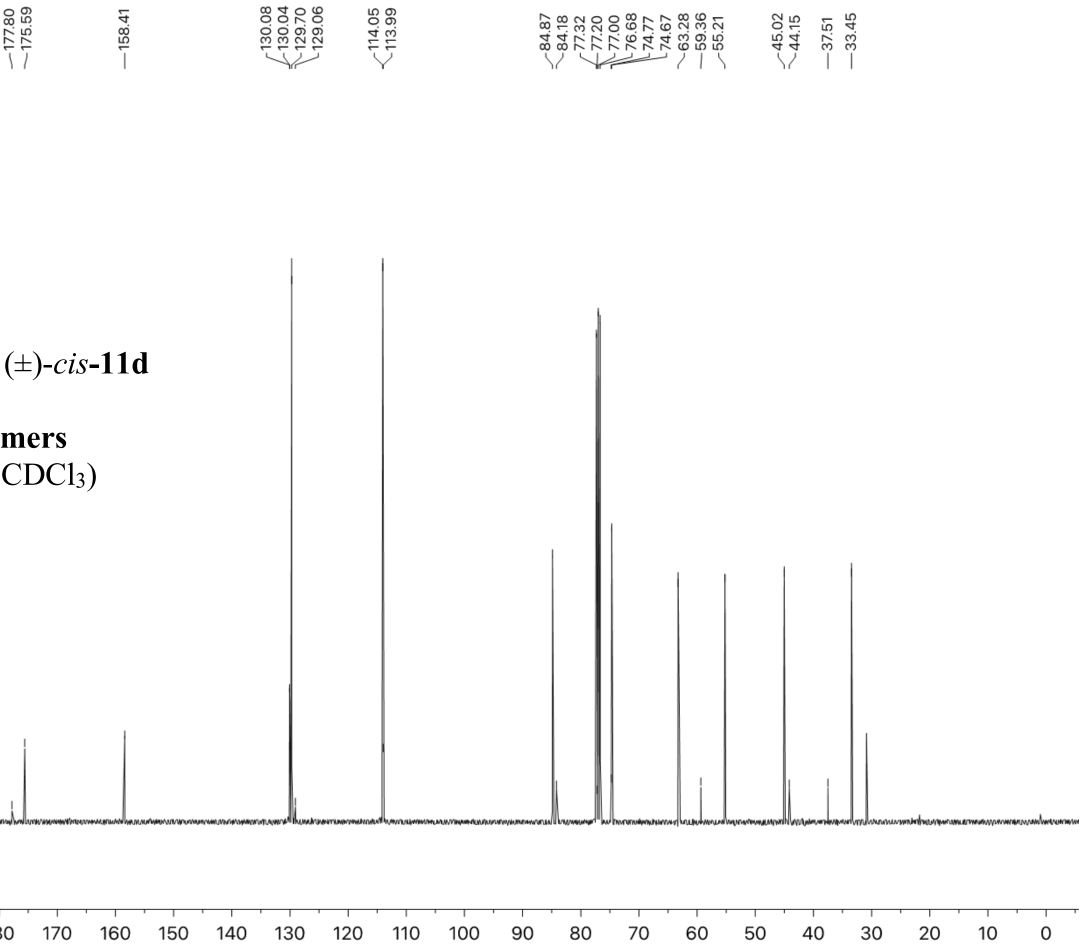

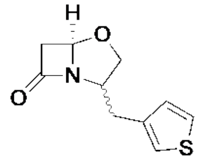

(±)-*trans*-**11e** (±)-*cis*-**11e**

Mixture of diastereomers

^{1}H NMR (400 MHz, $CDCl_3$)

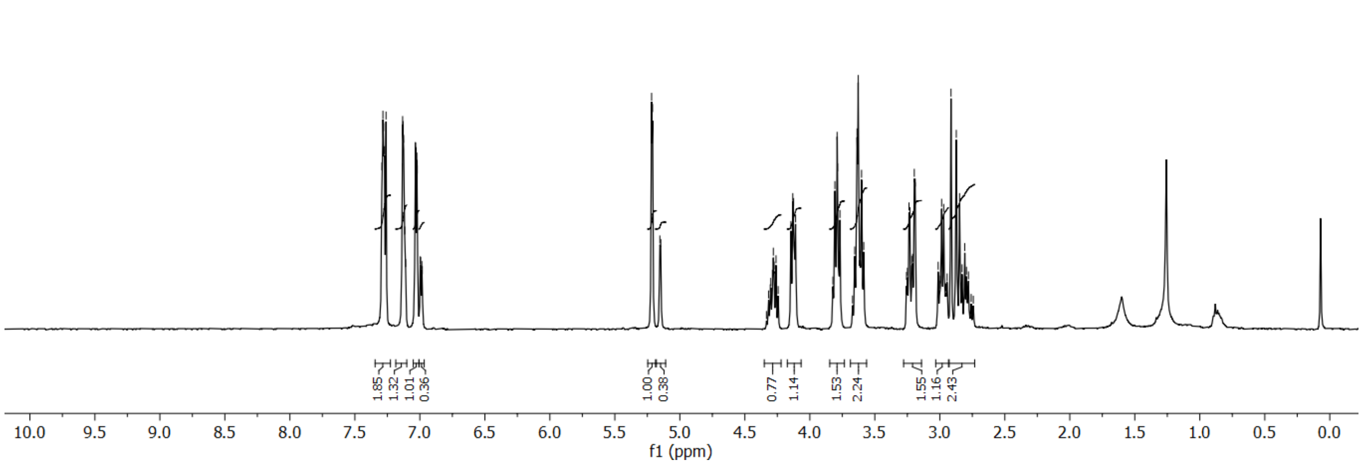

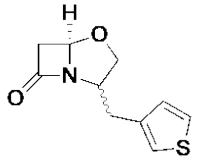

(±)-*trans*-**11e** (±)-*cis*-**11e**

Mixture of diastereomers

^{13}C NMR (100 MHz, $CDCl_3$)

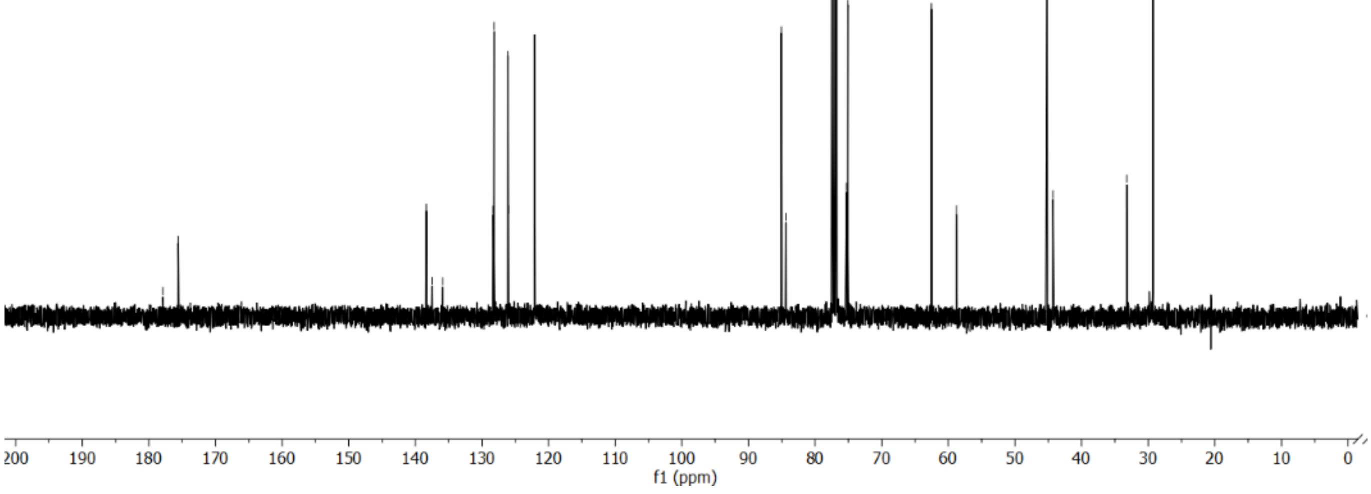

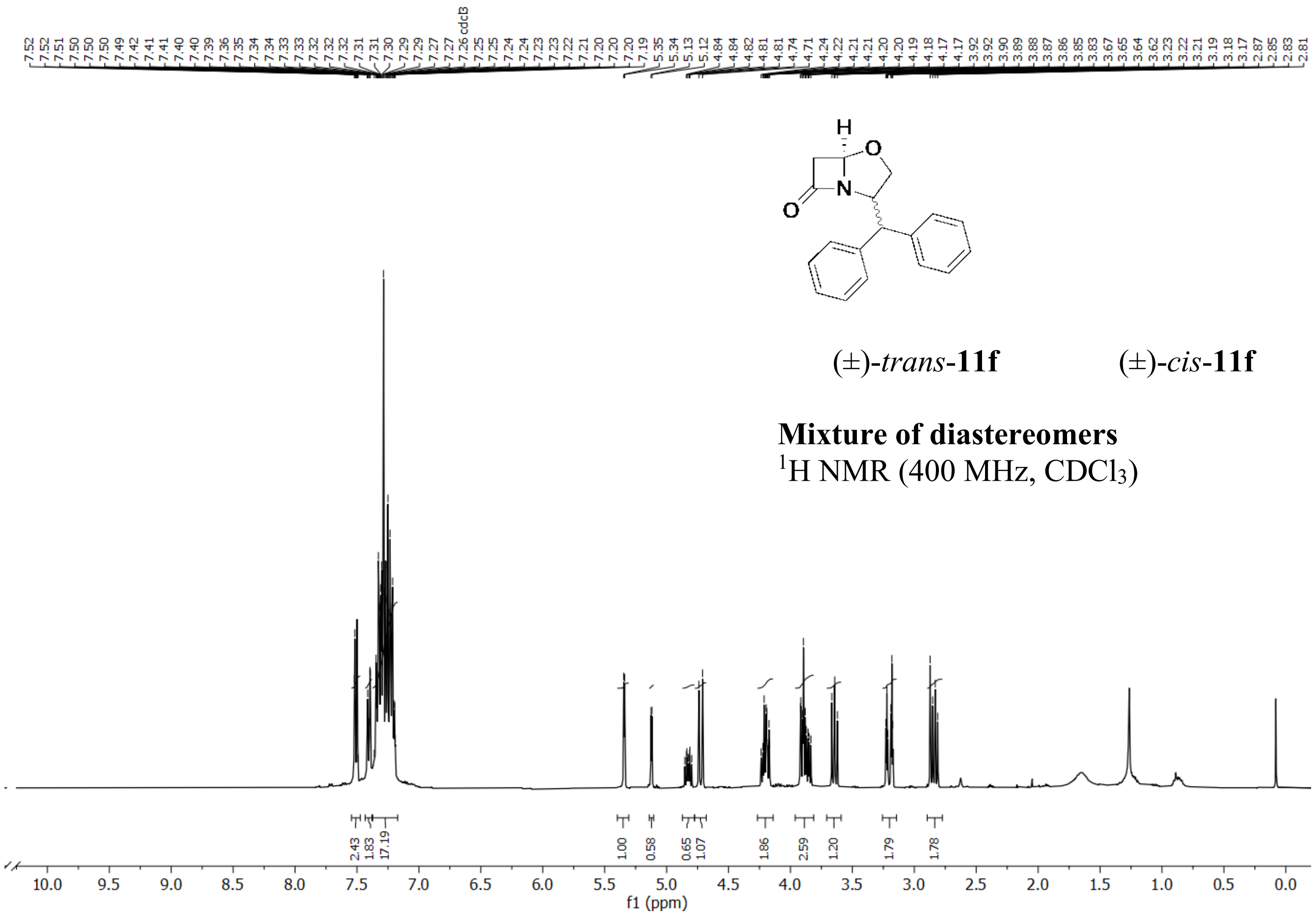
7.52 7.52 7.51 7.50 7.50 7.50 7.49 7.42 7.41 7.41 7.40 7.40 7.39 7.36 7.35 7.34 7.34 7.33 7.33 7.32 7.32 7.32 7.31 7.31 7.30 7.29 7.29 7.27 7.26 cdcl3 7.25 7.25 7.24 7.24 7.23 7.23 7.22 7.21 7.20 7.20 7.20 7.19 5.35 5.34 5.13 5.12 4.84 4.84 4.82 4.81 4.81 4.74 4.71 4.24 4.22 4.21 4.21 4.20 4.20 4.19 4.18 4.17 4.17 3.92 3.92 3.90 3.89 3.88 3.87 3.86 3.85 3.83 3.67 3.65 3.64 3.62 3.23 3.22 3.21 3.19 3.18 3.17 2.87 2.85 2.83 2.81
H
O
N
O
(±)-*trans*-**11f**
(±)-*cis*-**11f**
Mixture of diastereomers
^{1}H NMR (400 MHz, CDCl$_3$)
2.43 1.83 17.19 1.00 0.58 0.65 1.07 1.86 2.59 1.20 1.79 1.78
10.0 9.5 9.0 8.5 8.0 7.5 7.0 6.5 6.0 5.5 5.0 4.5 4.0 3.5 3.0 2.5 2.0 1.5 1.0 0.5 0.0
f1 (ppm)

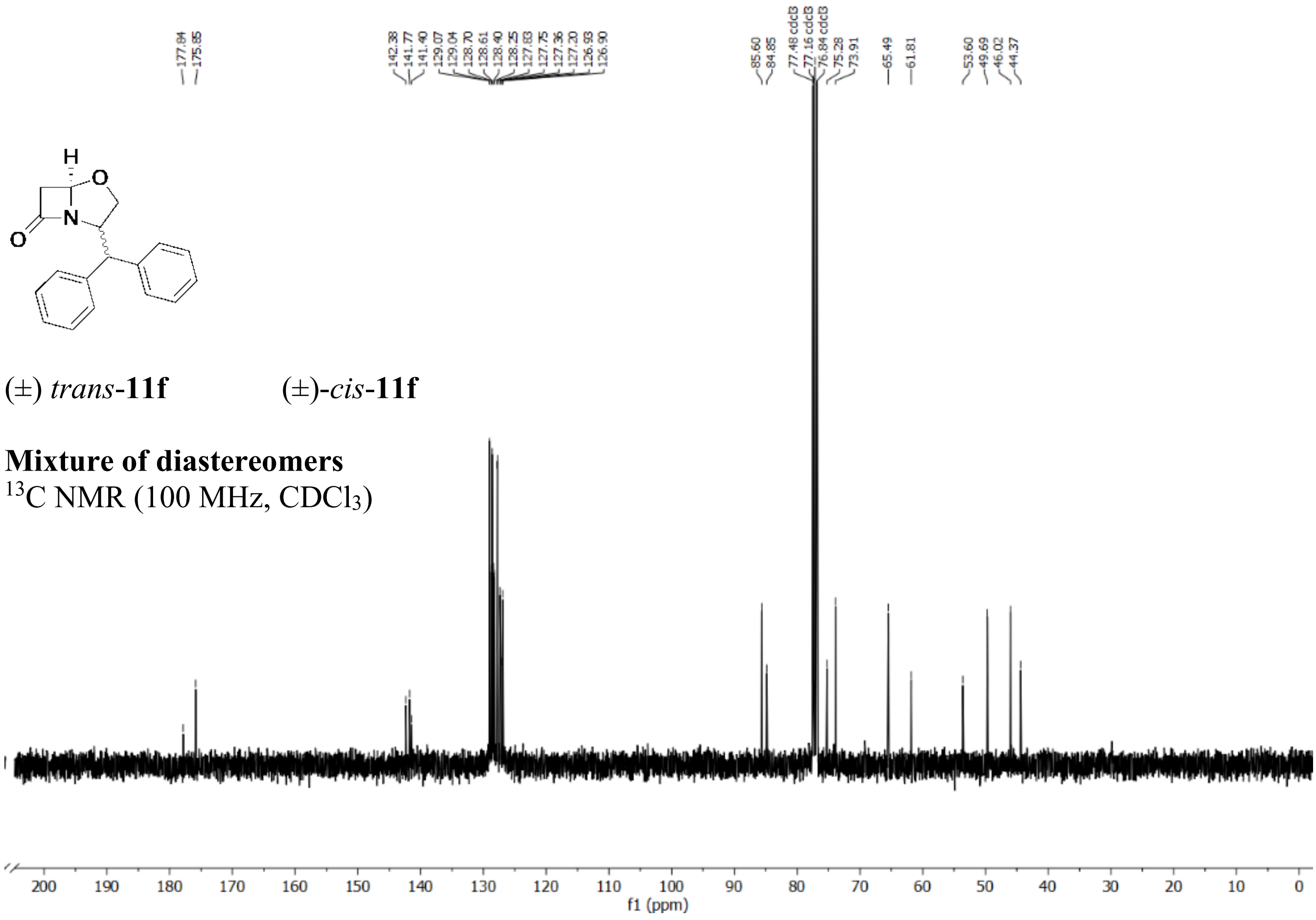
177.84 175.85
142.38 141.77 141.40 129.07 129.04 128.70 128.61 128.40 128.25 127.83 127.75 127.36 127.20 126.93 126.90
85.60 84.85 77.48 cdcl3 77.16 cdcl3 76.84 cdcl3 75.28 73.91 65.49 61.81 53.60 49.69 46.02 44.37
H
O
N
O
(±) *trans*-**11f**
(±)-*cis*-**11f**
Mixture of diastereomers
^{13}C NMR (100 MHz, CDCl$_3$)
200 190 180 170 160 150 140 130 120 110 100 90 80 70 60 50 40 30 20 10 0
f1 (ppm)

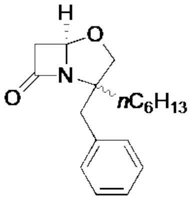

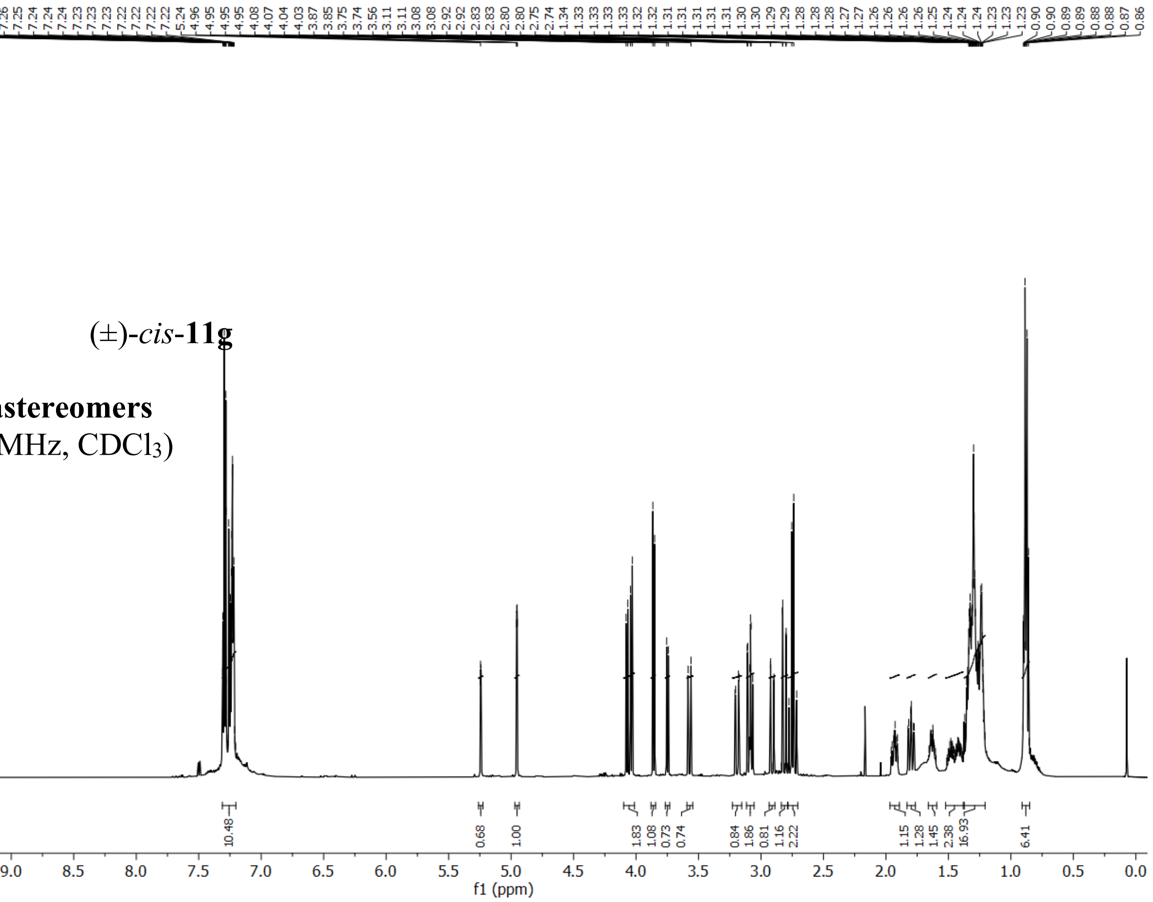

(±)-*trans*-**11g** (±)-*cis*-**11g**

Mixture of diastereomers

^{1}H NMR (600 MHz, $CDCl_3$)

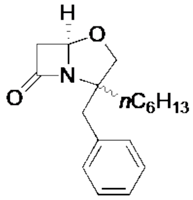

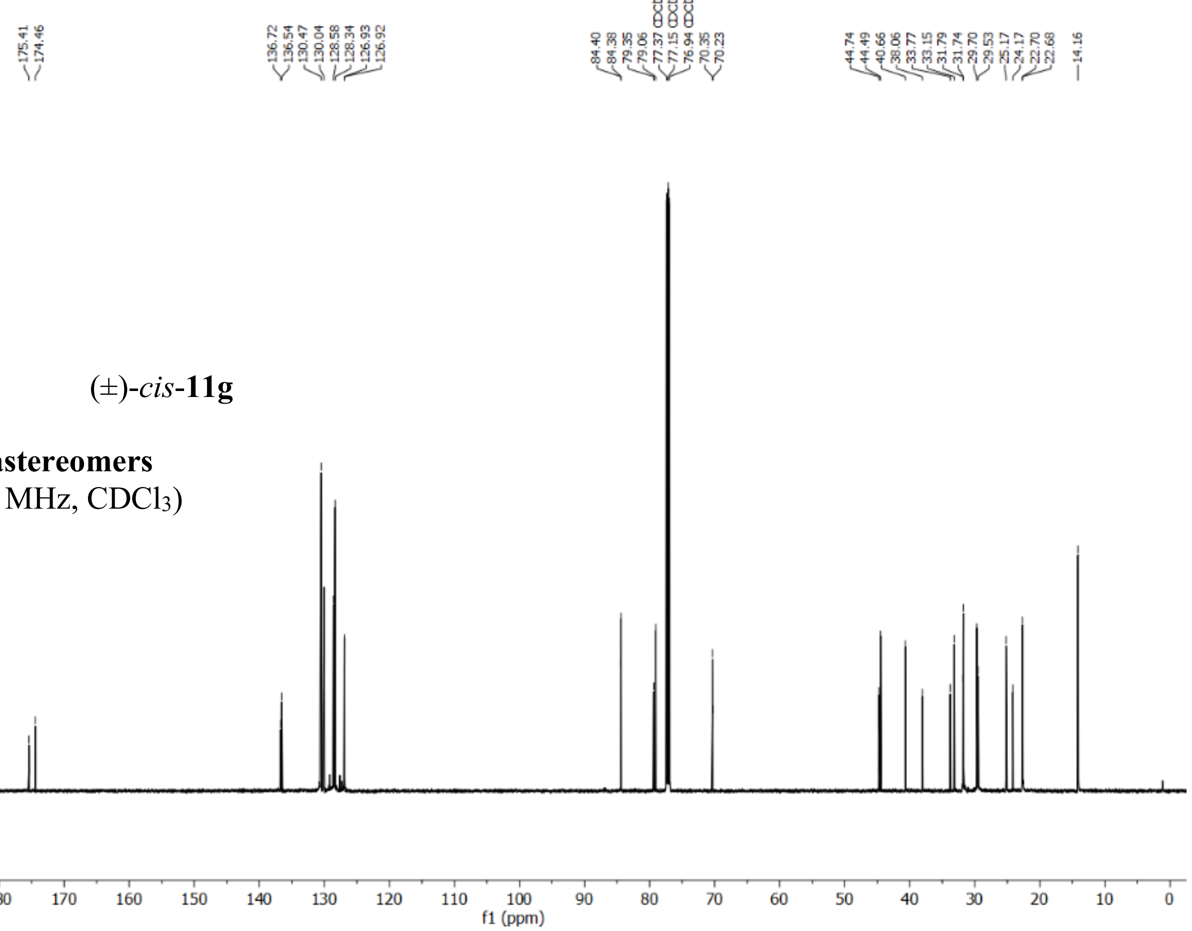

(±)-*trans*-**11g** (±)-*cis*-**11g**

Mixture of diastereomers

^{13}C NMR (151 MHz, $CDCl_3$)

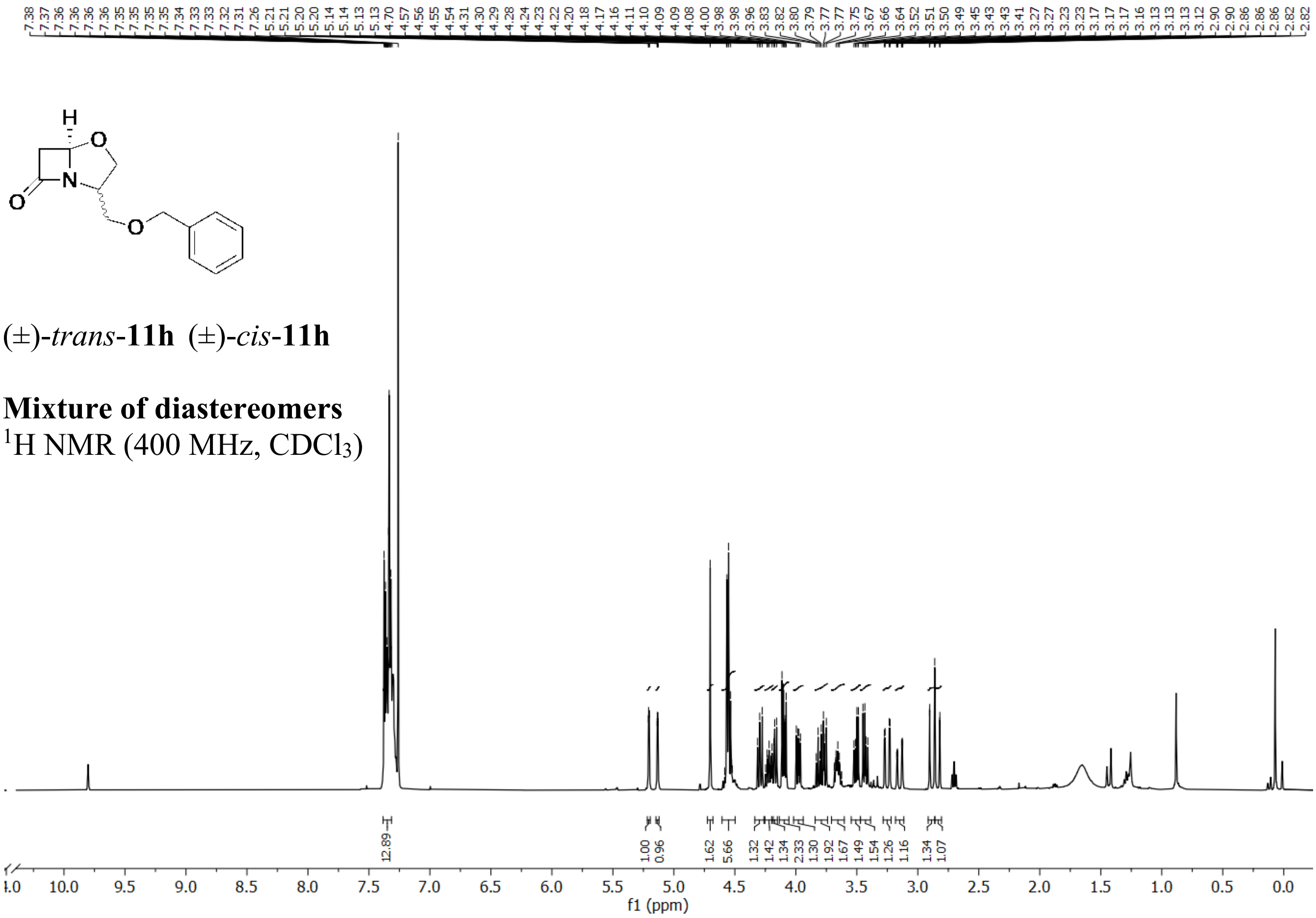

(±)-*trans*-**11h** (±)-*cis*-**11h**

Mixture of diastereomers
^{1}H NMR (400 MHz, $CDCl_3$)

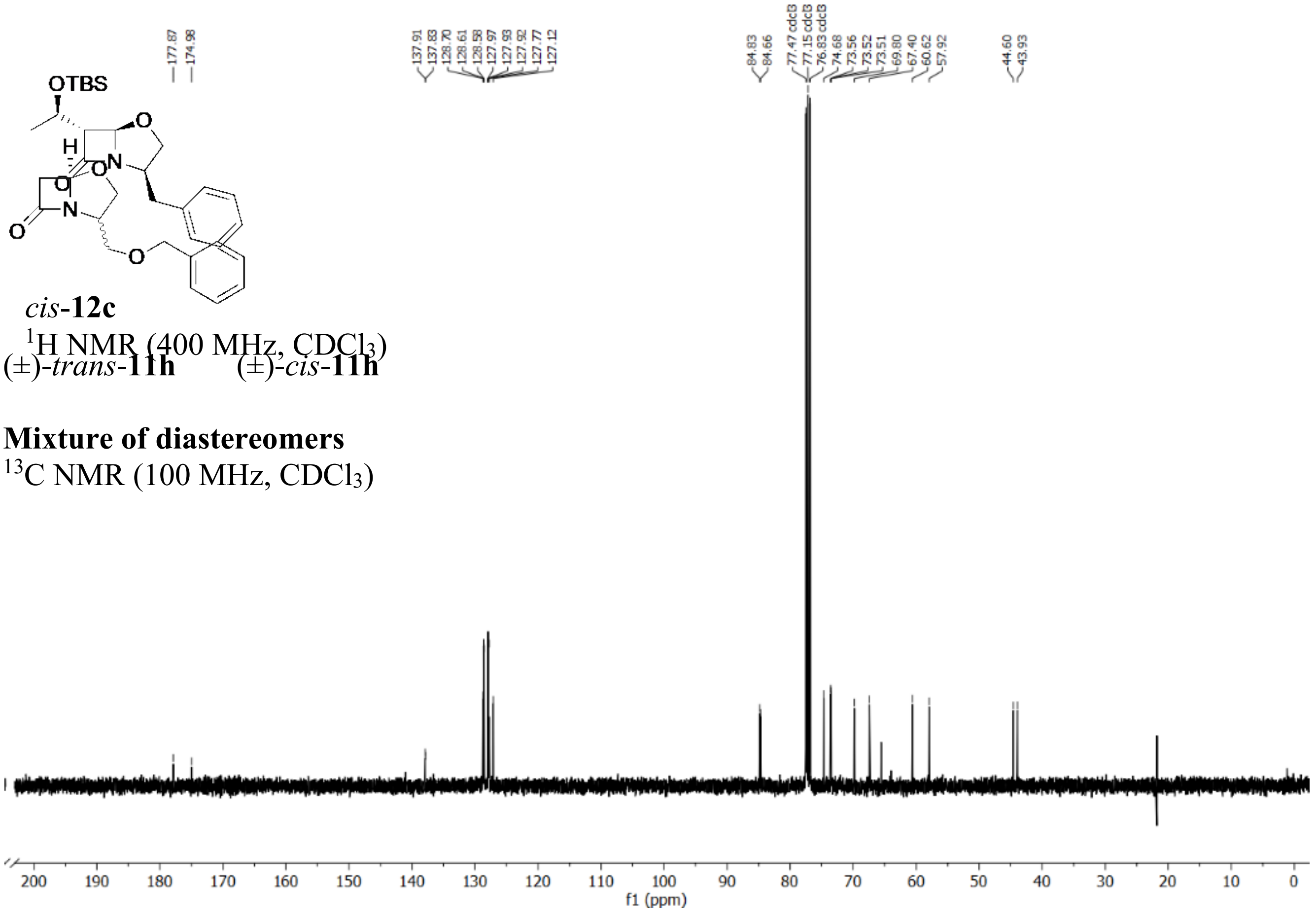

cis-**12c**
^{1}H NMR (400 MHz, $CDCl_3$)
(±)-*trans*-**11h** (±)-*cis*-**11h**

Mixture of diastereomers
^{13}C NMR (100 MHz, $CDCl_3$)

OTBS

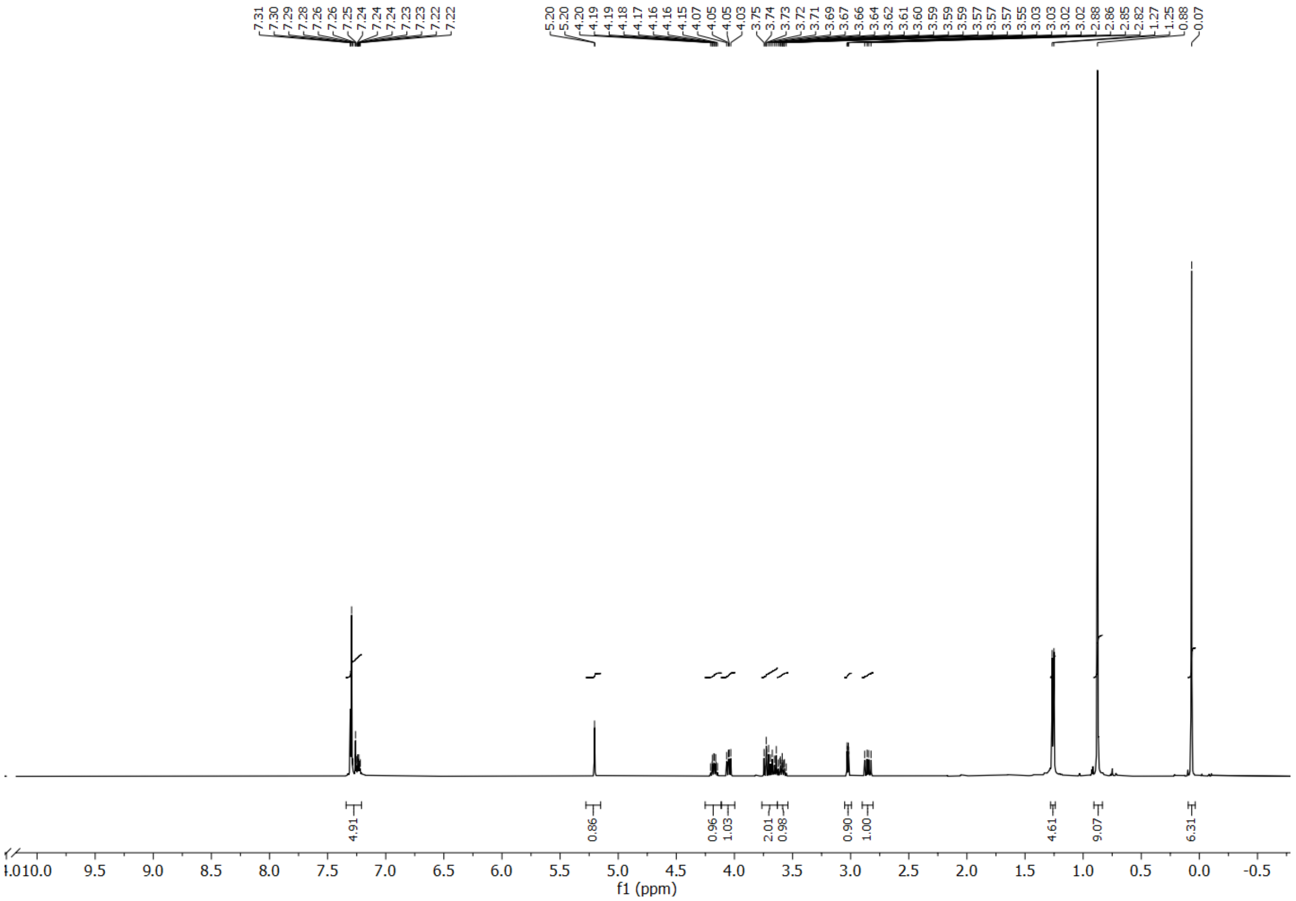
7.31 7.30 7.29 7.28 7.26 7.26 7.25 7.24 7.24 7.24 7.23 7.23 7.22 7.22
5.20 5.20 4.20 4.19 4.19 4.18 4.17 4.16 4.16 4.15 4.07 4.05 4.05 4.03 3.75 3.74 3.73 3.72 3.71 3.69 3.67 3.66 3.64 3.62 3.61 3.60 3.59 3.59 3.59 3.57 3.57 3.57 3.55 3.03 3.03 3.02 3.02 2.88 2.86 2.85 2.82 1.27 1.25 0.88 0.07
4.91
0.86
0.96
1.03
2.01
0.98
0.90
1.00
4.61
9.07
6.31
f1 (ppm)

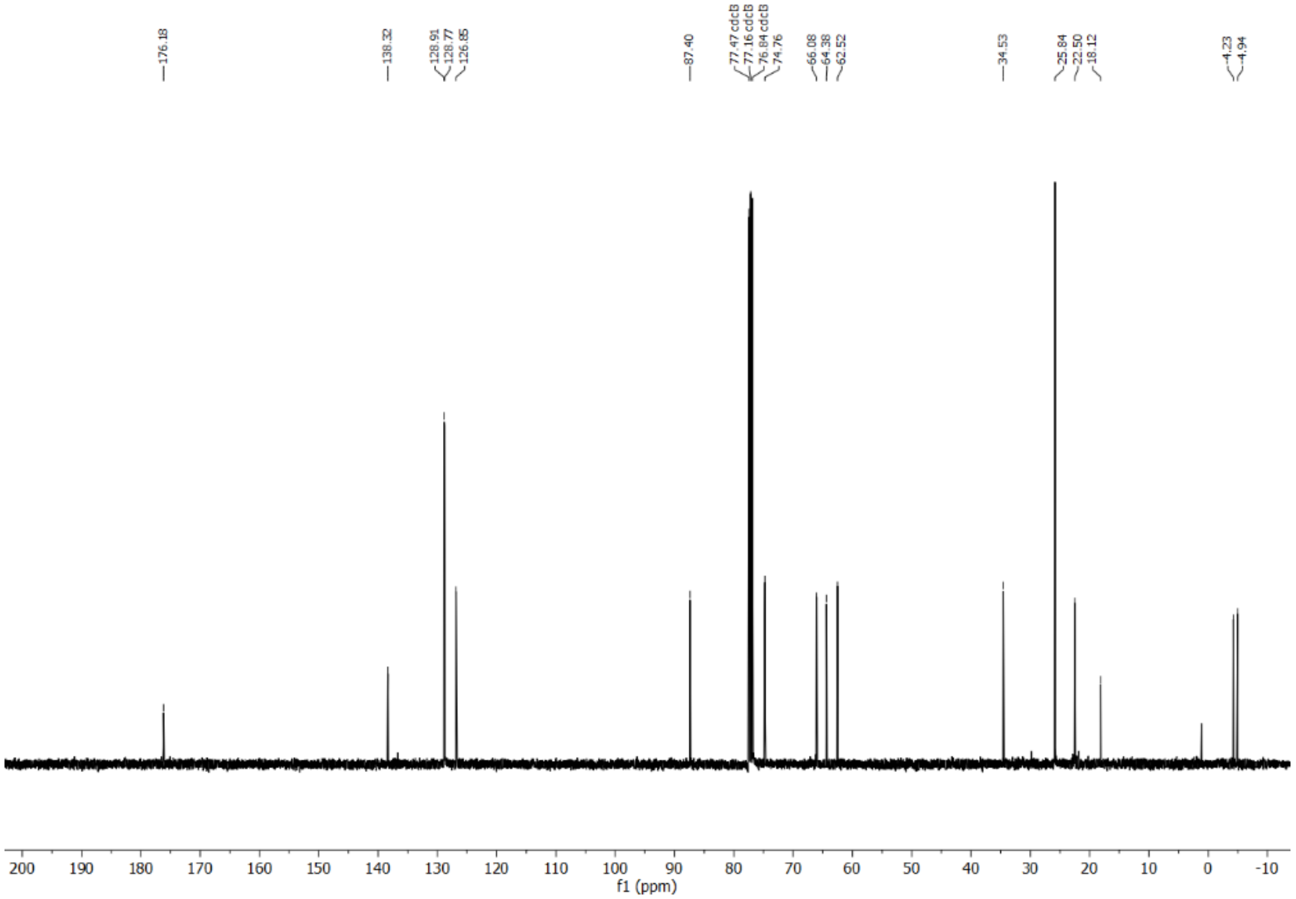
176.18
138.32
128.91 128.77 126.85
87.40
77.47 cdcl3 77.16 cdcl3 76.84 cdcl3 74.76
66.08 64.38 62.52
34.53
25.84 22.50 18.12
-4.23 -4.94
f1 (ppm)

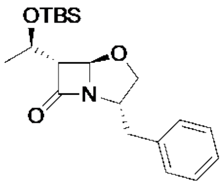

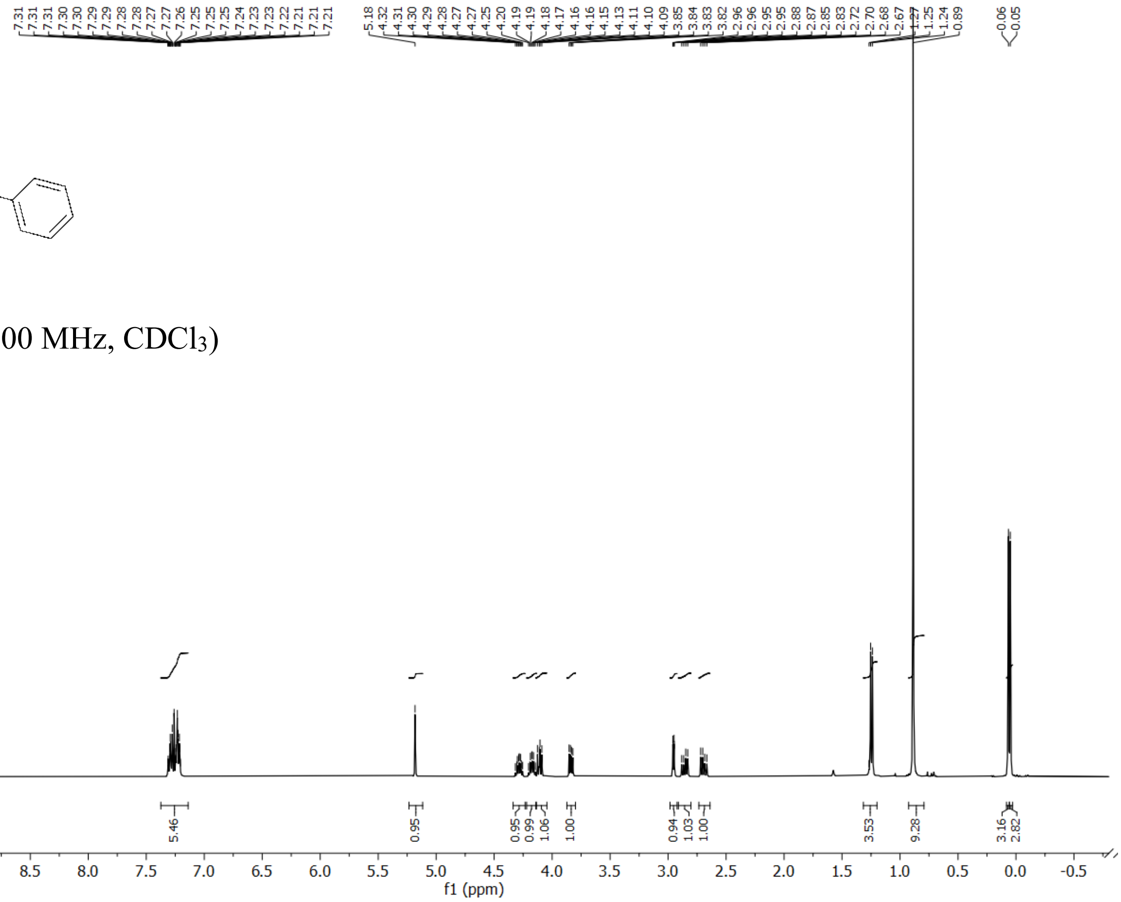

trans-**12c**

^{1}H NMR (400 MHz, $CDCl_3$)

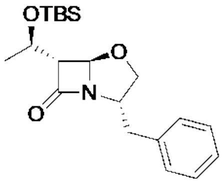

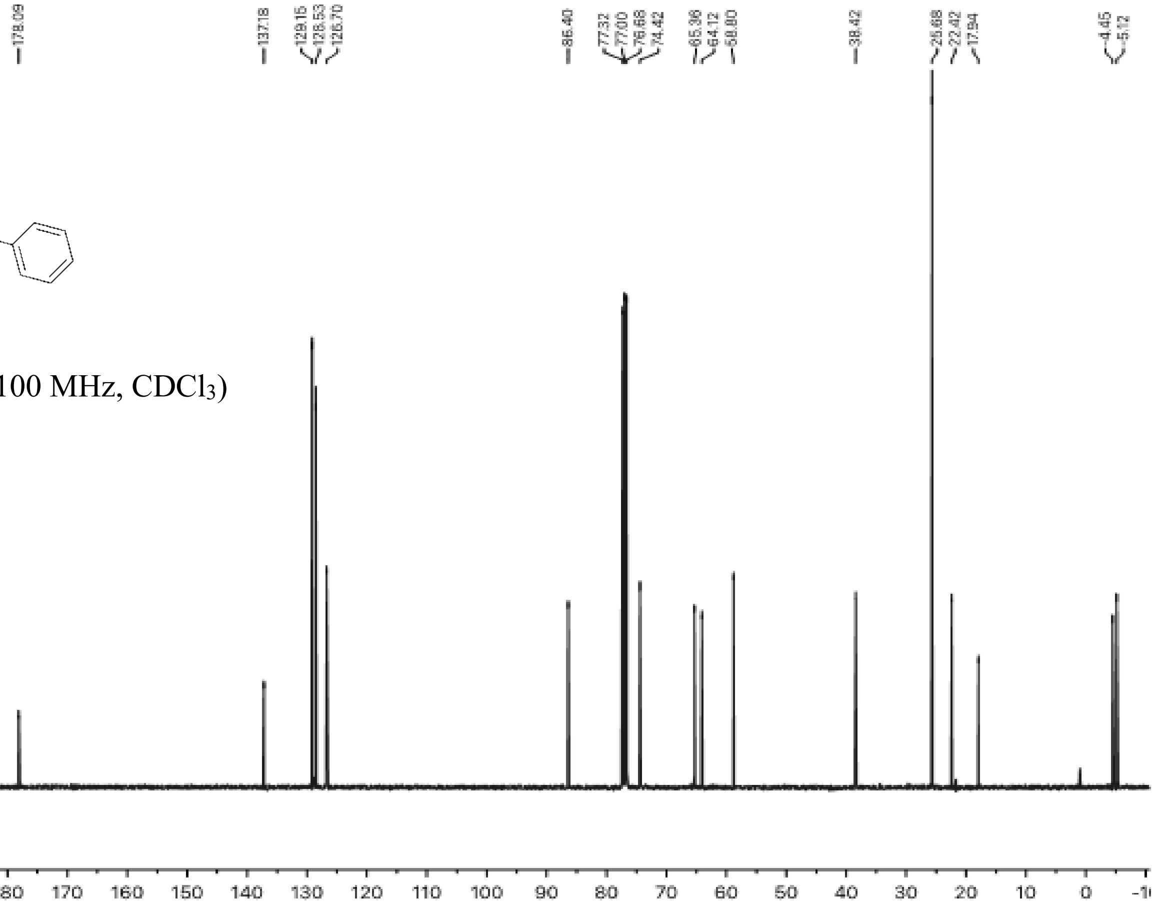

trans-**12c**

^{13}C NMR (100 MHz, $CDCl_3$)

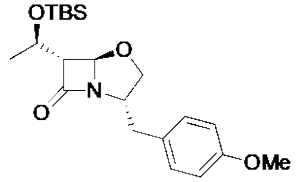

trans-**12d**
^{1}H NMR (400 MHz, $CDCl_3$)

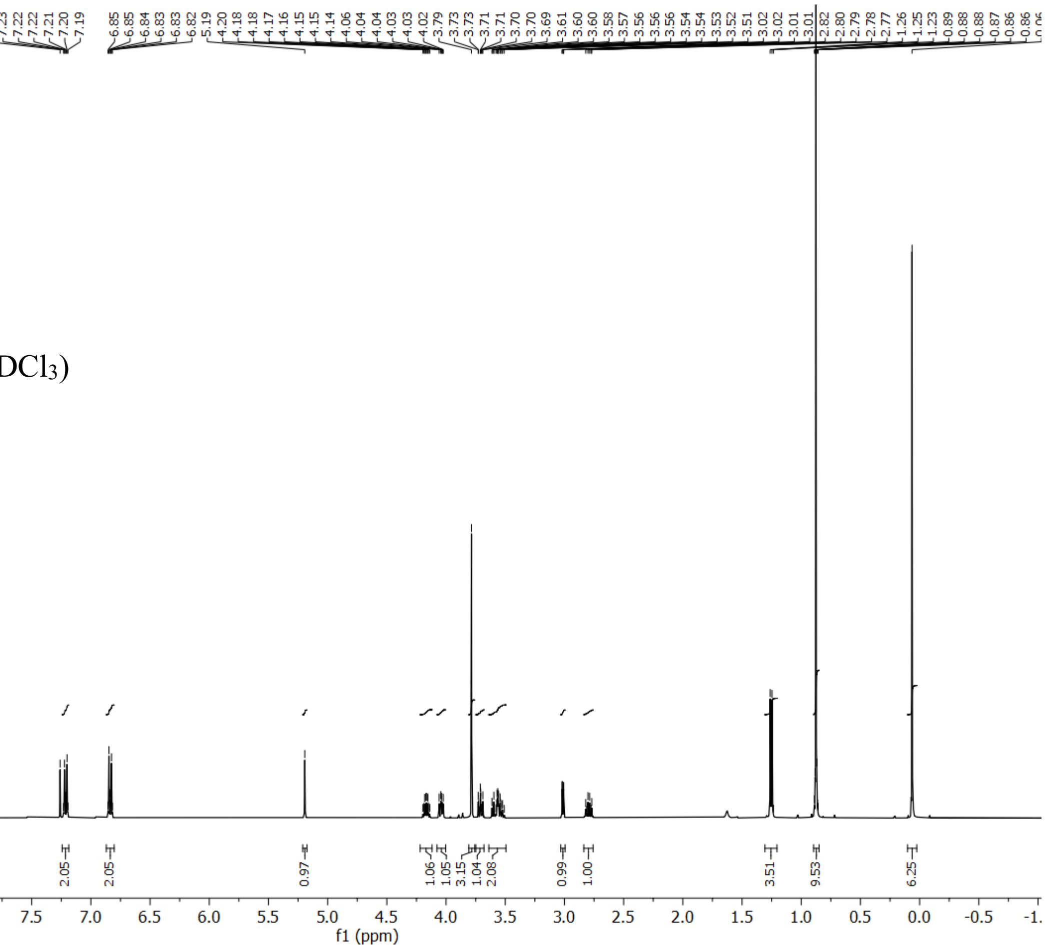

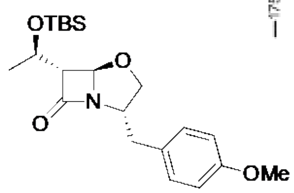

trans-**12d**
^{13}C NMR (100 MHz, $CDCl_3$)

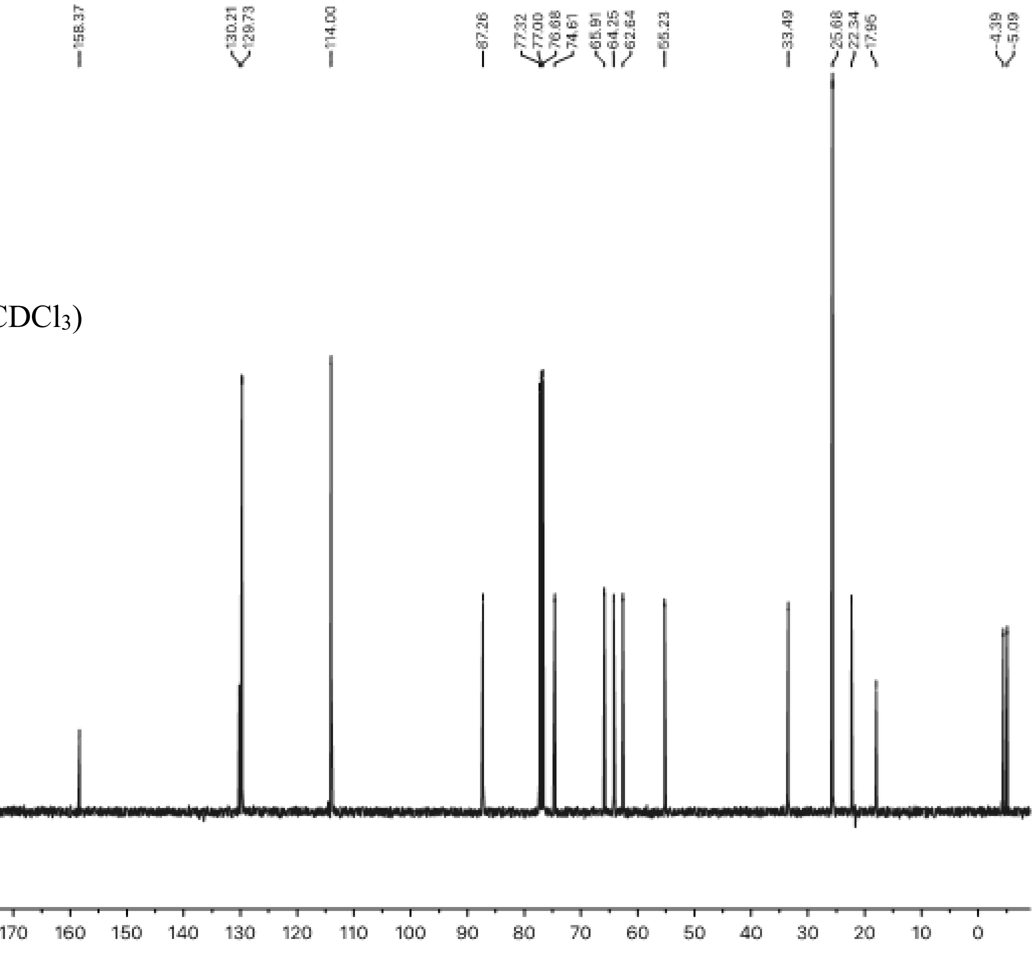

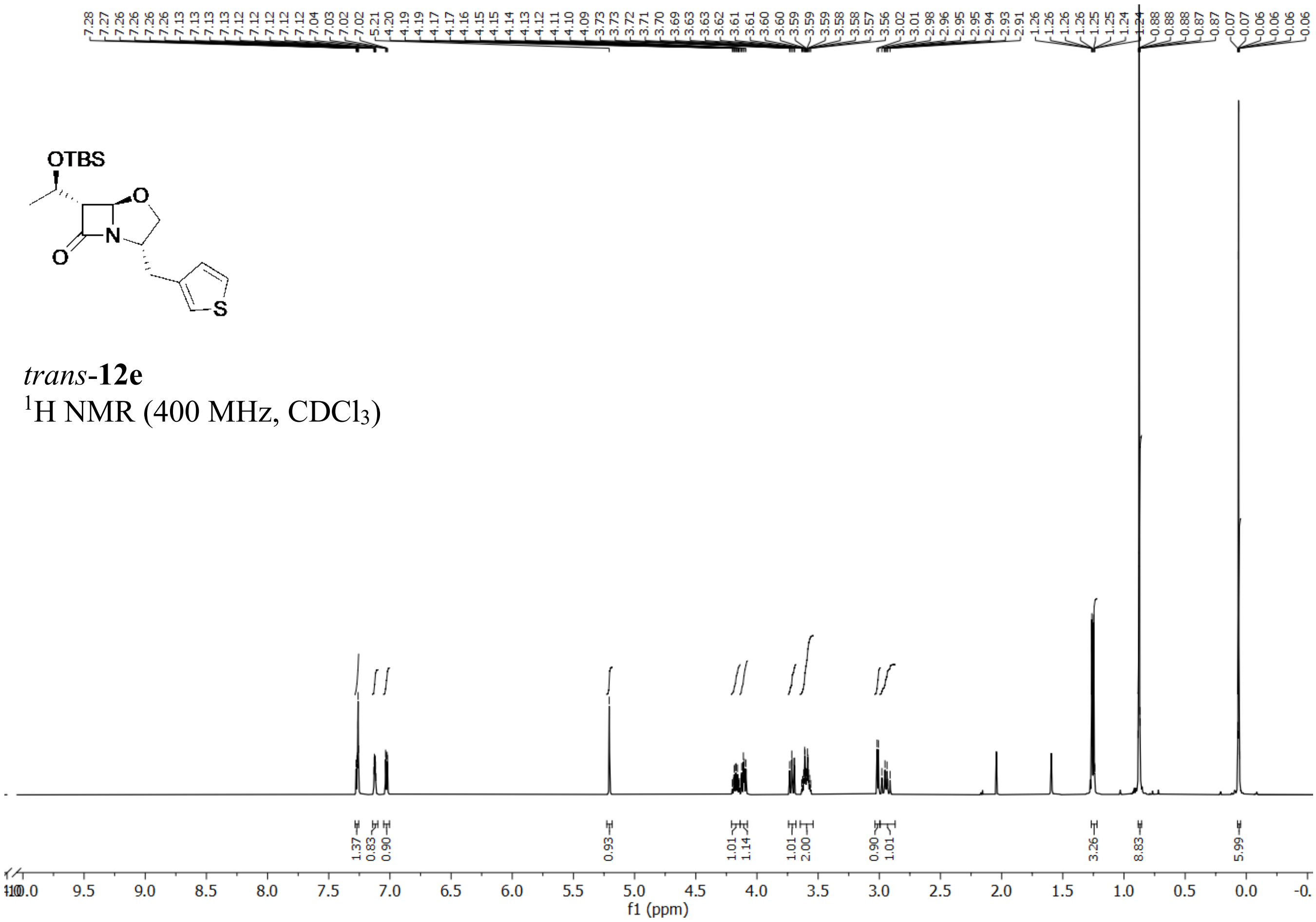
OTBS
O
N
O
S
trans-12e
1H NMR (400 MHz, CDCl3)
7.28 7.27 7.26 7.26 7.26 7.13 7.13 7.13 7.13 7.12 7.12 7.12 7.12 7.12 7.04 7.03 7.02 7.02 5.21 4.20 4.19 4.19 4.17 4.17 4.16 4.15 4.15 4.14 4.13 4.12 4.11 4.10 4.09 3.73 3.73 3.72 3.71 3.70 3.69 3.63 3.63 3.62 3.61 3.61 3.60 3.60 3.59 3.59 3.59 3.58 3.58 3.57 3.56 3.02 3.01 2.98 2.96 2.95 2.95 2.94 2.93 2.91 1.26 1.26 1.26 1.25 1.25 1.24 1.24 0.88 0.88 0.88 0.87 0.07 0.07 0.06 0.06 0.06
1.37 0.83 0.90 0.93 1.01 1.14 1.01 2.00 0.90 1.01 3.26 8.83 5.99
10.0 9.5 9.0 8.5 8.0 7.5 7.0 6.5 6.0 5.5 5.0 4.5 4.0 3.5 3.0 2.5 2.0 1.5 1.0 0.5 0.0
f1 (ppm)

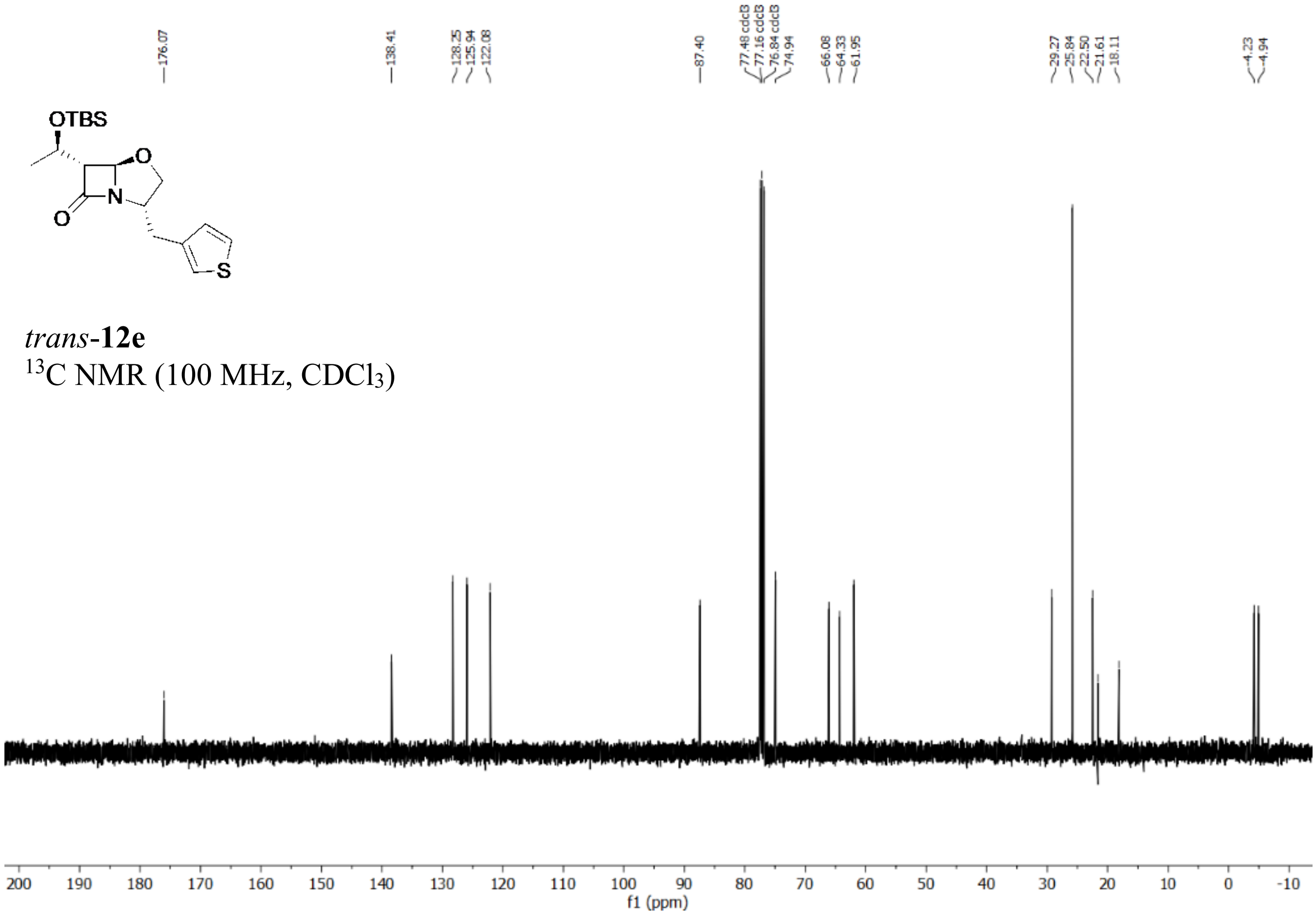
176.07
138.41
128.25 125.94 122.08
87.40
77.48 cdcl3 77.16 cdcl3 76.84 cdcl3 74.94
66.08 64.33 61.95
29.27 25.84 22.50 21.61 18.11
-4.23 -4.94
OTBS
O
N
O
S
trans-12e
13C NMR (100 MHz, CDCl3)
200 190 180 170 160 150 140 130 120 110 100 90 80 70 60 50 40 30 20 10 0 -10
f1 (ppm)

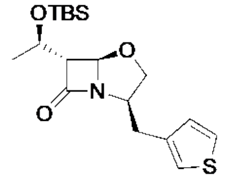

cis-**12e**
^{1}H NMR (400 MHz, $CDCl_3$)

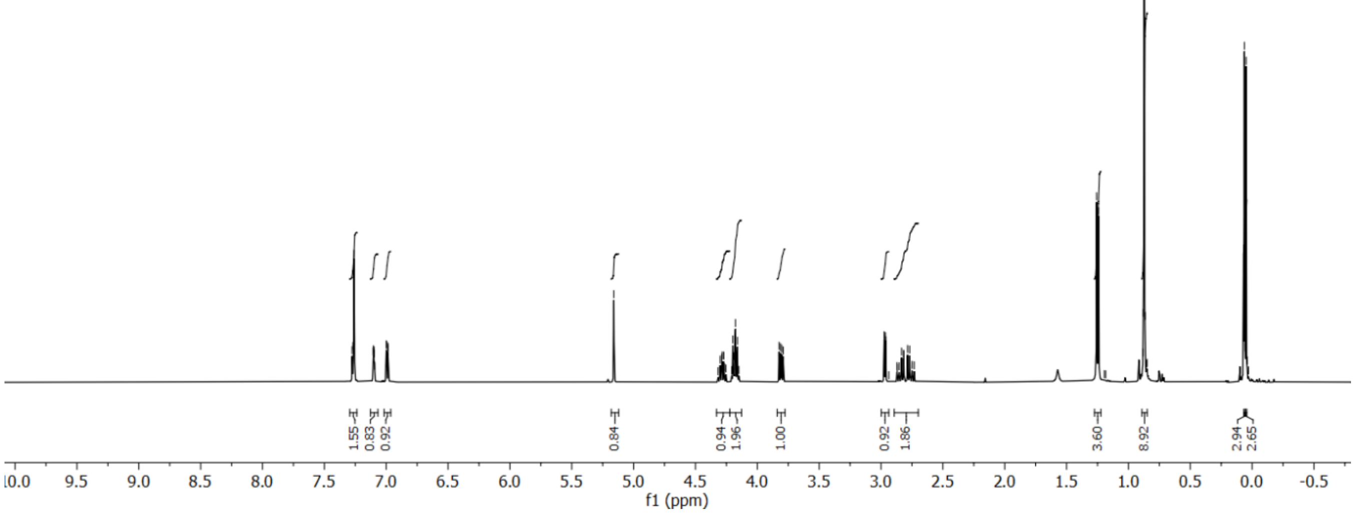

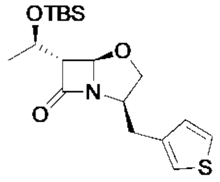

cis-**12e**
^{13}C NMR (100 MHz, $CDCl_3$)

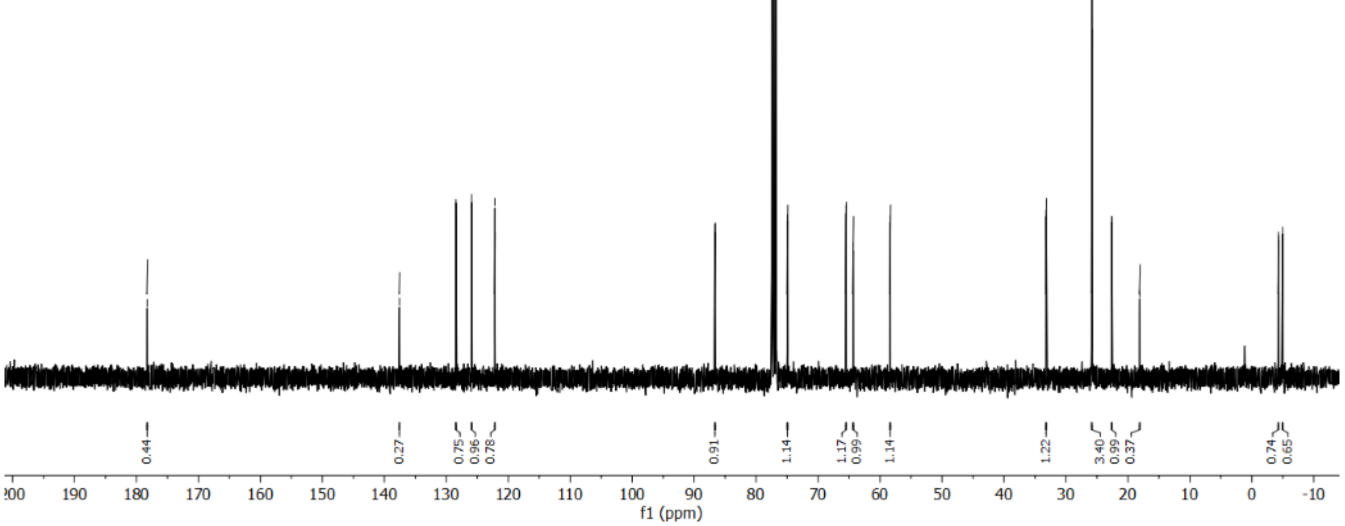

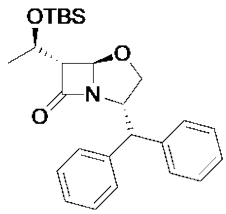

trans-**12f**

^{1}H NMR (400 MHz, $CDCl_3$)

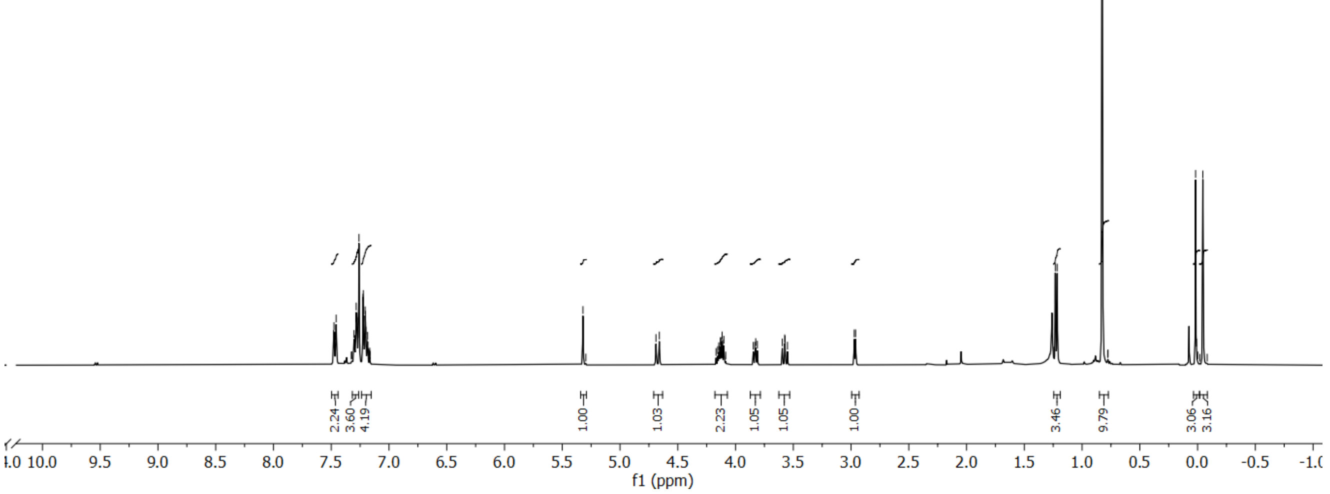

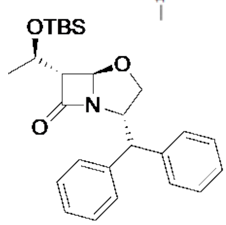

trans-**12f**

^{13}C NMR (100 MHz, $CDCl_3$)

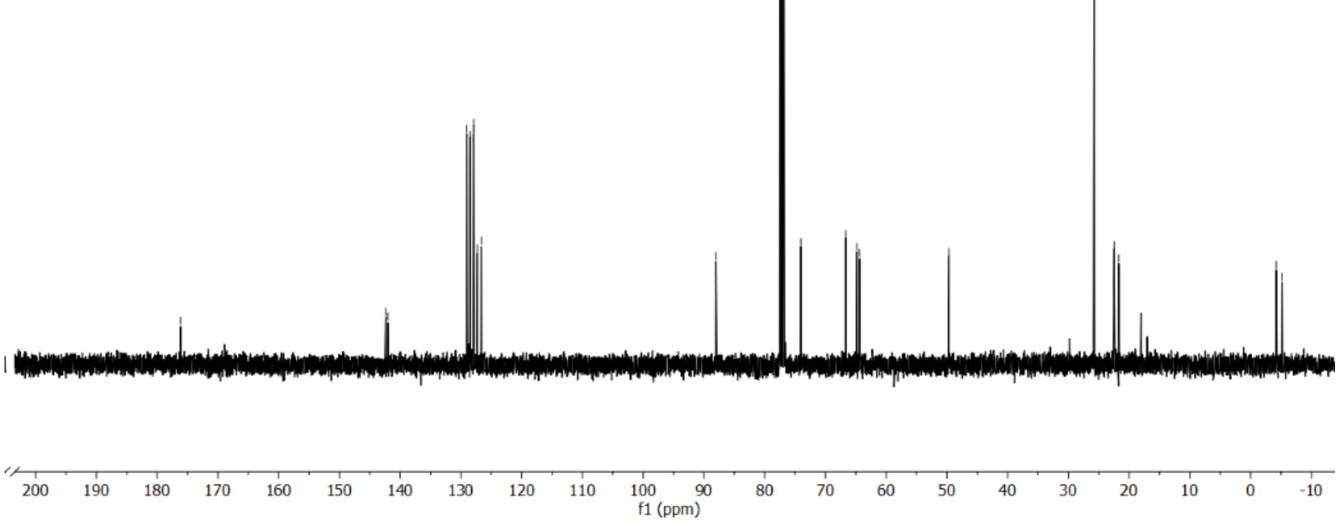

Supplement: File 1 — Reaction optimization studies, general experimental procedures, product isolation and characterization, spectroscopic data for new compounds, and copies of NMR spectra. [file Beilstein_J_Org_Chem-20-2461-s001.pdf]
